# Epitope Mapping of Exposed Tegument and Alimentary Tract Proteins Identifies Putative Antigenic Targets of the Attenuated Schistosome Vaccine

**DOI:** 10.3389/fimmu.2020.624613

**Published:** 2021-03-03

**Authors:** Leonardo P. Farias, Gillian M. Vance, Patricia S. Coulson, Juliana Vitoriano-Souza, Almiro Pires da Silva Neto, Arporn Wangwiwatsin, Leandro Xavier Neves, William Castro-Borges, Stuart McNicholas, Keith S. Wilson, Luciana C. C. Leite, R. Alan Wilson

**Affiliations:** ^1^Laboratório de Desenvolvimento de Vacinas, Instituto Butantan, São Paulo, Brazil; ^2^York Biomedical Research Institute, University of York, York, United Kingdom; ^3^Laboratório de Inflamação e Biomarcadores, Instituto Gonçalo Moniz, Fundação Oswaldo Cruz, Salvador, Brazil; ^4^Parasite Genomics, Wellcome Trust Sanger Institute, Cambridge, United Kingdom; ^5^Instituto de Ciẽncias Exatas e Biológicas, Universidade Federal de Ouro Preto, Ouro Preto, Brazil; ^6^York Structural Biology Laboratory, University of York, York, United Kingdom

**Keywords:** *Schistosoma mansoni*, epitope mapping, tegument proteins, alimentary tract, attenuated vaccine, antigenic targets

## Abstract

The radiation-attenuated cercarial vaccine remains the gold standard for the induction of protective immunity against *Schistosoma mansoni*. Furthermore, the protection can be passively transferred to naïve recipient mice from multiply vaccinated donors, especially IFNgR KO mice. We have used such sera versus day 28 infection serum, to screen peptide arrays and identify likely epitopes that mediate the protection. The arrays encompassed 55 secreted or exposed proteins from the alimentary tract and tegument, the principal interfaces with the host bloodstream. The proteins were printed onto glass slides as overlapping 15mer peptides, reacted with primary and secondary antibodies, and reactive regions detected using an Agilent array scanner. Pep Slide Analyzer software provided a numerical value above background for each peptide from which an aggregate score could be derived for a putative epitope. The reactive regions of 26 proteins were mapped onto crystal structures using the CCP4 molecular graphics, to aid selection of peptides with the greatest accessibility and reactivity, prioritizing vaccine over infection serum. A further eight MEG proteins were mapped to regions conserved between family members. The result is a list of priority peptides from 44 proteins for further investigation in multiepitope vaccine constructs and as targets of monoclonal antibodies.

## Introduction

The production of a schistosome vaccine would be a valuable addition to the toolbox of measures currently being used to control and ultimately to eradicate schistosomiasis, but decades of research towards that goal have yielded only a meagre harvest. The first human vaccine trial, with ShGST (1) ended with an inconclusive outcome while at least three other single antigen formulations are in early Phases of trials (2). Against this background, the vaccination of rodents and primates with Radiation-Attenuated (RA) cercariae remains a well-established means of inducing specific acquired immunity. By judicious manipulation of adjuvants (e.g. IL-12) it is possible to drive protection towards high levels (3, 4), although sterile immunity has never been achieved. Only Smp80 calpain has come anywhere near to emulating the protection achieved by the RA vaccine (5). Schistosomes undergo a protracted migration from the skin infection site to the hepatic portal system, entirely in the bloodstream, with one to several circuits of the vasculature (6). In mice only ~32% of penetrant cercariae reach maturity, while in primates >80% may mature (7). The lungs are a particular obstacle to migration and the difference in maturation has been attributed to the relative fragility of lung capillaries. The effect of the RA vaccine is to amplify the probability of migrating schistosomula getting stuck in the lungs on each pass (P ~ 0.36 in a naive mouse; P ~ 0.53 with 40% protection; P ~ 0.72 with 70% protection). Much has been discovered about the underlying immunology (8, 9). Inflammatory foci form around the larvae as they make strenuous efforts to traverse the capillary beds, impeding their progress and even deflecting them into alveoli (10). In contrast, there is little concrete information about the nature of the mediating antigens emanating from the live schistosomula targets (11).

Beginning in the 1980s, strenuous efforts were made to characterize the composition and antigenicity of the schistosome tegument, as a major parasite interface with the host, and potential target of immune attack. Techniques included biosynthetic labeling with ^35^S methionine and iodination of surface proteins, linked to immunoprecipitation and separation of proteins on 1D and 2D gels (12). Isolated membranes of the adult worm tegument (13) were also used to probe both human (14) and rodent (15) responses to schistosome infection, using western blotting. These approaches identified many reactivities, and clear differences were observed between infection and vaccination serum (12). However, at the time there was no simple and direct way to establish the identities of targets; this proceeded by isolation of one protein at a time, e.g., the Sm200 tegument surface protein (16). Rapid and comprehensive identification of proteins on gels and blots had to wait for the availability of a large transcriptome database (17), which facilitated the application of proteomics to schistosomes (18), especially to tegument proteins (19, 20). The biosynthetic radio-labeling approach applied to schistosomula revealed a characteristic pattern of proteins secreted during culture to the lung stage (21) but the dominant band was only identified by proteomics 17 years later, as a mixture of micro exon gene (MEG)-3 family proteins (22); the origin of these proteins in the head gland and tegument was confirmed by *in situ* hybridization (23). However, other secreted proteins of the migrating schistosomulum (21) remain to be identified directly by proteomics and can only be inferred from transcript data (24, 25).

Two new approaches, systems biology analysis and epitope mapping with peptide arrays have recently emerged as tools to aid in vaccine development. Systems biology was first applied to the mechanisms underlying vaccine-induced immunity in infections as diverse as yellow fever, influenza and malaria (26–29) and we have recently used it to analyze the immune processes associated with the RA schistosome vaccine in mice (30). We found that the failure to deploy the normal mechanisms for downregulation of hemostasis and coagulation after vaccination may explain parasite blockade in the lungs. Genes encoding chemokines and their receptors were also more prominent in vaccinated mice, indicating an enhanced capacity for inflammation. Both changes could potentially impact on intravascular migration. Epitope mapping has been made feasible by the laser-printing of overlapping 15mer peptides covering target protein sequences onto glass slides for screening with immune sera (https://www.pepperprint.com/). The approach has been used to identify epitopes in infectious agents as diverse as viruses, bacteria, protozoa and helminths (31–34). Recently, the technology was applied to map the epitopes present in 32 *S. japonicum* esophageal gland proteins using macaque, rabbit and mouse sera, and identified a significant number of immunodominant sequences for incorporation into vaccine constructs (35). The current report is a companion study to the systems biology analysis (30) using sera generated from vaccinated and infected C57Bl/6 mice, supplemented by high titer serum from IFNgR KO mice (36), to screen peptide arrays for reactivity to 55 secreted and surface-exposed proteins of *S. mansoni*. We mapped the reactive regions of 26 array proteins onto 3D crystal structure homologues, and for eight MEG proteins lacking solved crystal structures, to conserved regions. This has enabled the selection of a panel of epitopes, based on rational criteria, for incorporation into multi-epitope vaccine constructs and to serve as targets for monoclonal antibody production.

## Material and Methods

### Ethics Statement

The mouse sera for array screening came from Instituto Butantan, Sao Paulo, Brazil (C57Bl/6) and University of York, York, UK (IFNgR KO mice). Both were derived from or based on previously described research (30, 36, 37). Brazil procedures were conducted in strict accordance with good practices as defined by the Committee for the Ethical Use of Animals in Experimentation of the Butantan Institute (São Paulo, Brazil) under license 1030/13. York procedures were carried out in accordance with the UK Animals (Scientific Procedures) Act 1986, authorized on personal and project licenses to RAW and PSC, issued by the UK Home Office. The study protocol was approved by the Biology Department Ethical Review Committee at the University of York.

### Details of Sera

C57Bl/6 mice: Full details of the experiments can be found in (30). Vaccination serum (V) came from mice given three exposures of 500 radiation-attenuated cercariae 4 weeks apart *via* the shaved abdomen, sampled 4 weeks after the last exposure. Infection serum (I) came from mice exposed to 500 normal cercariae *via* the same route, also sampled at 4 weeks, before the start of egg deposition. Control serum came from uninfected sentinel mice kept under the same conditions as the test mice for the duration of the experiment. Three pools of vaccinated and infected serum, each from three mice (equalized by final worm burden), and two samples of control serum were analyzed. The 3x vaccinated mice showed a 70% protection against a percutaneous challenge with 120 normal cercariae (30).

IFNgR KO mice: Three experiments were performed to generate the sera used for array screening. Mouse maintenance and breeding, and experimental details of vaccination were precisely as given in Wilson et al. (36). In experiment MT1, groups of seven C57Bl/6 and IFNgR KO mice received one, two or three exposures to 500 radiation-attenuated cercariae *via* the shaved abdomen. At 5 weeks after the last exposure the test mice, along with seven naïve controls for each group, were subjected to percutaneous challenge with 200 normal cercariae, with worm burden and % protection determined by portal perfusion performed 5 weeks later. The levels of schistosome-specific IgG1 and IgG2a in the sera at various time points were determined by ELISA with soluble worm proteins as the coating antigen, as previously described (38). In parallel with the above, 15 C57Bl/6 and 15 IFNgR KO mice were given three exposures to 500 attenuated cercariae, and then terminally bled 14 days after the last exposure to provide serum for a passive transfer experiment (MT1). The ability of the serum to confer protection on groups of five naïve recipient C57Bl/6 mice was tested by administration of 400 µl of immune serum, or control serum from naïve mice, *via* the tail vein on two days after percutaneous challenge *via* the shaved abdomen, with 200 normal cercariae. Worm burden was determined as above, 5 weeks after challenge, and % protection conferred by administration of immune relative to naïve serum was calculated. Two further passive transfer experiments, MT2 and MT3, were then performed with serum collected from groups of IFNgR KO mice only, 2 weeks after the third vaccination (the G sera). Days for administration of donor serum to naïve recipients were chosen to pinpoint which larval stage was vulnerable to antibody-mediated elimination mechanisms.

### Array Design

**Figure 1:**
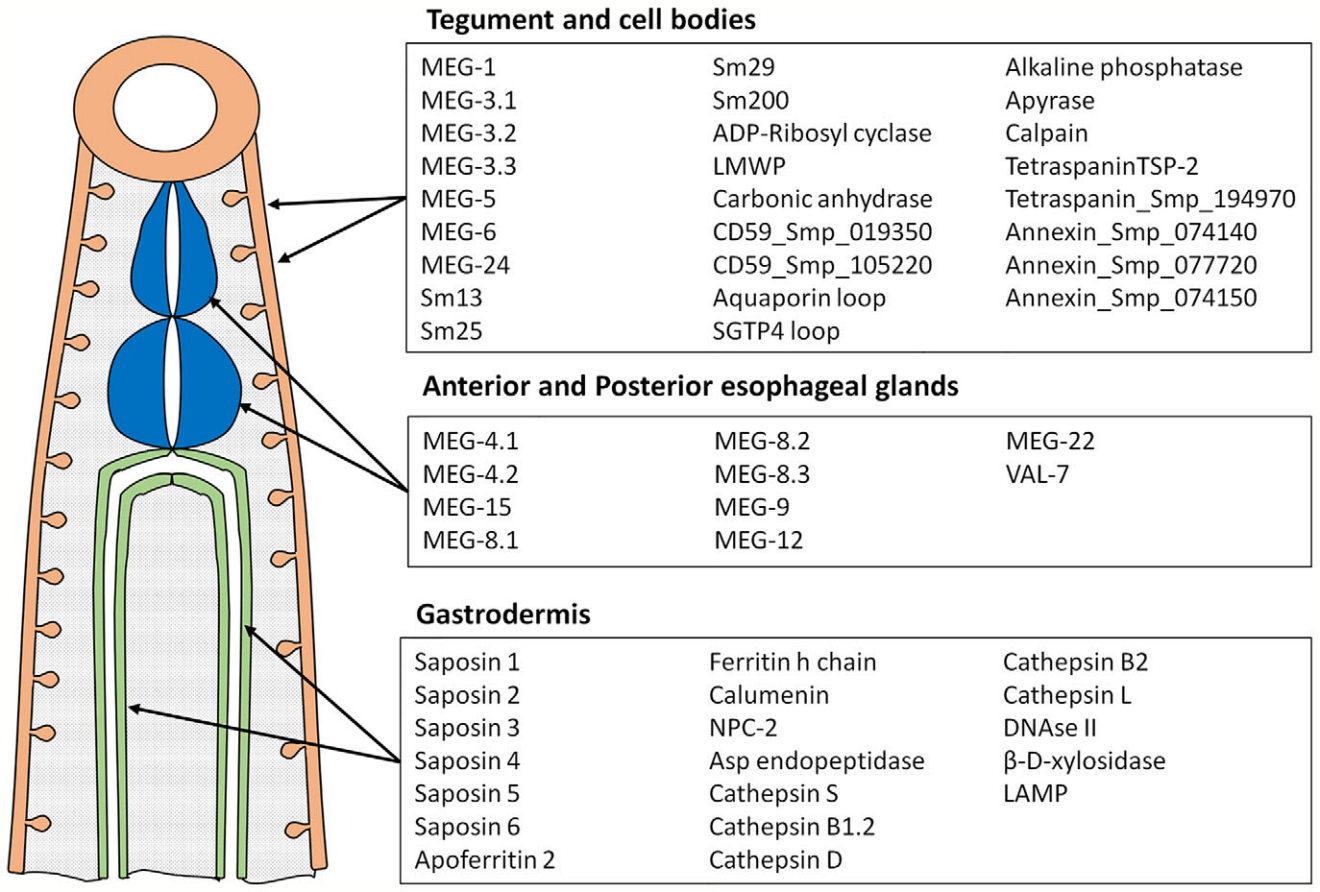
Diagram showing tissues where proteins printed on the array originate and the list of proteins in each category. These tissues represent the major parasite-host interface where antigens are exposed or released into the blood stream.

Peptide arrays were designed in consultation with PEPperPRINT (Heidelberg, Germany https://www.pepperprint.com/) and printed on glass slides using overlapping 15mer peptides, with a one, two or three amino acid offset, depending primarily on protein size. In total, we were able to print the sequences of 55 alimentary tract and tegument proteins, exposed at or secreted from the intra-mammalian stages, which comprise the major host-parasite interface. Proteins for inclusion on the four arrays were selected on the basis of our own studies of the worm transcriptome and proteome, reinforced by whole mount in-situ hybridization and immuno-localization to confirm tissue of origin (20, 22, 39–49). In selecting targets we took a conservative view that secretion, or location on the external surface of a membrane would be indicated by a signal sequence, or in a small number of cases (primarily the annexins) there was evidence from other cell systems for an alternative pathway of cell egress. The signal peptide was excised since it would not appear in the mature protein. Regions predicted to be heavily O-glycosylated were also omitted, the logic being that densely decorated peptide backbones would not be directly accessible to immunoglobulins in an infected host. The longest isoform of MEG proteins was printed; while exon skipping in these proteins can generate amino acid sequences that might be neo-epitopes, cost precludes printing all possible combinations. The cellular origin and list of proteins selected for inclusion is shown in **Figure 1**; the sequences printed and their full Smp designations are given in **Supplementary Figure 1**. Array 1 comprised 2329 unique peptides from 15 short alimentary tract proteins. For MEG-4.1, the known heavily glycosylated repeat central region (40) was omitted. Array 2 comprised 2,089 peptides from 14 longer alimentary tract proteins, with the predicted central glycosylated region of LAMP-1 omitted. Array 3 comprised 1914 peptides from 20 short tegument surface proteins and Array 4, 1,913 peptides from six longer tegument surface proteins. Only the external loops of the two tetraspanins, aquaporin and SGTP4 were printed. All proteins were printed in duplicate as overlapping 15mers. Quality control peptides, polio marker (KEVPALTAVETGAT) and HA tag (YPYDVPDYAG) were printed around the periphery. Each slide was printed with two copies of an array for incubation in a 3 x 2 well incubation tray. The arrangement of peptides on each of the four arrays can be seen from the row and column coordinates on the four pages of the spreadsheet in **Supplementary Table 1**.

### Array Screening

Peptide Microarrays were screened exactly as described in Li et al (35); see **Supplementary Figure 2** for a flow chart. Mouse primary antibodies were applied at a 1:200 dilution throughout. Their binding was detected using Cy3 labeled Goat anti-mouse IgG (H+L), at 1:300 dilution (A10521, Thermo Fisher (Cramlington, Newcastle, UK). The two quality control antibodies provided by PEPperPRINT, pre-labeled with Cy3 (Polio; KEVPALTAVETGAT) and Cy5 (HA; YPYDVPDYAG), were used at 1:1000 dilution. Two arrays per slide were each treated with 400µl (300µl for PEPperPRINT controls) of the appropriate solutions per well, with slow orbital shaking. Blocking, secondary antibody and control antibody solutions were each incubated at room temperature for 30 min; primary antibody solutions were incubated overnight at 4°C. Arrays were scanned using an Agilent DNA Micro Array scanner with High-Resolution SureScan Technology (Agilent Technologies LDA UK Limited, Stockport, Cheshire ; model G2565CA). The instrument has a dynamic range > four orders of magnitude; by optimizing antibody dilutions the arrays were never saturated while weaker reactivities were still captured. A screengrab of the Agilent image was taken for orientation and editing purposes.

### Data Analysis

The Agilent.tif file output for each array was analyzed using the PepSlide^®^ Analyzer (PSA) software as previously described (35). Heatmaps were then made from the cell scores for each array to facilitate visual interpretations. As the same Cy3-Rabbit anti-mouse detection reagent was used throughout, the mean PSA scores for each position on the array allow comparisons of the intensity of reactive regions between individual samples. PSA scores were color-coded on a linear scale using the Conditional Formatting function in Excel to highlight reactive regions and facilitate comparisons between samples. The within-group and all-group means for each 15mer peptide were calculated. An aggregate score for each reactive region was determined by summing adjacent peptide means above a predetermined threshold, down the array. In turn these aggregates were combined to give a reactivity score for each of the 55 proteins under investigation.

### Mapping Reactive Regions to 3D Protein Structures

A total of 26 crystal structures with sufficient sequence homology to array proteins was identified in the RCSB Protein Data Bank (https://www.rcsb.org/). To provide additional criteria for selection as vaccine candidates, we mapped the reactive regions of each protein onto the 3D crystal structure using CCP4mg software (50; http://www.ccp4.ac.uk/MG/). It should be noted that four of these were *S. mansoni* structures and the remaining 22 were the nearest homologues on PDB. A degree of homology between each array peptide and its protein subject was required for successful mapping to a crystal structure; the few instances where an antigenic peptide failed to map are indicated in the summary table (**Supplementary Table 3**). Short regions of reactivity could be mapped directly, while longer regions were arbitrarily segregated into short runs of ~ nine amino acids, the number being derived from our previous work mapping antibodies raised against 15mer synthetic peptides to predict epitope length (40). Where present, inhibitors/substrates were displayed in the active site of enzymes, with atoms colored using a modified CPK (Corey/Pauling/Koltan) scale, to indicate the location, while the side chains of amino acids comprising the active site were highlighted as cylinders. Regions of reactivity identified by the array screen were displayed on the crystal structure as “worms”, which were colored by solvent accessibility from blue (buried residue) to red (accessible residue). This parameter provides a calculated estimate of amino acid residue accessibility, indicating its likely exposure for antibody binding in the correctly folded native protein. No MEG protein structures have been solved but for members of MEG 3, 4 and 8 families, conserved regions were aligned using Clustal Omega (https://www.ebi.ac.uk/Tools/msa/clustalo/) on the assumption that these represented points of interaction with host macromolecules, which might make suitable antibody targets. The reactive regions of these eight MEGs on the array were then mapped to their respective aligned sequences.

## Results

### Serum From IFNgR KO Mice Confers Passive Protection Against Migrating Schistosomula

**Figure 2:**
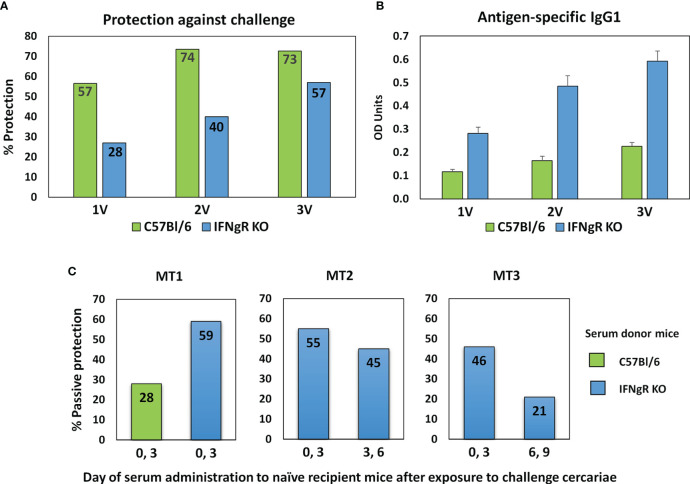
Properties of sera from IFNgR KO mouse used to screen the arrays. **(A)** Percent protection of C57Bl/6 versus IFNgR KO mice given one, two or three vaccinations with 500 radiation-attenuated cercariae and then challenged 5 weeks later with 200 normal cercariae. **(B)** Antigen specific IgG1 antibody levels in sera recovered from these mice 14 days after each vaccination. **(C)** Passive protection against a 200 cercarial percutaneous challenge of naïve C57Bl/6 recipients given injections of serum recovered from 3x vaccinated C57Bl/6 or IFNgR KO mice on the designated days. MT2 and MT3 sera were used to screen the four peptide arrays.

The protective immunity and associated specific IgG in C57Bl/6 and IFNgR KO mice induced by one, two or three exposures to attenuated cercariae, showed markedly different profiles (**Figure 2A**). For C57Bl/6 mice a single vaccination induced 56.5% protection, increasing to 73.5% after two exposures with no further rise after three (72.6%). In contrast, the rise in protection in IFNgR KO mice was almost linear with the number of exposures (28 to 40 to 57%), but not to such a high maximum. In both groups of mice schistosome-specific IgG1 was the major isotype produced, with only low levels of IgG2a (data not shown) (**Figure 2B**). However, at all sampling times the anti-schistosome titer in the IFNgR KO mice was about 2.5 times higher than in C57Bl/6 mice. The potency of the 3x vaccine sera from the two mouse groups was compared directly by administration to naïve recipients at Days 0 and 3 after cercarial challenge. The C57Bl/6 donors conferred 28% passive protection versus 59% for the IFNgR KO donors, corresponding to the differential antibody titers (**Figure 2C**, MT1). In the two subsequent passive transfer experiments with IFNgR KO mouse donors only (**Figure 2C**; MT2 and MT3), the serum administered at Days 0 and 3 conferred 54.9% and 45.7% protection against cercarial challenge, respectively. The same sera administered on days 3 and 6 gave reduced protection of 44.5% (MT2) and when administered on days 6 and 9, only 21% (MT3). We interpret these data to mean that the targets of the antibody-mediated passive protection are the early stage schistosomula undergoing migration through the lungs.

### Proteins From the Alimentary Tract Are More Reactive Than Those From the Tegument

**Figure 3:**
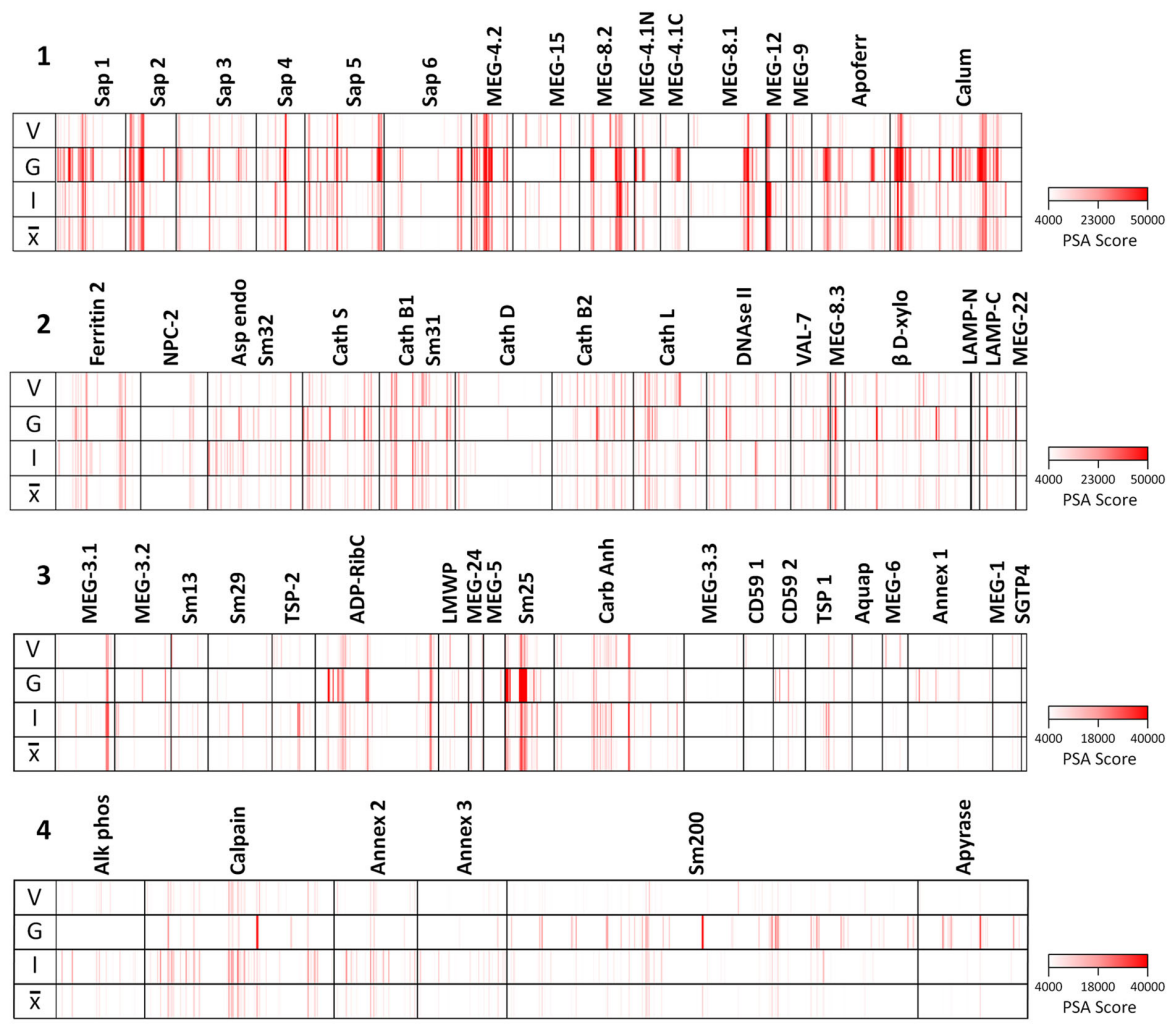
Heatmaps of the mean reactivities of murine serum pools against the peptide arrays: 1) short alimentary tract; 2) longer alimentary tract; 3) short tegument surface; 4) long tegument surface. V, mean of three C57Bl/6 samples from mice vaccinated with three exposures of 500 radiation-attenuated cercariae. G, mean of two IFNgR KO samples, MT2 and MT3 passive transfer experiments. I, mean of three C57Bl/6 samples from mice exposed to 500 normal cercariae for 4 weeks. $\bar{x}$, mean of all eight pools.

The full dataset obtained from screening the four arrays using the various sera is available in **Supplementary Table 1** and presented in **Supplementary Figure 3** as a series of color coded heatmaps to indicate the intensity of IgG binding against each 15mer peptide. In the text, the group means are illustrated to simplify description of the overall patterns of reactivity (**Figure 3**). The highest array cell score was ~40,000 units for the tegument arrays (Sm25) and ~50,000 for the alimentary tract (calumenin) (**Supplementary Table 1**). The number of neighboring reactive 15mer peptides ranged up to 20 for a few proteins (e.g. MEG-4.2, MEG-8.2) and even 25 for calumenin, but was mostly smaller, in many cases likely representing a single epitope (**Supplementary Table 1**). Inspection of the heatmaps (**Figure 3**) reveals considerable variation in the intensity of reactivity of the three different groups of sera, between the four arrays. Overall, Array 1 comprising short gastrodermal carrier proteins and esophageal secreted MEGs, shows the strongest reactivity while Array 2, comprising largely gastrodermal enzymes, is less intense. With a few exceptions, the short tegumental proteins on Array 3 are even less reactive (note PSA score), and the long tegumental proteins on Array 4 show the weakest response of all. Unsurprisingly, the high titre sera obtained from 3x vaccinated IFNgR KO mice (G) show markedly stronger reactivity than those from the similarly exposed C57Bl/6 mice (V), especially on arrays 1, 3 and 4. A surprising feature of the reactivity is that although we used genetically inbred mice, there is significant within-group variation in response to array peptides. The short alimentary tract protein MEG-8.2 provides an example, with a broad region of reactivity recognized by all eight sera (**Supplementary Table 1**, row 40, col 44-61) versus a neighboring set of peptides that strongly binds only infection serum 1 (**Supplementary Table 1**, row 42 col 7-11).

### Esophageal MEGs and Intestinal Transporters Show the Greatest Reactivity With IFNgR KO Sera

**Figure 4:**
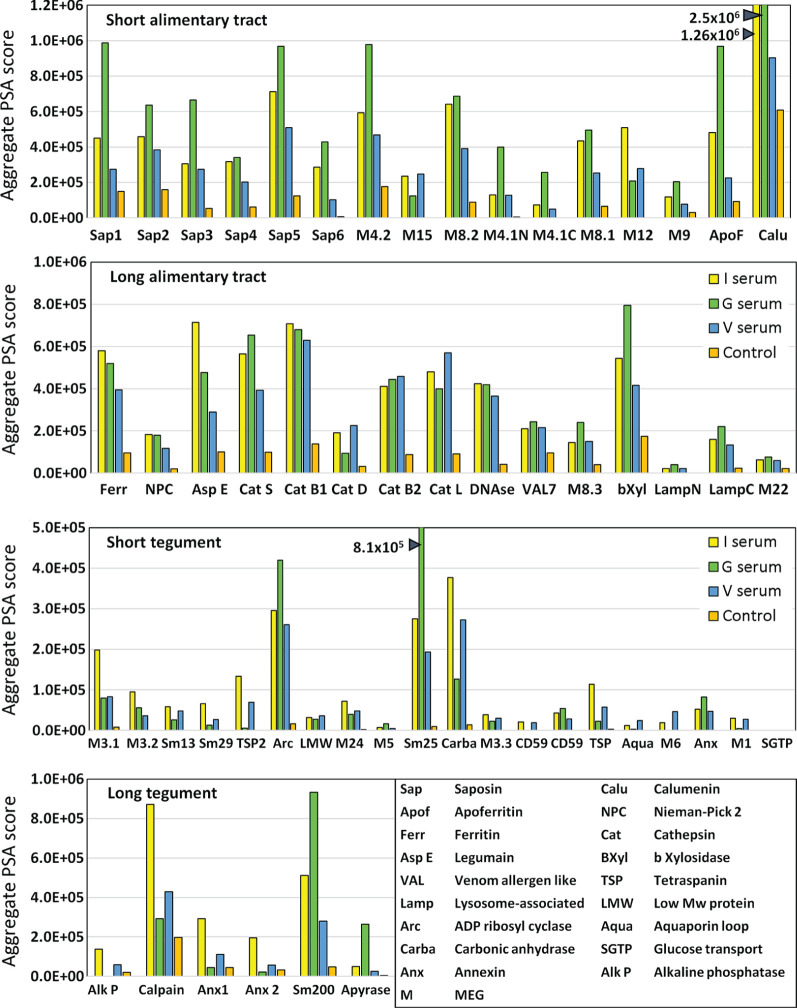
Bar chart summarizing the reactivity of all proteins on the four arrays, based on the data in **Supplementary Table 2**. The y axis is cumulative Agilent peptide score above zero, ignoring protein length for each protein. The means of three I, three V, and two G results are plotted plus one control serum. Note the much weaker response of tegument compared to alimentary tract proteins (y axis range).

The complexity of data presented in the heatmaps was further reduced by summing the reactive regions within each protein (**Supplementary Table 2**) and plotting them in a bar chart (**Figure 4**). Viewed together, the heatmap (**Figure 3**) and bar chart (**Figure 4**) permit the proteins in each tissue of origin (**Figure 1**) to be graded by their overall reactivity. MEG-4.2 was the most reactive esophageal secretion followed by MEGs 8.1 and 8.2, all in rank order G>I>V (i.e. sera from Vaccinated IFNgR KO>Infected>Vaccinated mice). MEG-4.1 N and C termini, and MEG-8.3 were similarly detected most strongly by G sera. MEGs 12 and 15 reacted more strongly with I and V than G sera, while MEG-9 and VAL-7 were the weakest reactors. However, these last four proteins reacted primarily at a single region; for MEG-12 this was at the extreme N terminus and for VAL-7, the C terminus. MEG-22 also has a single N-terminal reactive region, most notable in the G sera.

The transporter proteins secreted from the gastrodermis were also strongly reactive, especially the putative calcium transporter calumenin, followed by apoferritin, saposins 1, and 5, in rank serum order G>I>V. The remaining saposins 2, 3, 4 and 6 were similarly preferentially reactive with the G sera, whilst ferritin 2 was most reactive against the I sera. The one exception among the transporters was the Nieman-Pick 2 (NPC2) cholesterol transporter, only weakly detected by the three groups. It is notable that the sentinel mice also showed a moderate reaction to calumenin (**Figure 4**), potentially implying a pre-sensitization by some other agent. The gastrodermal secreted proteases, cathepsin B1, B2, S and L were all reactive over multiple regions of their sequences at roughly equal strength for G, V and I sera, but asparaginyl endopeptidase was most reactive with the I sera. In contrast, cathepsin D was immunologically unreactive with all sera. The glycosyl hydrolase, beta xylosidase and the DNAse were moderately reactive against all sera, the former most so with the G sera. Lastly, the C terminus of lysosomal associated membrane protein, LAMP-1, was weakly reactive with all groups.

### Few Tegument Proteins Show Marked Reactivity

The tegument proteins are harder to segregate into functional groups as many constituents have only been tentatively assigned to the plasma membrane or its overlying membranocalyx (43). The most prominent reactive protein, especially with G sera, was Sm25 of unknown location and function. Among potential membranocalyx constituents Sm200, Sm29, Sm13, and TSP-2, only Sm200 was reactive, particularly with G sera, while the others were weakly detected with I sera. The reactivity of GPI-anchored tegument enzymes, ADP-ribosyl cyclase and carbonic anhydrase, on the outer leaflet of the plasma membrane, were notable, greatest for G sera in the former, and for I and V sera in the latter. In contrast, GPI-anchored alkaline phosphatase and CD59 Smp_015390 were virtually silent; only CD59 Smp_105220 showed weak reactivity. Membrane-spanning apyrase (ATP-diphosphohydrolase), with a large extracellular domain, reacted only with G sera, while the external loops of the membrane transporters aquaporin and SGTP4 were virtually silent. The tegumental enzyme calpain showed moderate reactivity, especially with I sera. The three annexins and tetraspanin Smp_194970 loop likely play a membrane structural role, and all were weakly reactive with I sera. There are four tegument-associated MEGs, but only MEG-24 showed weak reactivity with all three sera. Finally, the three members of the MEG-3 family, expressed in the schistosomular tegument and head gland, reacted principally with I sera, in rank order MEG-3.1>3,2>3.3.

### Normalizing by Amino Acid Content Allows Ranking of Protein Reactivity by Size

**Figure 5:**
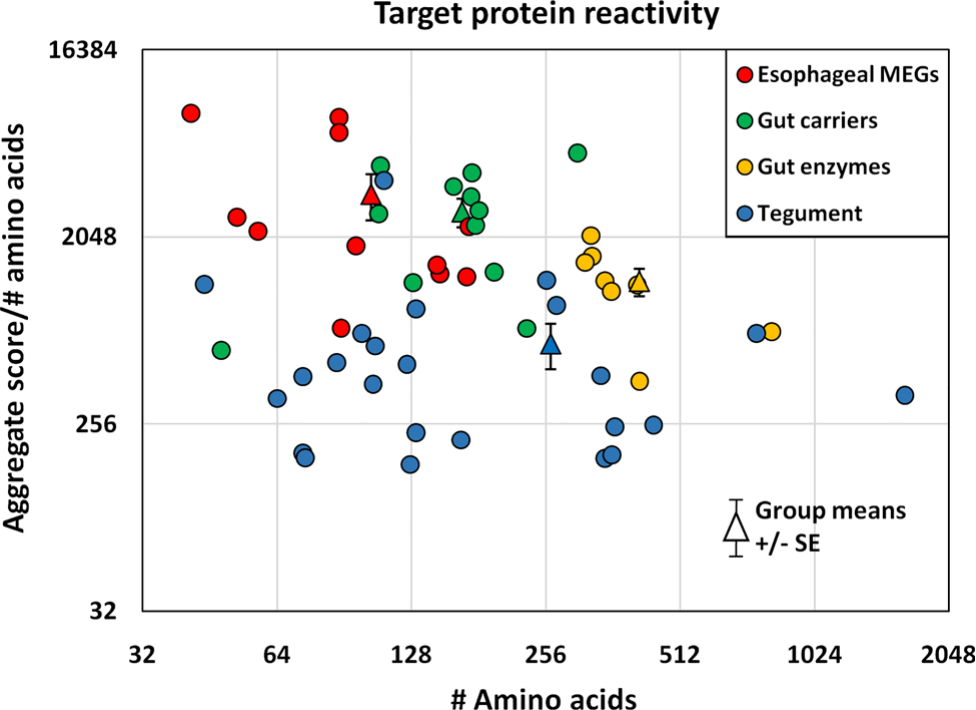
The mean reactivity of array proteins based on the data in **Supplementary Table 2**, normalized for number of amino acids and segregated into four groups by tissue of origin. The differences in group reactivity were tested for significance using a t-test. Note the log y axis scale.

The proteins printed on the four arrays differ in size from 8 to 200 kDa, and the divergence was normalized to create an index by dividing the mean reactivity score (**Supplementary Table 2**) by the total number of amino acids in the printed protein. The indexes, displayed as a scatter plot (**Figure 5**), and sorted by tissue of origin, segregated into four distinct but overlapping groups. Based on amino acid content (AA) the esophageal MEGs + VAL-7 are the smallest alimentary tract proteins ($\bar{x}$ 100 AA), followed by gut carriers + LAMP ($\bar{x}$ 166 AA) and finally hydrolases ($\bar{x}$ 414 AA); the 17 tegument proteins range between 44 and 1630 AAs ($\bar{x}$ 262 AA, skewed by Sm200; median = 129 AA). The mean reactivity values for each functional group show the esophageal MEGs lying at the top left ($\bar{x}$ 3285 +/- S.E. 827), the gastrodermal carriers in the center ($\bar{x}$ 2689 +/- S.E. 427), and the gastrodermal enzymes to the right ($\bar{x}$ 1249 +/- S.E. 187). All means are significantly greater than the tegument proteins ($\bar{x}$ 625 +/- S.E. 155; P<0.001 for MEGs and carriers, P<0.05 for enzymes), distributed along the bottom of the plot; the carriers are significantly different from the enzymes (P<0.05) but not the MEGs. Taking the tegument proteins as unity, the esophageal MEGs are 5.25x, the carriers 4.31x and the gastrodermal enzymes 2.0x more reactive with the murine IgG samples.

### Mapping the Reactive Regions to Protein Structures Reveals the Location of IgG Binding Sites

**Figure 6:**
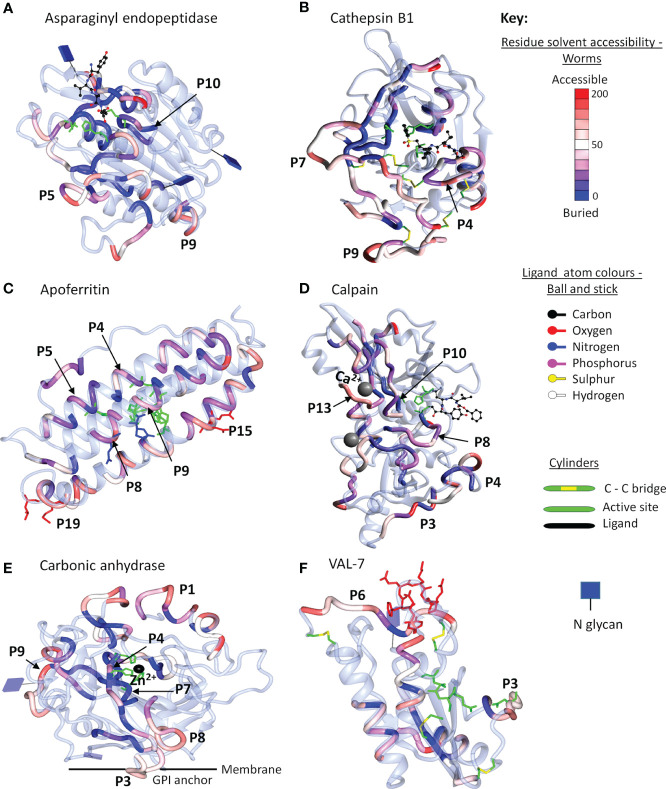
Reactive regions mapped onto six selected crystal structures using CCP4mg (50). **(A)** Aspariginyl endopeptidase. **(B)** Cathepsin B1. **(C)** Apoferritin. **(D)** Calpain. **(E)** Carbonic anhydrase. **(F)** VAL-7. The protein chain is colored ice-blue in ribbon format. The position of each reactive region listed in **Supplementary Table 3** is shown in worm format and selected key peptides from **Table 1** or **Supplementary Table 3** are indicated by P number. The worms are colored by solvent accessibility ranging from buried (blue) to accessible (red) on a scale 0-200 Å^2^, based on the algorithm described by Lee and Richards (51); the detailed key for each model is illustrated in **Supplementary Figure 4**. Where present, inhibitors/substrates are displayed in the active site of enzymes in ball and stick format, with atoms colored using a modified Corey/Pauling/Koltan (CPK) convention, to indicate their location, while the side chains of amino acids comprising the active site or other named features are highlighted as cylinders.

The final step in the mapping process was to use 3D crystal models of the native molecule to identify which regions in the most reactive proteins were likely to be accessible to antibodies, and hence were likely to have the potential for neutralization of function and/or formation of insoluble immune complexes. We elected to map the strongest reactive region in each instance, rather than mean values across the array, so that all potential epitopes were covered, even if detected by only one serum sample. To avoid repetition in the text, the full data set of antigenic peptides, extracted from **Supplementary Table 1**, is tabulated by individual protein as **Supplementary Table 3**, which also gives the number of sera in which a reactive region was found, the aggregate PSA score of the region, and pertinent comments on crystal location. Six representative structures are illustrated in **Figure 6** and the full set of 26 mapped proteins is presented in **Supplementary Figure 4** with a color key and additional commentary where necessary. The two best peptides selected for all antigenic proteins, mapped or otherwise, are presented in **Table 1**.

**Table 1:** Principal Reactive Epitopes.

Protein	*	Peptide 1	Why chosen	Peptide 2	Why chosen
Cathepsin B1.2	G	P4 ESCGLGCEGGILGPA	active site	P10 KAQKEIMKYGPVEAS	loop
Cathepsin B2	G	P12 TTNGCQPYEFPPCEHH	occluding loop	P15 NGPVEVDFEVYADFP	beta sheet-loop
Cathepsin L	G	P7 ELSNDPLPSKWDWRD	junction propep	P12 SSCHFRKSKGVVKVK	loop
Cathepsin S	G	P10 DHECVENPVSVAFDF	active site	P13 SGVLLIDDCQNEEPF	loop
Asp endopep	G	P7 TDHGAPGLIAFPDDE	loop	P13 VSEFQGSRDKSSTEN	α cleavage site
Apoferritin	G	P4 QILNEYEAFYIYDHM	ferroxidase	P15 NDATTQDFLDDFLQE	Fe entry channel
Ferritin 2	G	P10 EFFRKASHEEREHAE	ferroxidase	P17 LTDFIESQYLHEQED	Fe entry channel
Saposin 1	G	P5 GRLIKIFLEDEPFIP	hinge 1	P8 CKLIPERHWRNECLD	loop
Saposin 2	G	P4 SLAHYFMDDLYWRDV	hinge 1	P8 EVGSLIGPGICTDFD	hinge 2-helix 4
Saposin 3	G	P3 ICSLTGSFEVQCSFL	loop	P6 MFIDKYIDTISTMDP	hinge 2
Saposin 4	G	P2 WQTYLNSTSVDEKIE	hinge 1	P5 YFFFRYRCEEFMERY	loop-helix 3
Saposin 5	G	P3 RGIRWLLLQSYTRKT	hinge 1	P8 HDFVVNMSVYEPCQY	hinge 2-helix 4
Saposin 6	G	P3 GMNLVHYILSEDYWV	helix 1	P4 HYILSEDYWVKIYMI	hinge 1
Nieman-Pick 2	G	P2 VPIPFALDNSNLCEF	apolar rim	P4 WELQDSSNEDIVCVE	loop
LAMP-1C	G	P2 LSNATINDNESVCSE	loop-beta strand	P5 IPVGSYYSCLTVPEI	loop-beta strand
Calumenin	G	P1 LETVSRRDTEHFADD	v immunogenic	P17 STPFDENEPEPEWVE	v immunogenic
b Xylosidase	G	P4 LSFEADVPEHDLWMT	v immunogenic	P12 IGMWGTDIDEGYWWI	v immunogenic
DNAse	G	P4 KWTKPLGYDRENLWW	v immunogenic	P7 LNHFSKSSDFGKDLY	v immunogenic
Apyrase	T	P2 RLKLIEDPLGSLDLF	loop	P7 LQGYKSDLYNAFEWF	short helix
ADP Ribosyl cyc	T	P1 NISCSEIWNSFESIL	helix	P2 KSACVMKSGLFDDFV	loop
Carb anhydrase	T	P3 TWFISFDGILDYKYE	loop	P9 MHQIVESIKYEQTAF	loop
Calpain	T	P3 TERTLWEDPDFPAND	loop	P13 LSFTADGEFWMSYED	Ca2^+^ binding
TSP-2	T	P5 QQKLHCCGADSPKDY	loop	P6 KDYGENPPTSCSKDG	loop
TSP-194970	T	P3 QYYEKHPNYENQVDN	loop-helix	P4 PNYENQVDNLQREFF	helix
Annexin 074150	T	P1 RSLIHSFDPHGKHYR	loop	P9 KSLLNAVKDDTSGDY	helix-Ca binding
Annexin 074140	T	P3 MSQVEQDVEILWDAG	lipid binding	P5 EDWIRNETSGDYQRL	loop-Ca binding
Annexin 077720	T	P4 LESDIIKETKPPYEQ	loop-Ca binding	P8 LYDSMYGLGTREDTL	loop-Ca binding
CD59 105220	T	P3 IAKDCVASCVPQDRR	loop helix	P4 GGKAGLVTECCDEDY	loop beta strand
Sm13	T	P1 EPEPEPEPVPVSRNS	v immunogenic	none	
Sm25	T	P1 NSNSIITDEDYDHYN	v immunogenic	P2 FHRNSDPDGFPEYEF	v immunogenic
Sm200	T	P13 CRPTYLLLEFIEPNI	v immunogenic	P16 PGRRYELQAEVIYTE	v immunogenic
VAL-7	E	P3 CVPRRSNMTMRKGSK	loop-Cap site	P6 KYANRQPYDPIYPED	loop
MEG-4.1	E	P1 PLDDRFNDVNTIN	N terminus	P2 PLYYMVEKFVQIMGY	conserved C-term
MEG-4.2	E	P4 NHNKFHEMPEYDDQL	v immunogenic	P8 PLWLVNPIYYVLELF	conserved C-term
MEG-8.1	E	P7 GDGFFDLFSEQEFHP	imm & conserv	P9 KSYLFNFWYLFRTSF	conserved
MEG-8.2	E	P7 KFNSIFGEEEYNPPK	imm & conserv	P8 EYNPPKDSDFTERLW	imm & conserv
MEG-8.3	E	P2 FNPPKESEFYERFWE	conserved	P3 FYERFWELFKHCFLN	conserved
MEG-9	E	P2 SSTEGQNHEESQFFL	immunogenic	P4 AHFLQFLNGCFLNMD	immunogenic
MEG-12	E	P1 ENYEQQLQQP	v immunogenic	P2 QQLQQPKAYGIWSLF	v immunogenic
MEG-15	E	P2 LSHHNTVPAKTTRKS	immunogenic	P5 IPQIKTIEFSQNENL	immunogenic
MEG-22	E	P1 GSPVPHPSVYFDNPE	N terminus	none	
MEG-3.1	S	P1 AERECKKHCEGNNEY	conserved	P6 GSFPLCLYNCDQEHP	conserved
MEG-3.2	S	P1 TQQECVRHCGGHNEY	conserved	P5 LYNCDQGNGSGNFDE	conserved
MEG-3.3	S	P1 QQECEKNCKGDNEYV	conserved	P2 TDPRKDGADGSEDFD	conserved

*Tissue of origin: G, Gastrodermis; T, Tegument; E, Esophageal glands; S, Lung schistosomulum.

Pairs of peptides selected from the 44 reactive proteins detected on the arrays, based on their aggregate score, and accessibility indicated by mapping to crystal structures.

Amino acids in red are predicted epitopes.

#### Gastrodermal Enzymes

For enzymes, the ideal antigenic peptide would be accessible, include an amino acid involved in substrate binding, and have a high aggregate PSA score. When the reactive regions of the gastrodermal secreted cathepsins B1.2, B2, L and S plus asparaginyl endopeptidase (**Supplementary Table 3**) were all successfully mapped, these conditions were rarely fulfilled. The antigenic regions lay in external loops and α helixes, while the color gradations on the models indicate that some epitopes were buried and unlikely to be accessible to IgG in the native folded state. The mapping to the crystal structure of asparaginyl endopeptidase (**Figure 6A**) provides a worked example. The color-coded reactivity of all eight sera plus the control, against the sequential 15mer peptides of the enzyme, with a two amino acid offset, are shown in **Supplementary Table 3**, page 1. Moving down the array, the most reactive region is copied over to the Aggregate column and its aggregate PSA score calculated. The 15mer peptide that best represents the center of the region is then selected, and the core 8-10 amino acids highlighted for mapping to the crystal structure. For asparaginyl endopeptidase there are 16 distinct regions, but P1 is not in the crystal structure. When the remainder are mapped onto the crystal, the color range runs from accessible red to buried blue. Thus, P10 contains the cysteine that forms part of the active site but has the lowest aggregate score (26107), and a blue/purple color so is unlikely to be accessible to antibody (**Figure 6A**). P5 (164692) and P9 (170611) in external loops had the highest PSA scores but present a dilemma; both are reactive only with infection serum (pool I3). We therefore chose P7 (88288), reactive with both G sera, so potentially involved in protection. A second possibility presented itself in the mapping of the asparaginyl endopeptidase regions to a different crystal model containing the pre-protein (**Supplementary Figure 4.8**). Here, P13 (78797) mapped to the alpha cleavage site so binding of antibody there could potentially interfere with protein activation. The downside is that this region was only weakly reactive with the two G sera.

Similar considerations were applied to the other gastrodermal proteases. P10 of cathepsin S encompassing the S2 substrate pocket, detected by almost all sera, had the lowest score out of 15 peptides (**Supplementary Table 3**). The next best situation would be a region where antibody binding in proximity, might block the active site. P4 of cathepsin B1.2 is the best example in an accessible loop close to the site, with a good PSA score (**Figure 6B**). Interference with activation is also feasible for other proteases. Thus, the junction with the propeptide in cathepsin L at P7, was antigenic and accessible. Finally, P12 encompassing the occluding loop of cathepsin B2, which regulates active site function, was strongly antigenic and surface accessible. The remaining peptides selected for the above five proteases were loop structures chosen for their accessibility and high PSA score.

#### Gastrodermal Carrier Proteins

Apart from calumenin, all the gastrodermal secreted ferritins, saposins, and NPC2 were mapped to crystal structures (**Supplementary Figure 4**). The nanocage of ferritins, comprising 24 units each composed of four α-helix bundles, has a complex structure with eight entry channels admitting Fe^2+^ ions to 24 ferroxidases *via* transit sites for conversion to the stable Fe^3+^ state (see **Supplementary Figure 4.9**). Apoferritin is strongly antigenic along segments of the helixes; Peptides 4, 5, and 9 encompass the ferroxidase and P8 the transit site, but many of the relevant amino acids are on the internal surface of the helixes, so potentially inaccessible in the intact cage (**Figure 6C**). The most obvious targets on apoferritin are the P4 ferroxidase and P15 entry channels, with high PSA scores, where antibody binding might disrupt function (**Supplementary Table 3**). The situation is similar with Ferritin 2, where P10 encompasses the ferroxidase site and P17 an entry channel (**Supplementary Table 3**). The schistosome saposins possess a domain (two in Sap 6) comprising four α helixes separated by two hinges and a loop. They combine in pairs, unfolding to create a lipid-transporting disc (**Supplementary Figure 4.12**). Reactive regions were located in the helixes of all six mapped structures but the hinges and loop appeared the most accessible, with a high PSA score. For saposins 1-5 it was possible to select a hinge-loop combination of peptides and for saposin 6, hinge 1 and helix 1 peptides. NPC-2 binds cholesterol in a deep hydrophobic pocket sandwiched between the two β-sheets, its entry being regulated by an apolar rim (**Supplementary Figure 4.19**). The protein was poorly reactive with all sera, but P2 with the lowest score, encompasses two amino acids in the apolar rim, while P4 is an accessible loop (**Supplementary Table 3**). Lastly in this group, the LAMP-1 protein mapped only poorly to the available crystal structure (**Supplementary Figure 4.30**); the P2 and P5 surface loop/β strands of the C-terminal β-prism fold structure were selected as targets.

#### Tegumental Proteins

Four tegumental enzymes were mapped, calpain with 22 regions being the most reactive, although only half were in the crystal model. Residues in the active site were not reactive, so high scoring accessible loops P3 and P13, the latter comprising one of the two Ca^2+^ binding sites involved in activation, were selected (**Figure 6D**). For carbonic anhydrase (a *S. mansoni* crystal), two histidines of the catalytic site and the Zn^2+^ binding location were present within P4 and P7 but both regions were buried (blue) within the hollow core of the protein (**Figure 6E**). Therefore, two surface loops, P3 and P9, were selected, being detected by most sera (**Supplementary Table 3**). ADP ribosyl cyclase presents a similar dilemma since it functions as a homodimer on the membrane with the two catalytic sites apposed, so although P6 with active site glutamine residue and P7 substrate interaction site were reactive (but low PSA score), they may not be accessible *in situ*. Two loops comprising P1 and P2, recognized by all sera and distal to the membrane anchor, were therefore selected (**Supplementary Table 3**; **Supplementary Figure 4.21**). The external domain of apyrase was detected predominantly only by G sera. The catalytic site was immunologically silent so the P2 loop and P7 short helix were selected, based on PSA score (**Supplementary Table 3**).

The two tetraspanins and three annexins, all relatively unreactive especially with G sera, were mapped to crystals. For TSP-2 (another *S. mansoni* crystal), the high-scoring P6 loop and the partially buried P5 loop were selected, while for TSP Smp_194970 P3 and P4 were selected, even though they were partially or completely helical structures, respectively. The situation with the three annexins is more complex; they contain multiple binding sites for Ca^2+^ ions on the convex surface, involved in interaction with membrane phospholipids. For annexin Smp_074150 (another *S. mansoni* crystal structure), all PSA scores were low; P9 encompassed one of the six Ca^2+^ sites but this annexin is another example of a disulphide-linked apposed homodimer (**Supplementary Figure 4.26**) so the peptide may not be accessible *in situ*. The exposed P1 loop was chosen as the second target. Annexin Smp_074140 with few reactive regions, lacks the dimerizing cysteine and the P5 Ca^2+^ binding site may be accessible. In addition, although P3 is part of a helix, it incorporates a membrane interaction site, so antibody binding could block function. Annexin Smp_077720 had more reactive regions, with helical P6 containing the membrane binding site but also the lowest PSA score of 10 peptides; the two calcium binding loops in P4 and P8 were therefore selected. The tegument surface GPI-anchored protein CD59, Smp_105220, was only weakly reactive with I sera; P3 and P4 were selected, part loop-helix and part loop-β strand, respectively, based on projected accessibility.

#### Esophageal Secreted Proteins

Among esophageal secreted proteins only VAL-7 could be mapped to the crystal structure of schistosome VAL-4 (**Figure 6F**). It was notable that both its caveolin motif and CAP domain were largely immunologically silent. However, all sera recognized P6, an exposed loop at the C-terminus, complemented by P3 adjacent to the CAP site. The esophageal MEGs lack homologous crystal structures but the conserved sequence of MEGs 4.1 and 4.2 (55% identical, 81% conserved amino acids in the C terminal domain) can be exploited to select peptides (**Supplementary Table 3** and **Supplementary Figure 5**). For MEG-4.1 the highly reactive peptides, N terminal P1 and C terminal P2 were selected, and for MEG-4.2 highly reactive P4 and reactive, conserved P8. A similar situation pertains with the three MEG-8 isoforms (18% identical and 52% conserved residues), with all three variants showing strong binding of sera in the conserved region **Supplementary Table 3** and **Supplementary Figure 5**), from where peptides were selected. The three members of the MEG-3 family, expressed in the schistosomular head gland and tegument (55% identical, 77% conserved residues) contain seven pairs of cysteines, separated by three amino acids, and spaced 9–12 amino acids apart, throughout each sequence, implying a rigid and highly organized structure (**Supplementary Figure 5**). With one exception at the C terminus of MEG-3.1, especially in I samples (**Supplementary Table 3**), the aggregate scores of reactive regions are weak. Nevertheless, aligned peptides can be selected in proximity to the N and C termini of all three proteins.

#### Unmapped Proteins

No structural mapping or sequence analysis proved possible for three gastrodermal secretions (β-xylosidase, DNAse, calumenin), four esophageal MEGs (9, 12, 15, 22) and four tegument surface proteins (Sm13, Sm25, Sm200, MEG-24). The only criteria for selecting peptides were their PSA score and extent of reactivity with the eight screening sera (**Table 1**). Sm25 was notable for being one of the most reactive proteins in the whole screen with very high PSA scores (**Figures 3**, **4**). Tegumental Sm13 and MEG-24 possess reactive and presumably accessible N-termini; Sm13 has only one reactive region (See **Table 1**). Esophageal MEG-12, one of the few known products of the of the anterior esophageal gland, has a similarly highly reactive N-terminus. The remaining proteins are tabulated without comment. As if to emphasize the low reactivity of tegument proteins, nine of the ten array subjects that failed to provide any antigenic information have an authenticated tegument surface location.

## Discussion

### Antibodies Mediate Protection After Vaccination of IFNgR KO Mice

A single exposure of C57Bl/6 mice to the RA schistosome vaccine elicits a strong Th1 response operating against challenge schistosomula in the lungs by blocking their onward migration and deflecting them into the alveoli (8, 9, 52). Further exposures to the vaccine increase the humoral component of protection (53) with the higher levels of antibody generated being able to confer protection passively on naïve recipients [(54) and the current data]. Conversely, IFNgR KO mice lacking the cell mediated arm of the immune response, are poorly protected by single exposure (36) but we show here that when such mice are given multiple exposures the level of protection increases in proportion to IgG1 titer. Pertinent to epitope mapping, these sera conferred a mean of 53% passive protection to naïve mice when administered on days 0 and 3 of challenge, almost double that of comparable C57Bl/6 mice. The reduction of protection at later times indicates either that the lung schistosomulum is a special immune target or that the act of pulmonary migration is particularly hazardous. The observations from the systems biology analysis (30) suggest that the subtle changes induced in hemostasis, coagulation and chemokine pathways by vaccination simply make the lungs a more difficult capillary bed to traverse.

### Antigenic Load in Infected Versus Vaccinated Mice

The inclusion of 28-day infection sera in the array screen was intended as a comparator to determine whether vaccine sera reacted with any unique targets. A potential limitation of this comparison is the disparity in antigenic load provided by ~150 pre-adult liver worms with an active gut versus 3 x 500 schistosomula that do not develop beyond the lung stage. The former are estimated to vomit 5.6 μg of alimentary tract proteins per day (Leandro Neves, personal communication) versus the total protein content of 500 lung schistosomula of ~ 20 μg (55) releasing ~0.2 μg of secretions per day (21). Whether such heavily infected mice would develop any protection cannot be assessed, due to the complications of egg-induced pathology and mortality that would follow patency around day 35. However, both the absence of protection after challenge of mice with a single sex infection (56) or chemotherapeutic cure before pathology develops (57) suggest they would not be protected.

### Selection of Proteins

We aimed to be comprehensive in our selection of proteins but there are some omissions from the target list, including a second tegumental calpain [46, Smp_137410, (58)] and phosphodiesterase 5 (59), based on their MW; the same criterion applies to the omission of α2 macroglobulin from the gastrodermis, with a MW ~230kDa. The esophageal glands are the most underrepresented, MEGs 11, 14, 31.1 and 31.2 being deliberately omitted as they are anchored in the surface lining and predicted to be heavily O-glycosylated. Cystatin, MEGs 3.4, 16, 17, and the MEG-26 family, plus two phospholipase A proteins, several aspartyl proteases and palmitoyl-thioesterase were also omitted; with the exception of cystatin their transcripts were of low abundance (41). Most recently several MEG-2 family members have been identified as differentially expressed in day 6 lung schistosomula (25), which may also be products of the head gland and tegument.

### Epitope Conformation

It is unclear whether the linear 15mer peptides on the arrays take up any native conformation such as an α-helix. However, the mapping of reactive regions on the array to crystal structures revealed antibody binding to α-helixes and β-sheets as well as extrinsic loops. The ability of the arrays to detect conformational epitopes, where key amino acids are brought together by protein folding into tertiary structures, is uncertain. Such structures are best understood in the context of the rigid capsids of viruses. At one extreme, the participating amino acid targets of two neutralizing monoclonals on the surface of human papilloma virus were widely dispersed throughout the target protein and even on an adjacent molecule (60). At the other, interaction of neutralizing antibodies with Hepatitis C virus E1 and E2 surface glycoproteins revealed epitopes the were largely linear, and the viruses evaded neutralizing antibodies by mutating amino acids within the epitope sites (61). The schistosome tegument is more fluid/flexible than a viral capsid but several surface proteins contain numerous disulphide bridges, implying a tightly folded 3D structure. The presence of conformational epitopes in Sm29 (18 Cs) the three MEG-3s (16 Cs) and two CD59s (10 Cs) could well explain their low reactivity with the mouse sera if epitopes were conformational rather than linear. This argument does not hold for cathepsin D with six cysteines, fewer than the other reactive cathepsins, or alkaline phosphatase with four cysteines, both of which are virtually silent.

### The Reactivity of Potential Targets

A more plausible explanation for the low reactivity of array targets, especially of the tegument proteins, is that they have been subject to evolutionary pressure by the immune system due to their exposed location (62). Indeed, it seems highly likely that a worm living for decades in the bloodstream would require an unreactive surface to survive and thrive. Adult worms fixed *in situ* in both rodents and primates show no evidence of adherent leucocytes (63) but immunoglobulins and complement factors have been identified by proteomics in tegument membrane preparations (20, 46). However, the membrane attack complex does not form so the immune evasion mechanisms deployed by established adult worms are adequate to ensure survival (52). It is possible that exposed tegument proteins possess conserved amino acids for interaction with external targets, having limited possibilities to mutate without loss of function. Epitope mapping may identify such regions if they are reactive. With the alimentary tract proteins, there is a different consideration. The gut lumen is an acidic hydrolytic environment akin the inside of a lysosome (39). It is unclear whether antibodies can survive in such a degradative environment to exert a blocking effect on gut function. The esophageal secretions may be in an intermediate situation, especially those of the anterior gland, since ingested blood is at pH 7.4 and must be rapidly acidified, either as it passes along the esophageal lumen or after entry into the gut lumen. Thus, there is a possibility that the esophageal secretions make more plausible immune targets.

### Selecting Vaccine Targets

When we evaluate the screened proteins for vaccine potential, normalized by size, the esophageal secretions are the most highly immunogenic, especially MEGs 4.1, 4.2, 8.1, and 8.2 from the posterior gland and the N terminus of MEG-12 from the anterior gland. It is notable that these same five esophageal proteins were reactive in the peptide array analysis of host responses to *S. japonicum* (35). Their proposed function is to initiate blood processing in the esophageal lumen (40, 41), and their reactivity is highest with the protective G sera. In addition, the six saposins and apoferritin from the gastrodermis have the strongest reactivity with G sera. Leaving aside the question of antibody stability, targeting the hinge/loop regions of saposins may be a good strategy to block lipid-binding. However, at least ten are encoded in the genome (49) possibly reflecting a degree of functional redundancy, as an evasion strategy. Conversely, there is only a single cholesterol transporting NPC2 protein, but it is a weak antigen. The gastrodermal proteases (Cathepsins B1.2, B2, S & L) present numerous targets but few epitopes lie near the active center so there appear to be limited opportunities for direct blocking of catalysis. There is also the potential for functional redundancy in the cascade. Prevention of activation by binding to a protease pre-protein is an alternative strategy, with asparaginyl endopeptidase as a desirable target since it also initiates activation of other peptidases (64). Ferritin 2 was included although it lacks a signal peptide, as it was identified by proteomic analysis of vomitus (39). Calumenin which was identified in the same study as a potential calcium transporter, has a signal peptide but is normally located within the lumen of the endoplasmic reticulum. On balance, the similarity of the schistosome gut lumen to the contents of a lysosome make these two proteins valid constituents of gut secretions, hence potential targets for a protective response. The screen of tegument proteins identified a smaller cohort of potential candidates. Calpain was highly reactive and has been much studied as a single-antigen vaccine (5). However, the vaccine potential of Sm25, ADP-ribosyl cyclase and carbonic anhydrase have not been reported. The reactive N-terminus of Sm13 also deserves attention due to the alternating proline and glutamic acid residues suggesting a novel function that might be blocked by antibodies.

### Esophageal Gland Secretions Are Plausible Targets

The reactivity of the sera from vaccinated mice with alimentary tract proteins has illuminated an unsuspected feature of schistosome physiology. Feeding on erythrocytes, and worm growth, only begins after arrival in the portal distributaries of the liver (44, 55) so it has been assumed that the larval gut was non-functional up to that point. However, electron micrographs of lung schistosomula *in vivo* show the gut lumen packed with granular material (10). The strong reactivity of the protective G group sera with esophageal MEGs and gastrodermal saposins indicates that both tissues are productive at the lung worm stage, although ingestion of erythrocytes is not occurring. This widens the scope of targets mediating elimination of challenge parasites in the lungs. Supporting evidence for the synthetic activity of the esophageal glands and gastrodermis has come from the recent publication of a transcript screen of the intra-mammalian stages (25). Scrutiny of transcript abundance in day 6 *ex vivo* lung worms revealed MEG-15 as the most abundant, while MEG-9, MEG-4.2, two saposins and two cathepsins were all in the top 30 most highly expressed transcripts out of ~10,600 genes interrogated (**Supplementary Figure 6**). In day 28 liver worms the intensity of expression of alimentary tract proteins was no longer quite so dominant but there were still eight representative genes in the top 100 transcripts (**Supplementary Figure 6**).

### Applications to Vaccine Development

How best might the information accumulated in this study be used to advance vaccine development? The data on protein reactivity lends itself to the development of monoclonal antibodies (mAbs) specific for individual epitopes on putative target proteins. This approach was fashionable 40 years ago but proved short-lived (see (65) for review). Peptide array screening allows us to pinpoint the reactive epitopes on individual target proteins; mAbs could then be generated against those epitopes using synthetic peptides as targets. They would be useful as reagents to test hypotheses generated in this study about blocking activity of antibodies e.g. of enzyme activation by binding a cleavage site; direct inactivation by binding in or near an active site; the prevention of lipid uptake by binding hinge and loop regions of saposins. The mAbs could also be administered in calibrated doses to infected mice, singly or in cocktails, to investigate *in vivo* effects. Although cost considerations undoubtedly preclude their development for use as a human schistosome control agent, it is worth noting that a cocktail of three monoclonal antibodies (REGN-EB3) has been successfully developed by Regeneron and licensed as a therapy against Ebola virus (66).

The alternative approach is to perform direct vaccination experiments, probably initially in the mouse model. Several array proteins have already been tested singly with varying degrees of success. They include TSP-2 (67), Sm29 (68), Sm200 (69), and calpain from the tegument, MEG-4.1 from the esophagus (70), and cathepsin B1.1 (71), Saposins 4 and 6 (72) from the gastrodermis. The mouse has limitations as a vaccine screen for schistosomes (7) but remains the most practical option for large scale testing. Individual proteins here highlighted could be tested singly (e.g. tegumental Sm25 or esophageal MEG-4.2). However, finding an Achilles heel in the form of a single magic bullet for a schistosome vaccine, whilst highly desirable, seems implausible in a complex and sophisticated blood-dwelling parasite. Therefore, we advocate an alternative approach whereby the coding regions of reactive epitopes are combined in a string-of-pearls synthetic gene. Expression of these genes as recombinant proteins in quantity would allow multiple proteins to be targeted simultaneously. As proof of principle we have already tested the approach in our analysis of the reactivity of *S. japonicum* esophageal proteins and showed that the majority of encoded epitopes elicited high titers of antibodies in recipient rabbits (35). The data from both strong and weak reactor proteins displayed in **Table 1**, can be combined into constructs for expression and vaccination. We suggest that simultaneous direction of the immune response to multiple targets on, or released by the migrating lung schistosomulum, will maximize the host response, so blocking its attempts to traverse the lung capillary bed.

## Author’s Note

The recent publication by Wendt et al. (Science. 2020 Sep 25;369(6511):1644-1649) has identified Smp_147680 calumenin as expressed exclusively in muscle cells, suggesting that it is a cell leakage contaminant of vomitus. This observation would explain its very high PSA score; as an internal protein it has not been selected for immunological silence by the immune system.

## Data Availability Statement

All datasets presented in this study can be found in the **Supplementary Material**.

## Ethics Statement

The animal study was reviewed and approved by Committee for the Ethical Use of Animals in Experimentation of the Butantan Institute and the Biology Department Ethical Review Committee at the University of York.

## Author Contributions

LF, GV, KW, LL, and RAW conceived and designed the experiments. LF, GV, PC, JV-S, AW, LN, WC-B, and RAW performed the experiments and collected data. LF, GV, AN, SM, KW, LL and RAW processed and analyzed the data. LF, LL, and RAW wrote the manuscript, and all authors critically revised the manuscript. All authors contributed to the article and approved the submitted version.

## Funding

This work was supported by grants from Fundação de Amparo à Pesquisa do Estado de São Paulo to LF and LL (2012/23124-4) and to LL (2017/24832-6), Fundação Butantan, and by fellowships from FAPESP to JV-S (2012/18.095-5), from Conselho Nacional de Desenvolvimento Científico e Tecnológico (CNPq) to LL. Part of this study was financed by the program: Coordenação de Aperfeiçoamento de Pessoal de Nível Superior—Brasil CAPES) –Finance Code 001.

## Conflict of Interest

The authors declare that the research was conducted in the absence of any commercial or financial relationships that could be construed as a potential conflict of interest.
