# Supplementary material for: Epitope Mapping of Exposed Tegument and Alimentary Tract Proteins Identifies Putative Antigenic Targets of the Attenuated Schistosome Vaccine
Source: Front Immunol. 2021 Mar 3;11:624613. doi: 10.3389/fimmu.2020.624613 (PMC7982949; doi:10.3389/fimmu.2020.624613)
Supplement: Supplementary file 1 [file DataSheet_1.zip › Supplementary Material/Supplementary Figure 1.pdf]

Supplementary Figure 1. Proteins sequences printed on arrays.

Array 1, Short Alimentary Tract, 15mer peptides, 1 amino acid offset

>Smp_194910.1_Saposin_1 159 AAs
NEENIYSEQLERPQCHLLCGLCRRIGRLIKIFLEDEPFIPLTSRVLYQMCKLIPERHWRNECLDITHY
LPRKIHEFADHINVTLECSELGFCHHKHTMLSNSEITSCINDHINYGLSLANSPKYKETIIKNICTHH
AADKHKCFETIESIITFLLEITI

>Smp_130100_Saposin_2 109 AAs
FKVSTVEENDYEMQNVTLECRLCLSGLSLAHYFMDDLYWRDVYMMGAKKLCTFISSEQIKNICNKYTS
KYLPKILDEVGSLIGPGICTDFDVCKSTEVKMFTIQENNKI

>Smp_105420.1_Saposin_3 178 AAs
INQPELEFGYKDVACNLLELAQEKSLQIMKEEQFIGNMVEYLVSITCDNVKDLNKRLQCKTSMATEAR
ILVQYFIEFIESYRLKTMLNWCQSTLERPQYGNSSFLCSTCEMAVAYLKTFSKSEEAKAIVHQAVDKI
CSLTGSFEVQCSFLGGMFIDKYIDTISTMDPDSACMTMHMCS

>Smp_105450.1_Saposin_4 108 AAs
YSVKNVDMSNTKETVDYQLDNNSTCSMCIQFITKWQTYLNSTSVDEKIEQFIRSTCSFYFFFRYRCEE
FMERYVNKTVYIIQHNNATTLCKFSIIECMLIILKLTPLC

>Smp_016490.2_Saposin_5 175 AAs
ESIESSINEFEQLHCQLICETCVMNVRGIRWLLLQSYTRKTIAELLSVLCNFFPYKQPRAQCKRFFKG
PFEGLVHDFVVNMSVYEPCQYLGLCETVQEELMKSPDDSFYTNLLMKTLSIVKEDIESIITPESIEQA
ATKLCSSDNTCIRTFEKSAIDFIRVITKNSEGEWFSDDF

>Smp_014570.1_Saposin_6 196 AAs
STESNKINLLTKENKDDNSTIAECDTCLAGMNLVHYILSEDYWVKIYMIAAEQICQSIASESLKNTCQ
AYVNNYLNKTLDVLAKAVNPDYICKALQACANDTRSSSTRNSGDSILCEGCKSVYNRIQSIFTDHTVI
KEMKSSVKVICLLYAADESQCDKVLQKYMDQAITYFEHHRADQTCYALCTFLKLFKLIFY

>Smp_085840_SmMEG-4.2 88 AAs
DIEPRIQKEYYYNLHENNSQANHNKFHEMPEYDDQLPDFPHKQLEEEQNPFHKLSEVLNSGSVVPLWL
VNPIYYVLELFPRAISYYFN

>Smp_010550_SmMEG-15 148 AAs
QRDPPRTNNTITHTTNHYVGKLSHHNTVPAKTTRKSQHTTATARHHNTLKTTLSHHNTVPAKTTRKSQ
HPNTTPSHTDKTVQKKCLNKMTPQDLISLLFSLIPQIKTIEFSQNENLLKLATILEKIFEQQSRVEHS
SPTKTPANKIFH

>Smp_178180_SmMEG-8.2 120 AAs
GTVSKPTATVKPQPVNKMNTTPVHQEEPSFWRRMWNSFTSMFGSSDSSSGTNNKDTKSPNPNTTEAKS
LSLKERIMNKFNSIFGEEEYNPPKDSDFTERLWMLFKHCFLNFKNLAKIFST

>Smp_163630_SmMEG-4.1_N_Terminus 55 AAs
SPLDDRFNDVNTINKKQFTEEEFSRLINSMLKKYEDKNVDIRIIGNKKDKQPTQK

>Smp_163630_SmMEG-4.1_C_Terminus 58 AAs
KPKSIADIFLINKPKVPLWIVNPLYYMVEKFVQIMGYLLEDDDTLELNLPKYYYDKSI

>Smp_171190_SmMEG-8.1 172 AAs
KFEHTTSGIRNPSKLSDSNASKTLSLKNLTDHYIHTPQKSNNGTSCNGKDTCKLPNPSQKGFTNTTSL
PHTQSHNSTVAPSVPKPTRQEIPRSGTIVNGTKPTPGKPVVNGTKPTPGKPESFLKRVGDGFFDLFSE
QEFHPINHKSYLFNFWYLFRTSFLNLKNMKNLLLGS

>Smp_152630_SmMEG-12 41 AAs
ENYEQQLQQPKAYGIWSLFSYFYKTFKVFCSVSNMVNWIFG

>Smp_125320_SmMEG-9_ 52 AAs
FVVHESSTEGQNHEESQFFLAPLAAAAGAHFLQFLNGCFLNMDNLKKLVFPG

>Smp_063530.1_apoferritin-2 174 AAs
PIQIHGEVEDPIALDNQVEEGLNDQILNEYEAFYIYDHMVSYLSRPEVGLSGFAKFFRESANEELEHA
RKFSEFVNKRNSKVVLKNILLASSGVPMDFKNIHEVIDTAIGKELEVTAHINALHKLASKMNDATTQD
FLDDFLQEQVNSVSKLREMRARLHLDNSAVYLMDKEFR

>Smp_147680_Calumenin 301 AAs
KPHLETVSRRDTEHFADDPNRHDIEFDHNAFLGEETAKEFSQLTPNESEEQLKIIIRKIDKNNDERIT
EIELKSWIEYVAKKSKQNSTDRQWNDINPTNQPVIKWTEYLMRTYGPEEERLKDTATSESYKKAVQHD
RRRWIAADLDEDDSLNKTEFTDFVHPEDRPNMRDAVIDELLEYVDKDNDGYVSEKEYLVDLARAYQST
PFDENEPEPEWVERERSQFRRFRDTNQDGRMDRAEVGEWIMPSNYDPIDAETKHLFYHADTNKDGLLT
EAEIIAKRDTFVSSQATNYGNALKQHEEL

<u>Array 2, Long Alimentary Tract</u>, 15mer peptides, 1, 2 or 3 amino acid offsets

Transport proteins, 1 Amino acid offset

>Smp_047660.1_Ferritin-2_heavy chain now Smp_311630 181 AAs
MNKTVSKHIFTESRARQNFAKECEDAINKQINMELQAAYDYMAFFTYFDRDDVSFPKAAEFFRKASHE
EREHAENLAKYQNKRGGRVQYSDIKCPTKVEFSDLVDAMNTALSMEKAVNDSLLKLHEIAAKNNDPAL
TDFIESQYLHEQEDAIKQFADYVTETNRVGSGLGQYLFDKMTLKE

>Smp_194840_Niemann_Pick_C2 129 AAs
EPFRDCGSKFGKLYSLTVTPCDRTPCALYKGQNATITIEFTTQETVKDGHISVHGVIAHVPIPFALDN
SNLCEFVSPTCPLIPSIGKYTHTYSLYVKAMYPSISLTIRWELQDSSNEDIVCVEFPVQLI

Enzymes, 2 Amino acid offset

>Smp_075800_Asparaginyl_endopeptidase 410 AAs
QLDTNYEVSDETVSDNNKWAVLVAGSNGYFNYRHQADVCHAYHVLRSKGIKPEHIITMMYDDIAYNLM
NPFPGKLFNDYNHKDWYKGVVIDYRGKKVNSKTFLKVLKGDKSAGGKVLKSGKNDDVFIYFTDHGAPG
LIAFPDDELYAKEFMSTLKYLHSHKRYSKLVIYIEACESGSMFQRILPSNLSIYATTAASPTESSYGT
FCDDPTITTCLADLYSYDWIVDSQTHHLTQRTLDQQYKEVKRETNLSHVQRYGDTRMGKLHVSEFQGS
RDKSSTENDEPPMKPRHSIASRDIPLHTLHRQIMMTNNAEDKSFLMQILGLKLKRRDLIEDTMKLIVK
VMNNEEIPNTKATIDQTLDCTESVYEQFKSKCFTLQQAPEVGGHFSTLYNYCADGYTAETINEAIIKI
CG

>Smp_139240.1_cathepsin_S 325 AAs
ELYNQSWDNNNNNNNNTIDYYLLWLTWQLRYNQYYQKTDELKDFYYWQLNIQKILLHNLYYDLGIKTY
RKSINQYTAINWNEFYNQKIKKNNQYFKNTKKMNKLRNNNINNDNNIPEYVDWRNNDTVTPVKTQEDC
ASSWAIASVEALEGQVKIKTGTLTPLSSQQLVDCAGDHECVENPVSVAFDFIKQNGVESQQDYPFTGK
VGNCTYDSSKKVTTISSYIQVDDNEEELQKAVYNIGPIAVRIAMTQEFLTYGSGVLLIDDCQNEEPFE
SVLVVGYGIENDIPYWLVKFNLGEEFGDHGYIKLARNYKNMCHIANFAYYPVI

>Smp_067060_Cathepsin_B1.2 323 AAs
HISIKNEKFKPLSDDIISYINEHPNAGWRAEKSNRFHSLDDARIQMGARREEPDLRRKRRPTVDHNEW
NVEIPSNFDSRKKWPGCKSIATIRDQSRCGSCWAFGAVEAMSDRSCIQSGGKQNVELSAVDLLSCCES
CGLGCEGGILGPAWDFWVKEGIVTGSSKENHTGCEPYPFPKCEHHTKGKYPPCGSKIYKTPRCKQTCQ
KKYKTPYTQDKHRGKSSYNVKNDEKAIQKEIMKYGPVEASFTVYEDFLNYKSGIYKHITGEALGGHAI
RIIGWGVENKTPYWLIANSWNEDWGENGYFRIVRGRDECFIESEVIAGQIN

>Smp_013040.1_Cathepsin_D 415 AAs
EVVRIPLHPLKSAQRTLIEFETSLEIVKKVWLSRVSGVDPQPEYLKNYLDAQYYGDITIGTPPQTFSV
VFDTGSSNLWVPSKYCSYFDIACLLHRKYDSSKSSTYIPNGTEFSVHYGTGSLSGFLSTDSLQLGSLS
VKGQTFGEATQQPGLVFVMAKFDGILGMAYPSISVDGVTPVFVNMIQQGIVESPVFSFYLSRNISAVL
GGELMIGGIDKKYYSGEINYVDLTEQSYWLFKMDKLTISDMTACPDGCLAIADTGTSMIAGPTDEIQK
INAKLGATRLPGGIYTVSCGNINNLPTIDFVINGKAMTLEPTDYLLKVSKMGSEICLTGFMGLDLPKR
KLWILGDIFIGKFYTVFDMGKNRVGFAKALHPDSVHRTKTYIPMMRLFPAQSVPQAASETPNGVFAFS
KLLSDEV

>Smp_141610.1_Cathepsin_B2 347 AAs
MNQYSCYLLQLYIIILLSYGTLNEIDARRHKRMYQPLSMELINFINYEANTTWKAAPTTRFRTVSDIR
RMLGALPDPNGEQLETLCTGYISDELPKSFDARVEWPHCPSISEIRDQSSCGSCWAFGAVEAMSDRIC
IKSKGKHKPFLSAENLVSCCSSCGMGCNGGFPHSAWLYWKNQGIVTGDLYNTTNGCQPYEFPPCEHHV
IGPLPSCDGDVETPSCKTNCQPGYNIPYEKDKWYGEKVYRIHSNPEAIMLELMRNGPVEVDFEVYADF
PNYKSGVYQHVSGALLGGHAVRLLGWGEENNVPYWLIANSWNSDWGDKGYFKIVRGKNECGIESDVNA
GIPKIKN

>Smp_139160.1_Cathepsin L_SmCL2 313 AAs
QHLSLQYDDIWKQWKLKYNKTYSDSNEIRRKAIFMRYVEKIQQHNLRHDLGLEGYTMGLNQFCDMDWE
EIKTIMLSKVFGNSPLWDDKKEELELSNDPLPSKWDWRDHGAVTPVKNQGLCGSCWAFSAAGAVEGQL
VKKHKKLISLSEQQLVDCSYKYGNDGCQGGTMDQSFAYLEKYPIESEKDYKYIGHDSSCHFRKSKGVV
KVKKFVDLPARDEEKLQKALYHYGPISVAIDALDDLILYKSGIYESKQCSSFLLNHGVLAVGYGRENR
KDYWLIKNSWGTTWGMNGYFKLRRNKHNMCGIATNASFPLL

>Smp_008610.1_DNAse_II 359 AAs
LSCLDDDGHTVDWFVGYKLPKGYDVVFMNADQRNWKLSKSPINEKGMMKNTFESMFKLVDKPDSVIGM
YNDEIPKWTKPLGYDRENLWWGHMKGAFAFDDTNTGFWVIHSIPKLSYTNTSYVYPRTGHTYGQHFLC
VTLEKKHLKSLITQFALARPLFQGAHIASSLKSEFPDLAKLLQNQKVFTETQSNVIELQTAKRSFQLN
HFSKSSDFGKDLYADFVAPKLKKSLDTETWQHDGSIPSACHRQYSVKNIESIYIRATGTTIRNAQDHA
KWAVTVPSKKPTEDTNNWICLGDINRQPHQFERGGGTMCIQDQKLWQSFYDSVQRVEECSKTVYTIFA
KRLFTRLLTLIEIVQFFNT

>Smp_242990_VAL_7 170 AAs
YRWYTQNSELLALHNAYRRNIKYGNVRDQPQAMSMLKLTWSHKLAEMAQEWALQCVPRRSNMTMRKGS
KWTYVGQSIAFVPKVRQAASVWFEQHKNYNFENNTCEANKTCADYKQLAFADTTHIGCGYAMCFNLTG
LDKVFVVCNYGPGGKYANRQPYDPIYPEDPYYLP

>Smp_331380_MEG-8.3 96 AAs
MNTTIIGILCICTCSIITNANAVPTNPSTVVAESPNTSTVEAESLTFKEKIVELYKNWMNEKEFNPPK
ESEFYERFWELFKHCFLNSKQLTKILPF

Large Proteins, 3 Amino acid offset

>Smp_043390_Beta_D_Xylosidase 819 AAs
QIFRNLSVSIKKRLDDLISSLTIEEIVDQMANGGAGPTYGPAPAIPRLKIKPYQWRTNPNQGLCTSFV
SHINQAASFDILDVWKIAFANGLEMRAKWNQFSQQNNYRDNTGINVFAPTVNLLRHPLWGRNKETYGE
DPYLAAELARAYVHGISGWNVPNDGAIKVHLLTINPIRQHVLLVGANCKHYAAHSGPENDPVSRLSFE
ADVPEHDLWMTYLPAFRACMEAGAVGVMCAYSGVNGTPACVNHWLLDTVLRKQWKYPGFVISDEGALQ
FLISKHHVYSSLQEAALAAVKAGVNIENALPNAVNVYSELLNLTKSGNVSEDELRNLVRPLFLARILE
GELNSPEMDPYAHLLPSTVVKSERHKYLSLVTAAKSMVLLKNYEEFLPIETKPDANERPLKHVALLGP
FSTNISELVGSYTKHIDPNDTVPLDKALEKISGNVTASDICIDGGKCTMFDDLAIQEILSQPDIDLVI
ITIGTGRNVEDESHDRHNLSLPGHQNEFLLSTLDMAAGRGIIRRKPVPVVLLVFSSGPIDIEPAVKDE
NVKSIFWCGYMNDILGEVIVRVLIGNPGKQFGPYAPLLRLDPELSEIGMWGTDIDEGYWWIPAARLPF
TWYSSIDELAHITVYKMTNQTYRYLPKSCTKQQKGCKIPILYPFGYGLSYNMLSPSLSGIKYLKLELP

LSPVKPNQPITVYATVANVGAIACEEVVQLYIQWVKCGQTDITNEDQWECTVPNIQLAGFKRIRLDVG
EKKDLHFQISPDQLSVWSNQNKSMVPGQGTLRITVGGQQPDQPVTVGSNYLIGIVKIDHTKQSHGWKM
PGT

>Smp_162770_LAMP_N_terminus 48 AAs
DVYGFTDADGYKSSSQSSETEETEVTDYSDDIIFPVINVSYDNMTANG

>Smp_162770_LAMP_C_terminus 232 AAs
VHKFSLKSGNSYCFLFQSDIMFQVFYNSHSKLVKSYKFTLSNATINDNESVCSEHYTKLSLTFIPYGR
NEEHKWTLVLLFNRMEPNKTSNDATNVSSIYTLDSITLTYYMDKNLFPDSLTPGQYVNVSSNNMVFRI
PVGSYYSCLTVPEITLKGNEKIPNNCKLFMKNLKVEAFMNNSVEAFIGNETLCDSDVNVNNMVPIGVG
IALIVCIVVAITVFIVFNKRNRRSYTTL

>MEG-22_Smp_318200 89 AAs
TETESKVVYLTPVPHPSVYFDNPEVKFEDLTFTKFIRYIVKKLLWLFDNLILKPYEMEIKQHNTSQMY
KFRLPENKKSNPLKNNTTQKN

Array 3, Tegument Surface, short proteins, 15 mer peptides, 1 amino acid offset, minus signal peptides and GPI anchors

>Smp_138080_MEG-3.1 131 AAs
AQETRDAERECKKHCEGNNEYVTRYCGGLCSSNTGPQTFYCYLGCSHNASTQDDFDKCLPKCNDRVQL
TEENCRDDCGRVTSHHELCGDVCGGNHGGSFPLCLYNCDQEHPREYERGYDKCKTKCYAMEGR

>Smp_138070_MEG-3.2 125 AAs
ARETQQECVRHCGGHNEYVTRYCGGLCSGSTGPQTFYCYLGCSHNASNQNDFDKCLPKCNGSPQLTES
SCQNDCGRVTTHPELCGIVCGGNVGDSFPLCLYNCDQGNGSGNFDECKTKCYEMAGR

>Smp_195190_Sm13 87 AAs
EPEPEPEPVPVSRNSKDVSIQTDVDLDPRFLLLDLKREIGRLKDTFNALVAKIDTIPPSSIATKYIHN
GLLSSICIIFTVYYHYKKS

>Smp_072190_Sm29 gi|4090941|gb|AAC98911.1| 165 AAs
VRCYVCDYCPIVTSVSISEENNCTSCSTAGYNYSIHRICVFKDGIPINFPNENRTQCNTDLCNGLTVD
NTGKIPSVPIANPFRCYTCLNCTKSNQKVLSGCGACVTTRGSGIISKFCGTTCERLYIDDQISCCSTD
LCNG

>Smp_335630_SmTSP-2_Loops 84 AAs
LHSVVNHWSEIEPPAIVEKPKVKKHITSALKKLVDKYRNDEHVRKVFDEIQQKLHCCGADSPKDYGEN
PPTSCSKDGVQFTEGCIKKVSDLSKAHLNAI

>Smp_025830.1|ADP ribosyl cyclase 284 AAs
QHQINLLSEIVQSRCTQWKVEHGATNISCSEIWNSFESILLSTHTKSACVMKSGLFDDFVYQLFELEQ
QQQQRHHTIQTEQYFHSQVMNIIRGMCKRLGVCRSLETTFPGYLFDELNWCNGSLTGNTKYGTVCGCD
YKSNVVHAFWQSASAEYARRASGNIFVVLNGSVKAPFNENKTFGKIELPLLKHPRVQQLTVKLVHSLE
DVNNRQTCESWSLQELANKLNSVHIPFRCIDDPLEFRHYQCIENPGKQLCQFS

>Smp_194860_LMWP 73 AAs
ESQASQKELFTESVKLWKSITELWKRFEHNCRVKIRKYLEEDNLGEKLAAVVSIYVKRLNKRLDMRLS
EDRAE

>SmMEG-24 44 AAs, Not on Wormbase or GeneDB
VILHPKYEMTFTEKLRFYFRCLVKVLCHMIRLGYIGPLSDYYVH

>Smp_152580_SmMEG-5_ 56 AAs

SGRQPKFVNVDTDGNLRSGGSSDISDMFGQNKTLGTAFKTLLHNLWDLLKQSLGLP

>Smp_195180 Sm25_ 111 AAs
EENSNSIITDEDYDHYNSSLDSSNNVKHSQEAFHRNSDPDGFPEYEFLNETSIEIKEELGQELHQLQL
ILDELSRRIRATPNSANKYMKNEFLMSSCIVITLNLFIFMYKS

>Smp_168730_New Carbonic anhydrase 306 AAs
NGSEWSYTNILTGPETWHEHYKNMCSGYYQSPIDLKTDISTLDLKLKTVIIYRNTSSTETTTIQNNGHSAEVKFP
RNTWFISFDGILDYKYEIIQMHFHWGNTDDRGSEHTIDGFRFPLEGHIVSFRRQMYSSPSEAIGRPGGLAVLGIM
HQIVESIKYEQTAFKAYNNFSGVLNSQFVPPNNSTIDDINLALLLSLLNPSRYFRYLGSLTTPPCTENVLWTVFI
DPVLITREQINLFRNLPYGSNEKQTRMGDNFRPIQLLNPIDTLAS

>Smp_138060_MEG-3.3 131 AAs
AQQECEKNCKGDNEYVSPNCGILCSGTIGPQTFYCYLGCSHNATKQSEFDNCKTKCDGGVQLTKEACL
SNCGLITTHPELCDAVCGGNDGGSFPICLYNCDQKHTDPRKDGADGSEDFDKCKTKCYKMAGQ

>Smp_019350_CD59 73 AAs
HRCYVCSKCQDPFRVKDTEIQNGCTFCSTIRTYVQDKLQVTSRSCVPVCVEADARRSGSGIVTSCCQD
DLCNS

>Smp_105220_CD59 105 AAs
IKNKKVKCYRCSDCPNPFDKTQITELGNCNFCRTVYTYRDEDNYRIAKDCVASCVPQDRRGGKAGLVT
ECCDEDYCN

>Smp_194970_tetraspanin_loops 106 AAs
PIRTLDVLANYNTILDYFKRSSLRKNQFFKVANQSLWNSIQYYEKHPNYENQVDNLQREFFCCGVRSY
TDYKRPVITLPLSCKTGNSIHPKGCAEALYDYIQHCIM

>Smp_005740_Aquaporin_external_loops 74 AAs
GDHGKHAHGTREKIIEYAKLNDNGALLLNTTGGIFVTNPSASHLTCFNPARDLGPRLMIFIFGWGNKA
FSGANY

>Smp_163710_MEG-6 64 AAs
MVQNPKNTKKINPIRRSTKTVIVITDRVQNIVLGHRLLHHRIPTIKRSKSHGINKNETVSNLFP

2 amino acid offset
>Smp_074140_annexin 347 AAs
MGRDKSQIIGPNGETYHPTLKIDLHNDPKKDAEELYQAMKGWGTDEHRIIKVLGYRNSYQRMEIRDTF
KAMYGKANLIDELCSETSGDFRRLLKMLLTDIDKVDARALYKAMKGGGTDEETIIEVLCTATNIEIEN
IKQAYLSDPSRTLESDVQSDLGGYLQQLVVALLQAKRDEIPFEDVEKISKKGLKSVVDMSQVEQDVEI
LWDAGEAHLGTDEDAIIRIVCGRSVWHLQEVSHLFEKKYGKTLVDSLASETSGDFESALLLILNTCLN
RPKAYSDLLVKAMKGAGTDDCTLMRIIVTRCEFDLGSICIEFQKSQGSTLEDWIRNETSGDYQRLLLA
LIGAEWQ

>Smp_122630_MEG-1 127 AAs
IVFKSDETVNHIKDIDWLILTKGGKLNRTWFVFNETKEICSCLTDFIKCIFREINIDKDYLCTYPTNF
SHGLITYCTKSNDERDLLSYEEDHIALYVIQPTNHCQRYEGSSEFGISKTRKRMSFLLR

>Smp_105410 Glucose transport protein SGTP-4 loop 33 AAs
ENIKEFLSRTMLGKNASEAENTANLVTPSFLYA

Array 4, Tegument Surface, long proteins, 15mer peptides, 2 amino acid offset, 6 proteins, minus signal peptides and GPI anchors

>Smp_155890_alkaline phosphatase 360 AAs
KSSLLNVADPETWKKSADERFNKFEKSLSYLLLKRPKNVILFIGDGMSLNTVTGARYLKAEKMDFLGG
DVQLVWENWPVASLVRTFNSDRLTTDSGSAATAFMSGVKGPFNTVGITGTVKCCECTELKELERAKSS
IMYASKAGFSTGIVTTTRVTHATPAAAYANMLHRDWESKVPSNEHAFHCTDAAAQLLTNASHVNVIMG
GGASEFYGPSDNTTFNVKGKRSDSRNLLNEWQEIQTKMNHKHVLLHTNNEFKQTDWSSVDYVLGLFAP
NHLAYRLENQDQPTLAEMTEAAIKVLSRNPKGFLLLVEGGRIDHGNHQNQAQYALTETLELEKAVEKA
LSLVDQQETLLLVTADHSHA

>Smp_214190_calpain 758 AAs
MGRIQIVYSPDENVSGRTNRPGKEVVDPRTGRIIKVKRETPDDYLNVLKPIKGPKRMEFNPYLPKTLT
PKGYAKFKLMMNVASKQYETLVKRLKTERTLWEDPDFPANDKAIGNLPDFRERIEWKRPHEINPNAKF
FAGGASRFDIEQGALGDCWLLAVVASISGYPQLFDQVVPKDQELKGPEYVGVVRFRFWRFGHWVEVLI
DDRLPVRQGRNTLVFMHSNDPTEFWSALLEKAYAKLNGCYAHLSGGSQSEAMEDLTGGICLSLELNQK
ERPSDLIDQLKIYAQRCCLMGCSIDSSVMEQKMDNGLIGSHAYSLTGVYPVNYRGRTQWLMRLRNPWG
DSHEWKGAWCDGSPQWREISEQEKKNINLSFTADGEFWMSYEDFVTCFSRVEVCHLGLESLEYNQNFH
GKRRLDEAIFSGQWQRNVNAGGCINNRTTYWTNPQFRITVEDPDPDDDDNKCSVLIGLMQTDIRKKVG
ADFQPIGFMVYNAPDDLNTLLSRAQLLTRSPIAKSQFINTREVTAQFRVPPGSYVVIPSTFDPNIEVN
FILRVFSQTSITEQELDEDNTNQGLPDDVIEALKLEDTLLDEDQEIEQKFLAIRDPKTNAINAVKLGE
LLNNSTLQDIPNFQGFNKELCRSMVASVDNNLTGHVELNEFMDLWIQAKGWKHIFIKHDVDQSGYFSA
YEFREALNDAGYHVSNRLINAIINRYQDPGTDKISFEDFMLCMVRLKTAFETIEAHPKNIEGTSLFSA
EDYLRFSVYI

>Smp_077720_annexin 1 340 AAs
MISHQTGPSITFPDRISAENDAEQLHNACKGLSTDEETITKILGHRNLQQRYQIRETFHRRYKKDLVH
VLCSSTKGDYESLIKTLFRGSIQILAHDLYKGLKKPDIVNEIICCCNNHEIMMLKKAYQEVLQEEEPK
KASQRTLESDIIKETKPPYEQLLVALLQARRDEDPPELVEEAIRTRSTSRLVSRSQVDKDVEDLYYAG
EKRAGKGDSDTFIKILTKRSKYHVKEIWDLYLAKYHNTIVEVISKKFSEPFRSGLNTMIMALMDLRLL
LVCQLYDSMYGLGTREDTLIRITCLRCEVDMNTLKSMYREYFGKPLIEAVREDTSGDFRKLLLALLGE

>Smp_074150_annexin 2 365 AAs
MANISGFGITRSLIHSFDPHGKHYRPTIKPTTGFSASADAERLHRSMKGPGTNELAIINILARRTNYE
RQEICQSYKSLYKQDLKDDLKSDTSGDFRKVLCQLIVDTPYMLAKSLYYAMKGLGTNDRVLIEIFTTL
WNDEMKAVADAYKQVLKDKGSEESERSLVTDMKKETCGDYEYALLSLVQAERDDIPILQLKAIPDKGV
NSIINHELAEADAKDLYASGAGRVGTSERRITRVICNRTPYQLYLTSEIYFKMYGKTLLEHIESETSG
DYRKLLVAVLRYAIDRPSLIAEWLHDSMAGLGTKDYALMRLLITRSEIDLQDIMDAYESIYGKSLLNA
VKDDTSGDYRRTLCVLMGEIYNQQQ

>Smp_017730_Sm200 1630 AAs
DLHSNNVVDIKAHDYKLLTKILAARQLQDLFDNDKNTHGLFHAELNQKVYLIVDLGGLYQVSSVEITS
DEPENLKQTISIGYGFDKNTTSLFGSLYNCTYQQTSTLCIPACSQYDNSRTGFVGNSLLWSFQSDKEG
WTRIYDLIIRGTPFDENRSMKNNLDDITLFKPYVDHLVPAESMESCNGLLWHLNDESSVTESSALAAT
DDRNVTIYFGKTYSVTSVHFVTSKRDDMPQEYTLHFNGMESLTKIINLQNDCTLTASSDNDGANFKEY
NCPTTDLESYTFDYVTVNVKGLYKLHVYGLPFHYPKIQIIPSKEDDKTIMVDKNVLQISCIAQSCNST
SNVLLDVDDSYRRSRSSDKSCPMNGHIVRCEYLNLFRLSTSPNDDNNQKIQLTNQGIINTEFSEIMTE
LRIIQSNAEKLIVTMPRTHQYYSQYECSCQATDNKKSQLFSLSTNLKPTDFDQDFIFQTNYTTIFADQ
TIESHVGFIELPENEHESYFKLNIIGYSLLNDVQIGVVFVEGGQAGSRTNANIEEISVQAFPGINVGS
DLWSIPVYKYELQDVWPDSVDDSVITVTWSMPKALSATKSPSVIRDIFRALIITSSGVNTIRAWVWQR
DSHLLDIRAYHQLDENNVDSQLKILTLQRSGCSPSESEDEVVASVQLKDGQCSTDNNDIITCTRTIHG
QIIQFKLNNPSTSDVYKLYMKSDGVEDNVESTSSIDLVTSGSLGETVKEDIKGAGLSLTVEGIHHNHE
TQETELDVAVHIASKVISDNIACRPTYLLLEFIEPNIETLKSRVSSKQTMFRIKLPSNQKEINLKMQL
SIGSVDPTQSEATTNQSIAFQNPFYIPTDIKVDAENQLIQWFGLPTIFNNLLHHYETKLSGLPKACEQ
ASEFNLPITQQEIDNGTIYRVNLKNIPDPTITKNGLAIDYNFKVTPVFKGIDGKSITMGTSSDIRFST
GRTGQTDLKAPTSGRYYSLQVQVRPSQIPSCLNLETLNTQFILRVIGEVDEYPDYIKQVNYVPITMKT
TETLNDKNNHVKLYKIENLLPGRRYELQAEVIYTEDFRDKISEPVRLWIEDEVHVQTEEVFISPGERV
VINCTGSVGPNDTSQKSLEWKLFDGGRLPDGSRSLKTQEAQSGPLWYAMESLIFDPVNKQHGGVYACF
IRPSILELMNKPTELHKVTVTVSDLEVDINSKIVEFGEKIIITCRTASPGQLDWMLPSGEKVEIMNEM

KSDDDNDDQPYTIKDENDDVKLSIKLIIPKVNLNKVGKYTCLHSPSNNKQTFSLKMKEVIKLVKSPES
SDKPGKTLILDCTANLGNLHQSVVWYKRPNSNSPWLEITEAIQTIEHITIQQKNPEDTLSSGVWLSEL
KVKNSPGIIGEFMCTIQNIQTTMNIERMETGSIMTNDNDFSKITHATIKVSLKSVLKILTPIKLENGQ
ISVHCQGYPAHSKDRLQWVYIPLNTDNKSDKVITIVHSNPKDEENDQESDGNTKSLDEKEIDEIVSLA
FQLTDSIPATWPGSTGPQQLVQSGLIEQEHQPKQMYTAERLSLIFDSKYAEKVADGILSCRYVRPKGI
LPMDSDEAAESLSKVTIPETNDDSDEILEKSEIPMKTLLDAKQGDDNDNLSILKSSLNEEPDEVQS

>Smp_042020_Apyrase_ext_domain 446 AAs
LYILSSNIPDVYSVVIDAGSTSSKLHLYKWIDEPFRSNGKVDEVTNEKLSPGISDYINDTIKAYDTLK
PKLLKLTNSLTFEQKQHTPIYLAATAGMRLKLIEDPLGSLDLFSVIRQYLKQSGFQIETPNERIRLLY
GSEEGLYGWVSVNYILGIIKEGKQTNPSDTVGSLDLGGASTQIAFIPKVYSNIPKEKLDFYPLRLYGN
DFSVYSHSFLCYGKSEFERRVITSIAAASLLQSKIPNPCFLQGYKSDLYNAFEWFSGSCLSGTYVKKT
FAEEIFRPPNMNSFSFNGTGQPNECVNYILKHFQTKCDHSSCSFNNVFQPAPFGKFMAYSGFSYVMRY
LFPNKNTGFTLTEVTDAVMKFCKKPWKDVAKITKLSDQGFTAKYCFDGLYIITLLKMYGFTTDESWKT
ITFDSKVNGKSVSWALGYMLDQSGHLPSESPKVSVSTQ
