# Supplementary material for: Epitope Mapping of Exposed Tegument and Alimentary Tract Proteins Identifies Putative Antigenic Targets of the Attenuated Schistosome Vaccine
Source: Front Immunol. 2021 Mar 3;11:624613. doi: 10.3389/fimmu.2020.624613 (PMC7982949; doi:10.3389/fimmu.2020.624613)
Supplement: Supplementary file 1 [file DataSheet_1.zip › Supplementary Material/Supplementary Figure 3.pptx]

## Slide 1
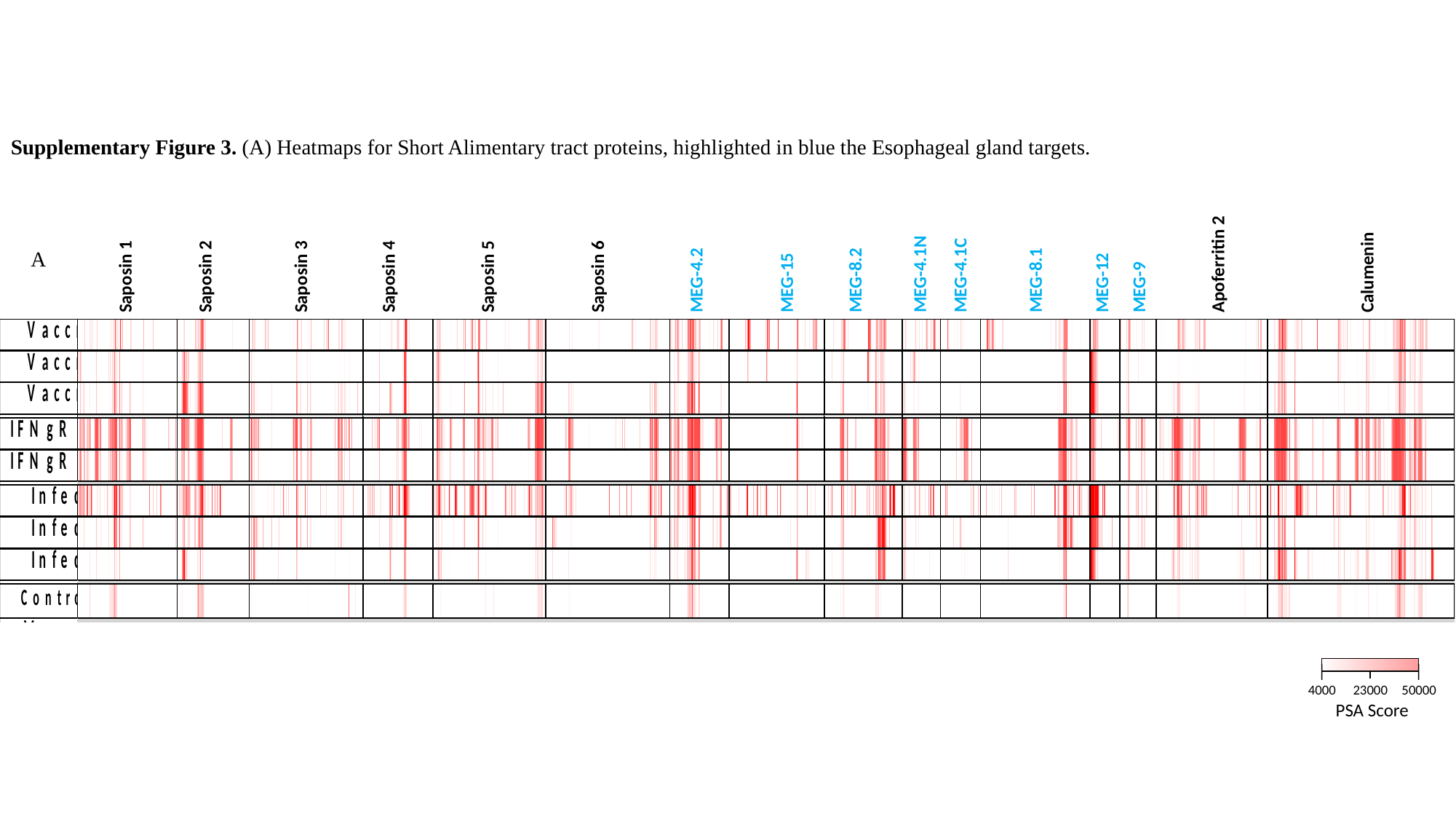

Supplementary Figure 3. (A) Heatmaps for Short Alimentary tract proteins, highlighted in blue the Esophageal gland targets.
Saposin 1
Saposin 2
Saposin 3
Saposin 4
Saposin 5
Saposin 6
Apoferritin 2
Calumenin
A
MEG-4.2
MEG-15
MEG-8.2
MEG-4.1N
MEG-4.1C
MEG-8.1
MEG-12
MEG-9
4000
23000
50000
PSA Score

## Slide 2
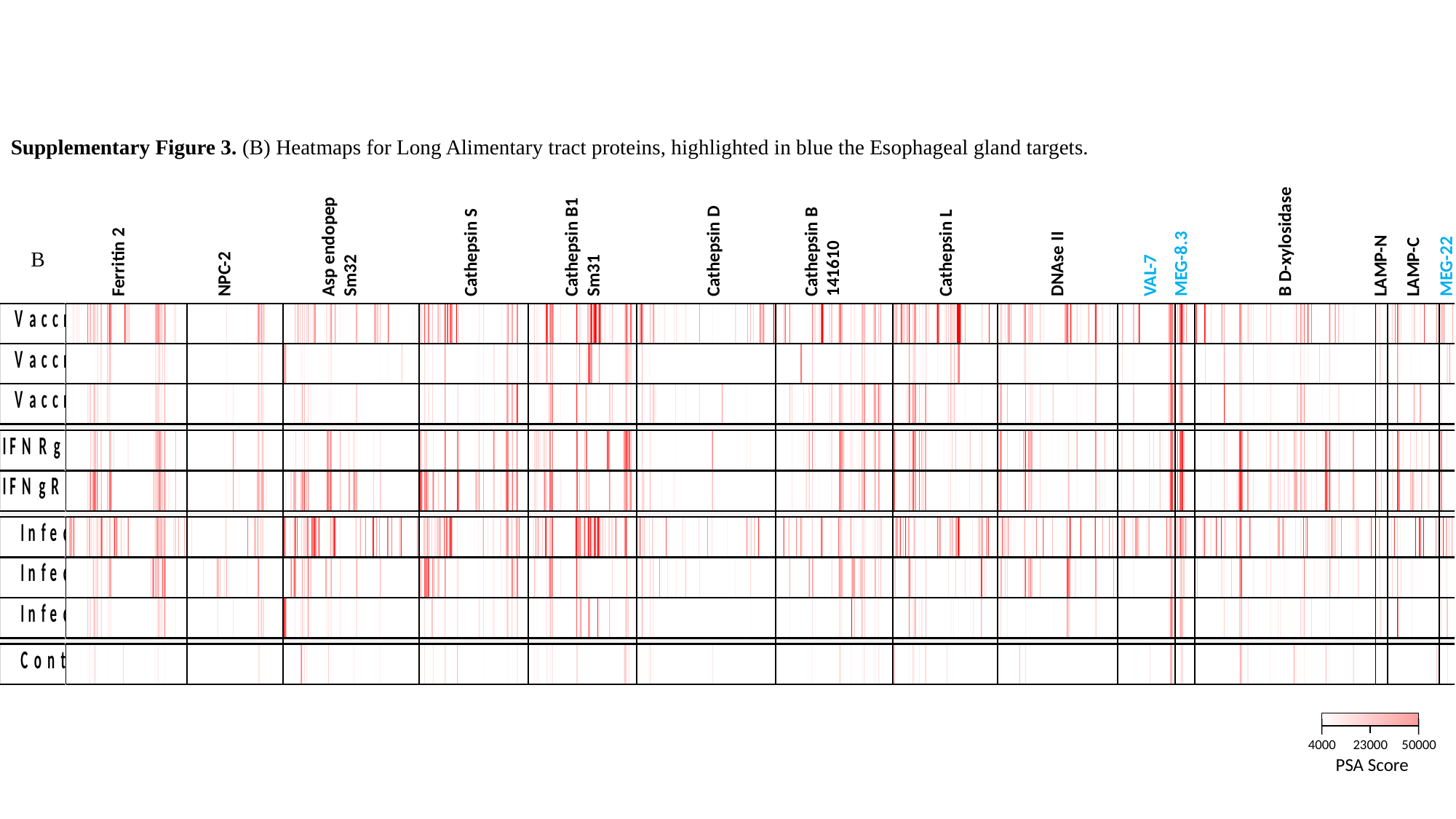

Supplementary Figure 3. (B) Heatmaps for Long Alimentary tract proteins, highlighted in blue the Esophageal gland targets.
Asp endopep Sm32
Cathepsin B1 Sm31
Cathepsin B 141610
Ferritin 2
NPC-2
Cathepsin S
Cathepsin D
Cathepsin L
DNAse II
B D-xylosidase
LAMP-N
LAMP-C
VAL-7
MEG-8.3
MEG-22
4000
23000
50000
PSA Score
B

## Slide 3
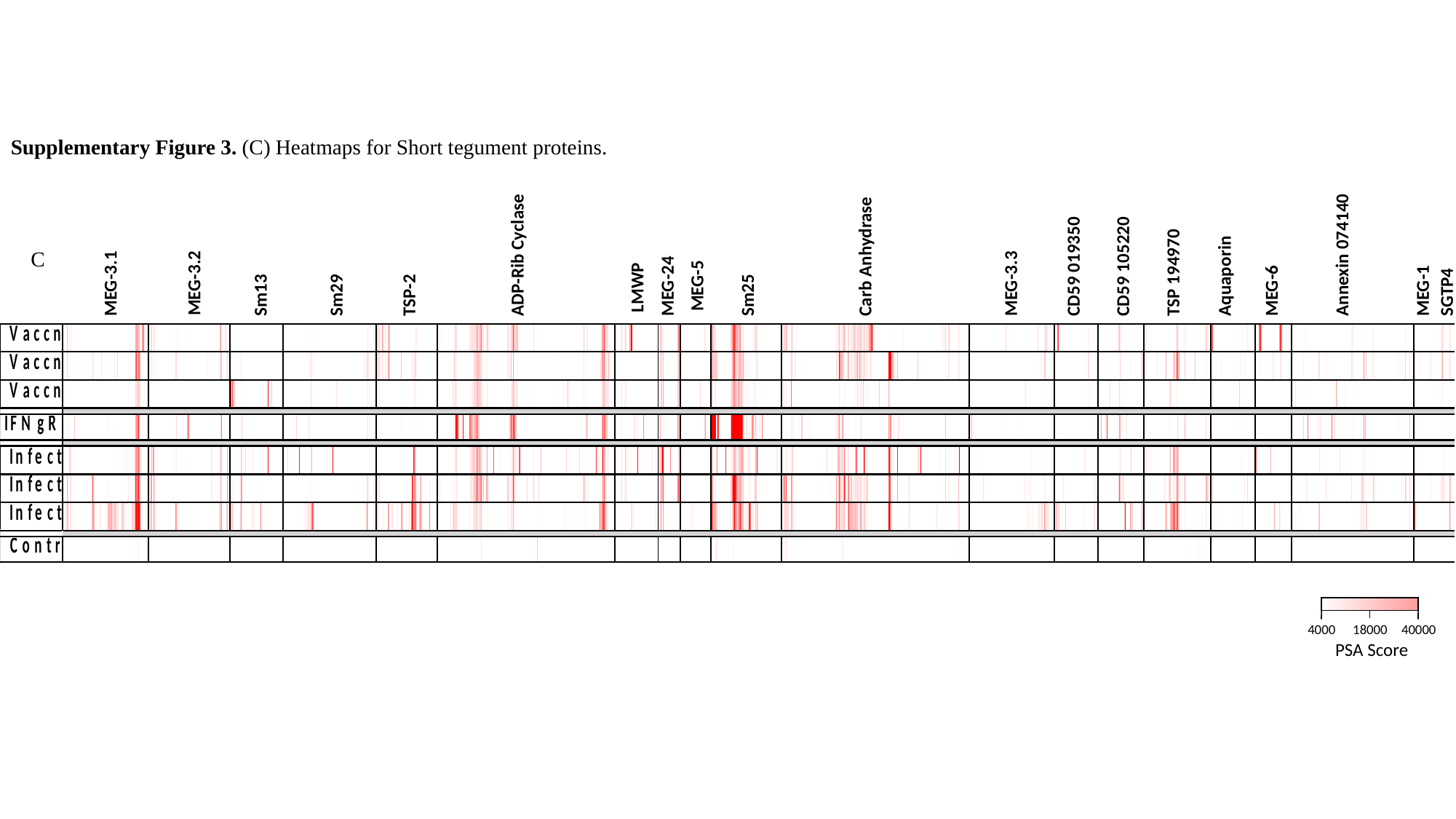

Supplementary Figure 3. (C) Heatmaps for Short tegument proteins.
ADP-Rib Cyclase
Carb Anhydrase
Annexin 074140
MEG-5
LMWP
TSP 194970
Aquaporin
Sm25
CD59 019350
CD59 105220
MEG-24
MEG-1
MEG-3.1
TSP-2
MEG-6
MEG-3.3
Sm13
Sm29
MEG-3.2
SGTP4
4000
18000
40000
PSA Score
C

## Slide 4
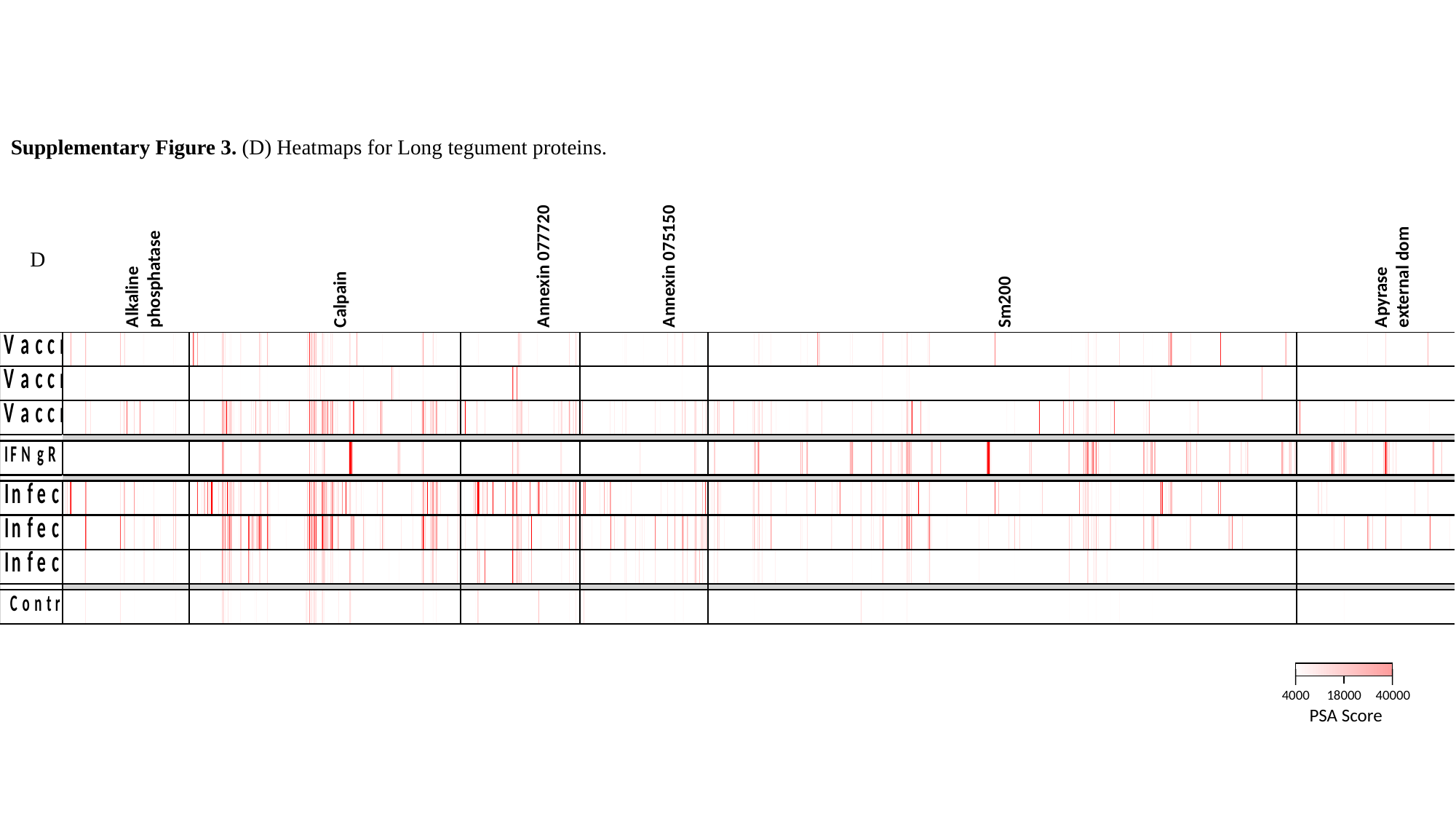

Supplementary Figure 3. (D) Heatmaps for Long tegument proteins.
Annexin 077720
Annexin 075150
Alkaline phosphatase
Apyrase external dom
D
Calpain
Sm200
4000
18000
40000
PSA Score
