# Supplementary material for: Epitope Mapping of Exposed Tegument and Alimentary Tract Proteins Identifies Putative Antigenic Targets of the Attenuated Schistosome Vaccine
Source: Front Immunol. 2021 Mar 3;11:624613. doi: 10.3389/fimmu.2020.624613 (PMC7982949; doi:10.3389/fimmu.2020.624613)
Supplement: Supplementary file 1 [file DataSheet_1.zip › Supplementary Material/Supplementary Figure 4.pptx]

## Slide 1
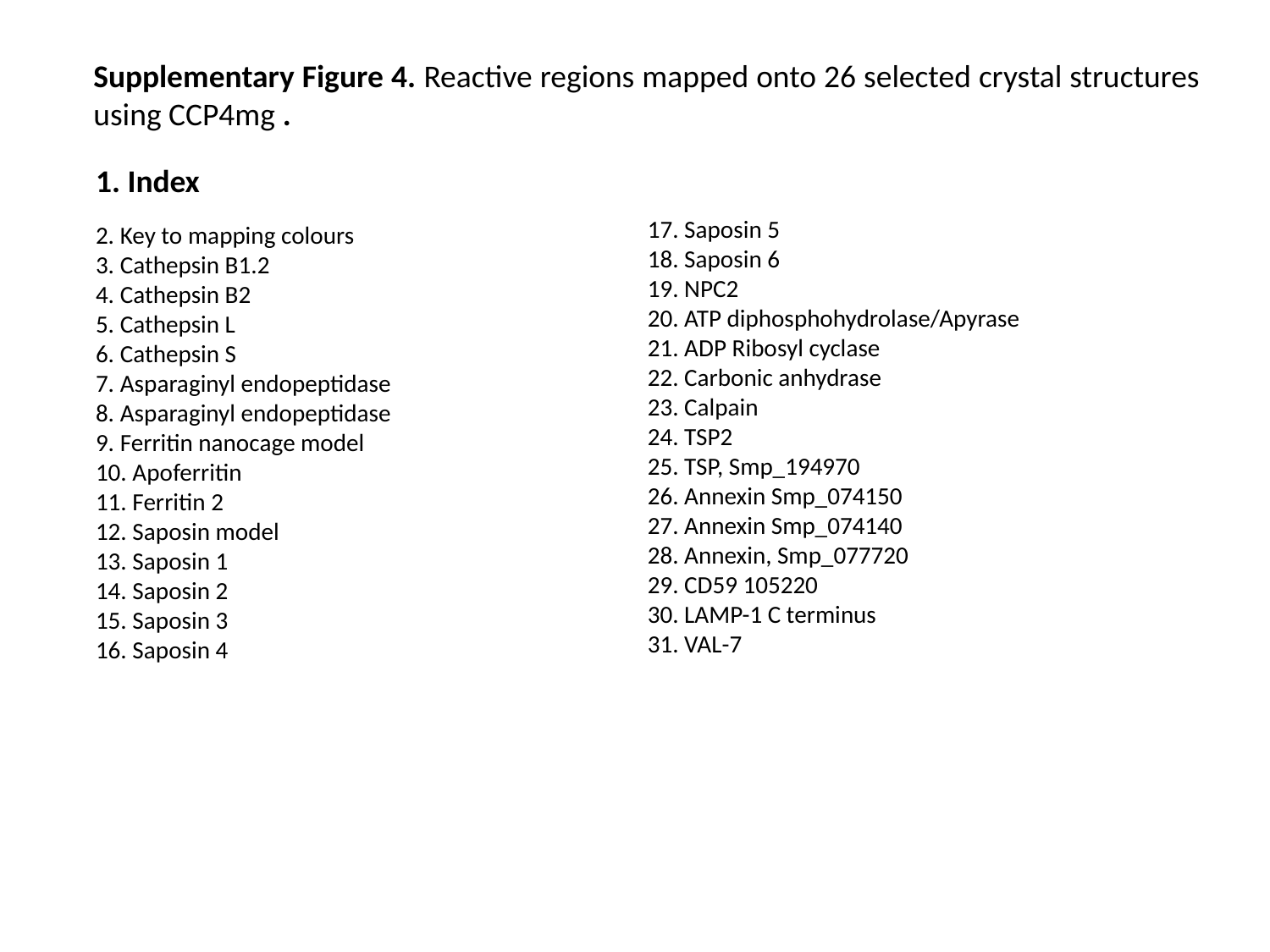

Supplementary Figure 4. Reactive regions mapped onto 26 selected crystal structures using CCP4mg .
1. Index
17. Saposin 5
18. Saposin 6
19. NPC2
20. ATP diphosphohydrolase/Apyrase
21. ADP Ribosyl cyclase
22. Carbonic anhydrase
23. Calpain
24. TSP2
25. TSP, Smp_194970
26. Annexin Smp_074150
27. Annexin Smp_074140
28. Annexin, Smp_077720
29. CD59 105220
30. LAMP-1 C terminus
31. VAL-7
2. Key to mapping colours
3. Cathepsin B1.2
4. Cathepsin B2
5. Cathepsin L
6. Cathepsin S
7. Asparaginyl endopeptidase
8. Asparaginyl endopeptidase
9. Ferritin nanocage model
10. Apoferritin
11. Ferritin 2
12. Saposin model
13. Saposin 1
14. Saposin 2
15. Saposin 3
16. Saposin 4

## Slide 2
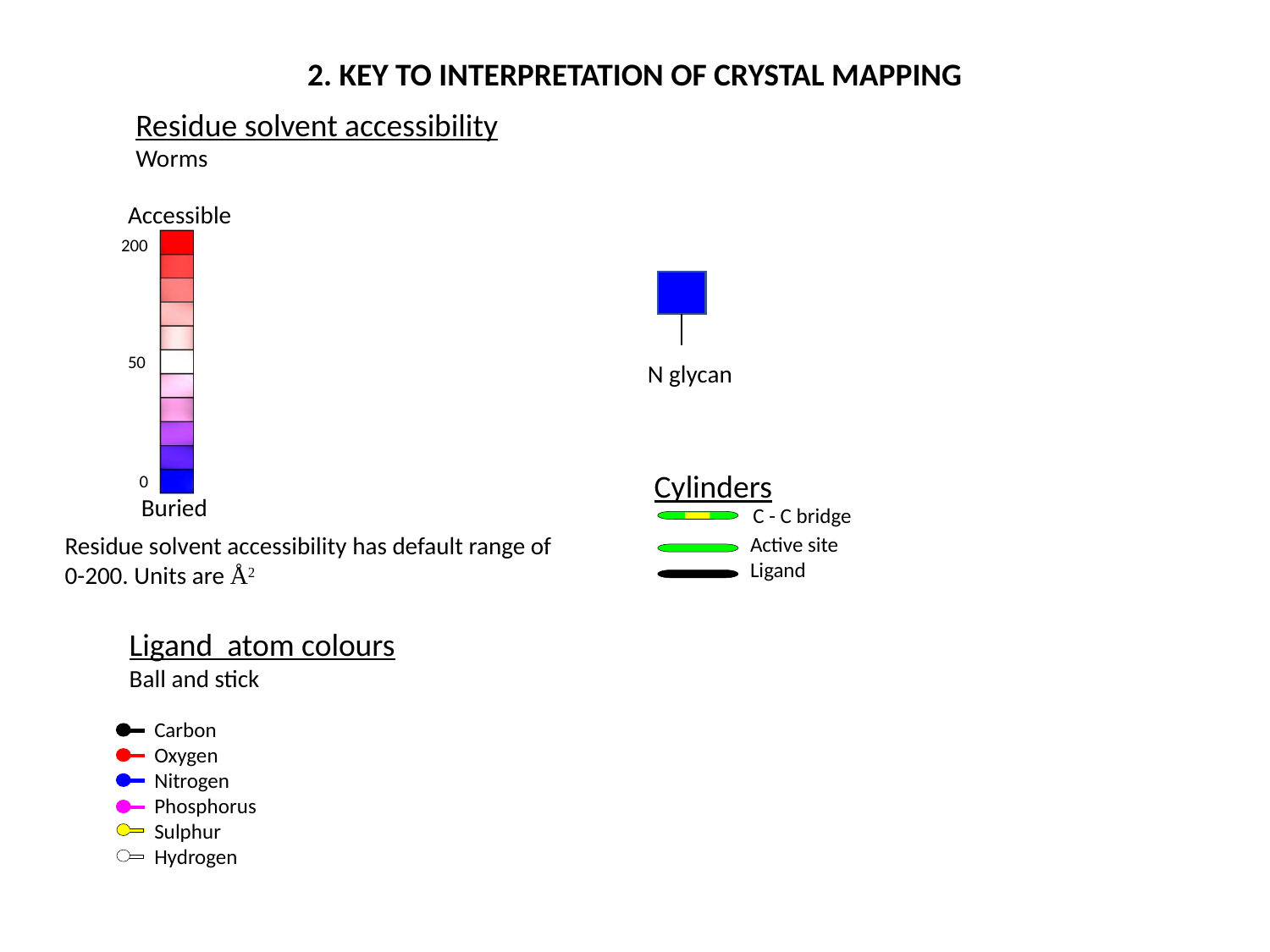

2. KEY TO INTERPRETATION OF CRYSTAL MAPPING
Residue solvent accessibility
Worms
Accessible
200
50
0
Buried
N glycan
Cylinders
C - C bridge
Active site
Ligand
Residue solvent accessibility has default range of 0-200. Units are Å2
Ligand atom colours
Ball and stick
Carbon
Oxygen
Nitrogen
Phosphorus
Sulphur
Hydrogen

## Slide 3
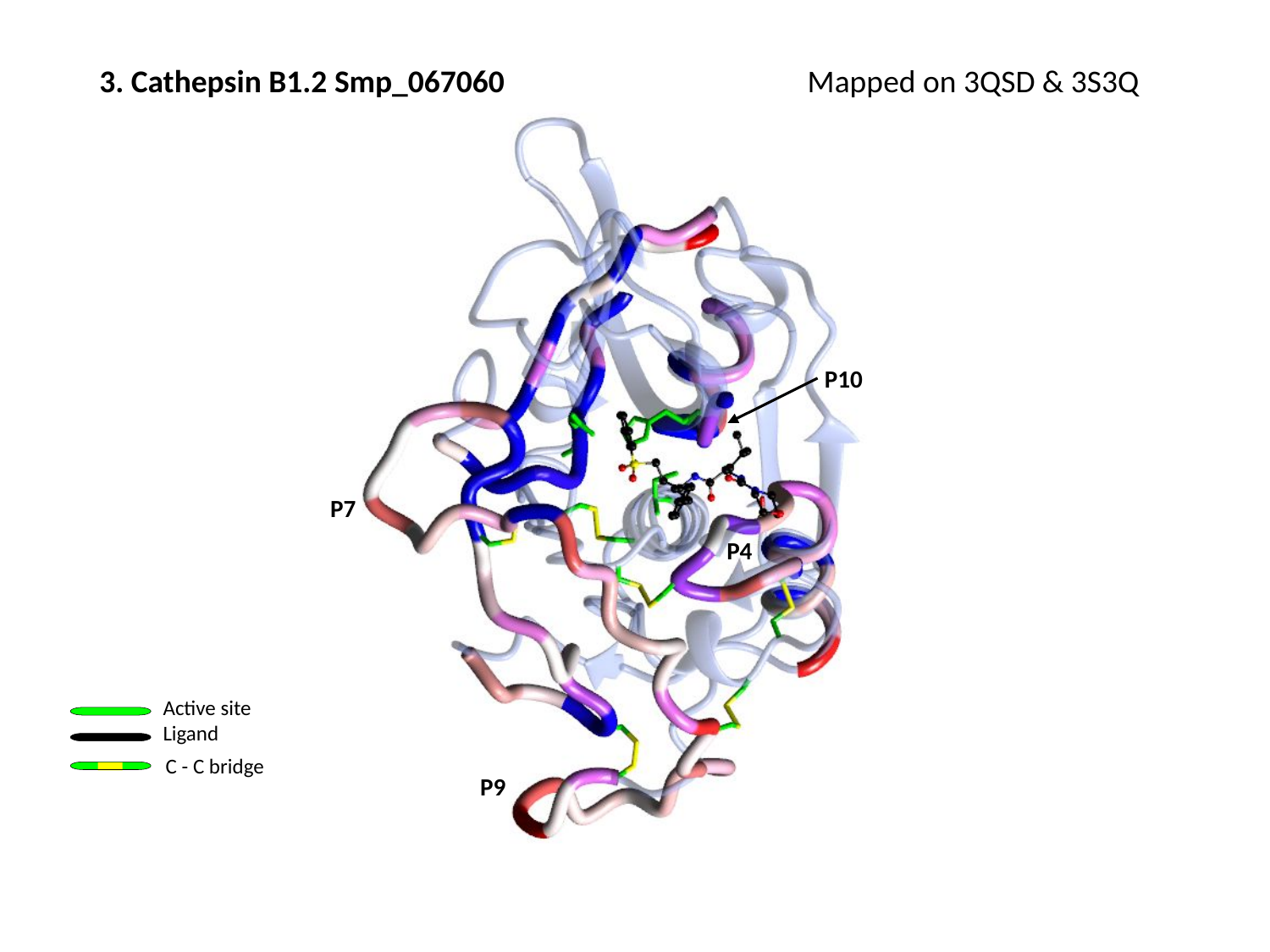

3. Cathepsin B1.2 Smp_067060
Mapped on 3QSD & 3S3Q
P7
P4
P9
P10
Active site
Ligand
C - C bridge

## Slide 4
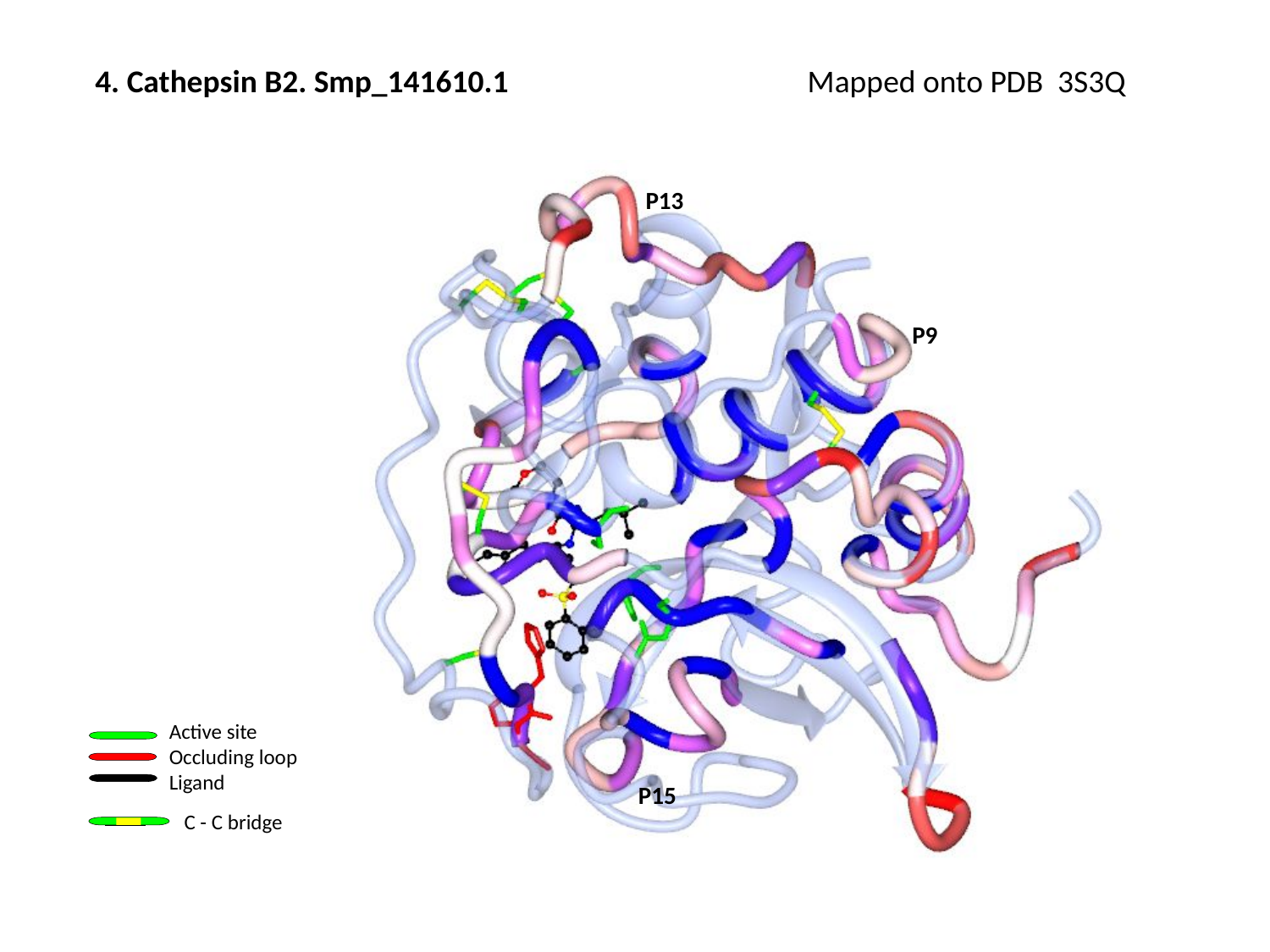

4. Cathepsin B2. Smp_141610.1
Mapped onto PDB 3S3Q
P13
P9
P15
Active site
Occluding loop
Ligand
C - C bridge

## Slide 5
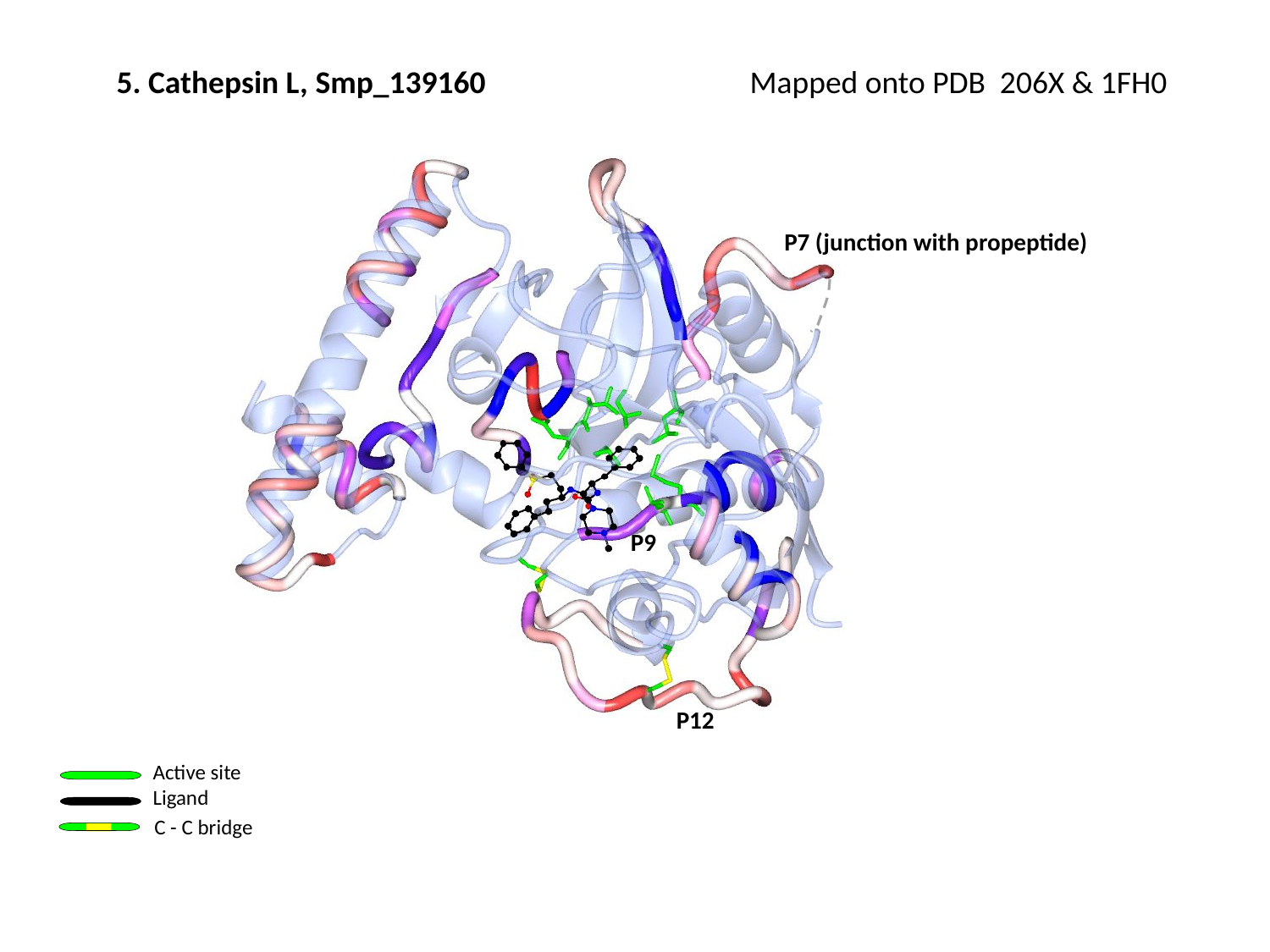

5. Cathepsin L, Smp_139160
Mapped onto PDB 206X & 1FH0
P7 (junction with propeptide)
P9
P12
Active site
Ligand
C - C bridge

## Slide 6
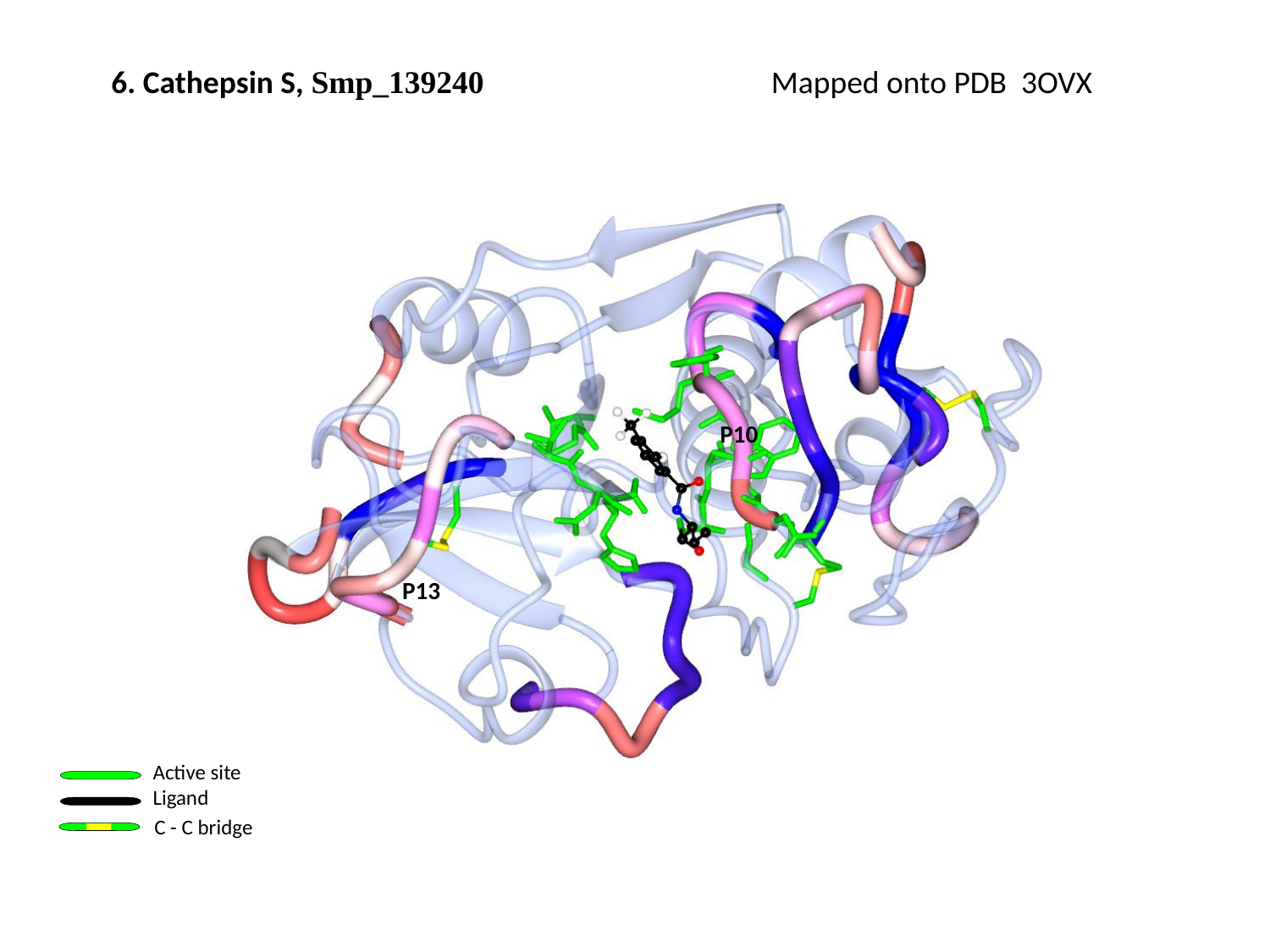

6. Cathepsin S, Smp_139240
Mapped onto PDB 3OVX
P10
P13
Active site
Ligand
C - C bridge

## Slide 7
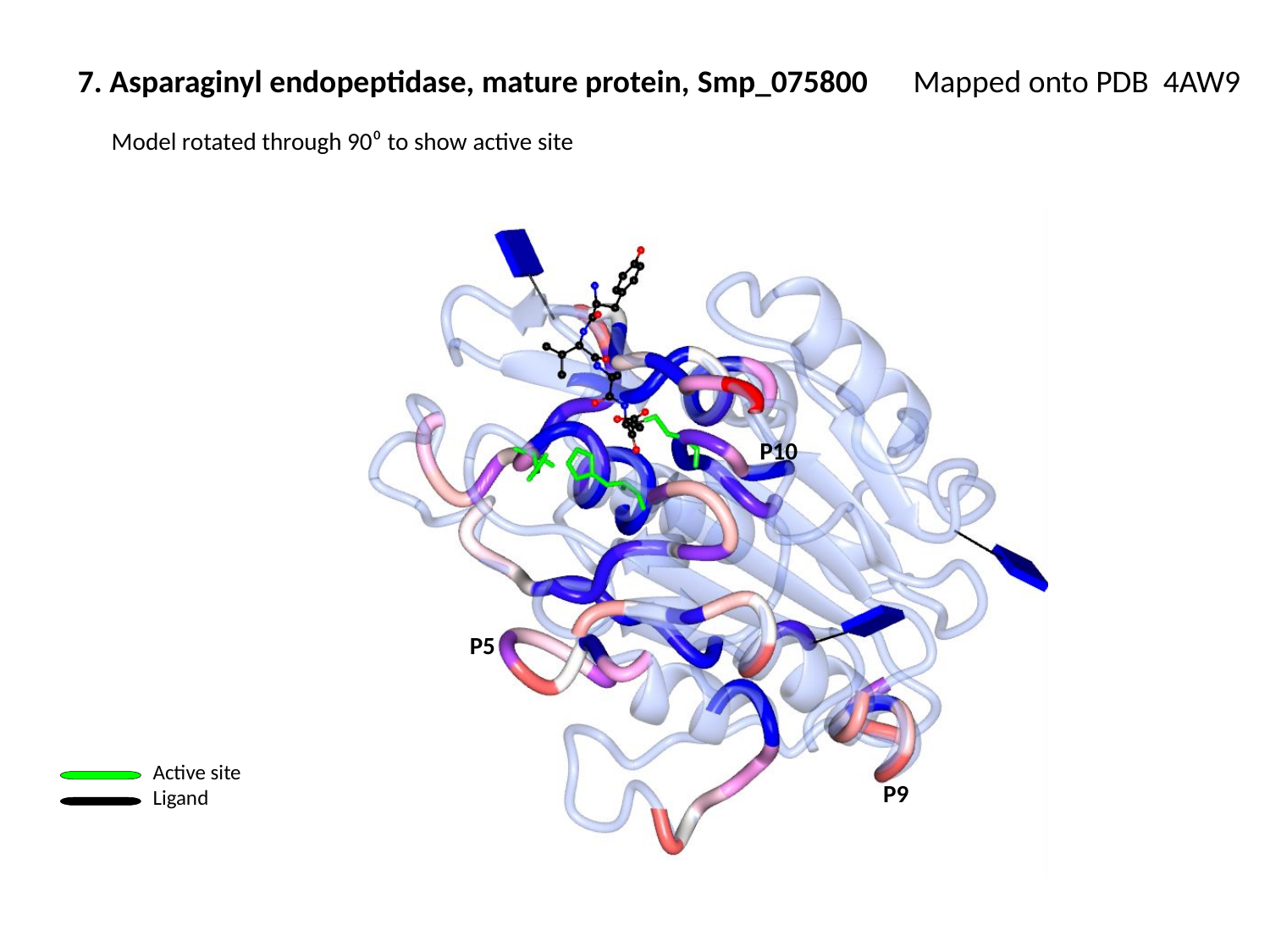

7. Asparaginyl endopeptidase, mature protein, Smp_075800
Mapped onto PDB 4AW9
Model rotated through 90⁰ to show active site
P10
P5
P9
Active site
Ligand

## Slide 8
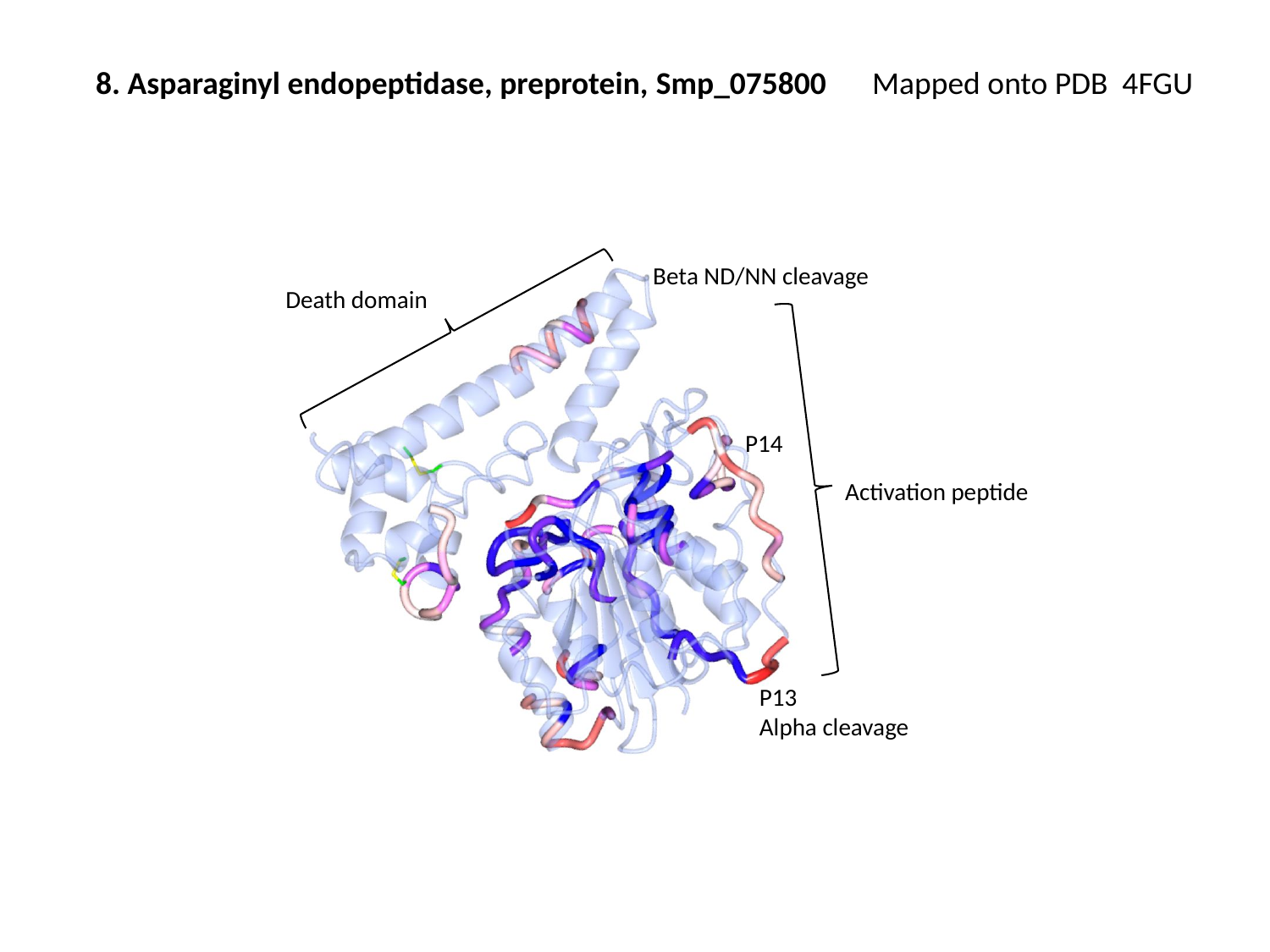

8. Asparaginyl endopeptidase, preprotein, Smp_075800
Mapped onto PDB 4FGU
Beta ND/NN cleavage
Death domain
Activation peptide
P13
Alpha cleavage
P14

## Slide 9
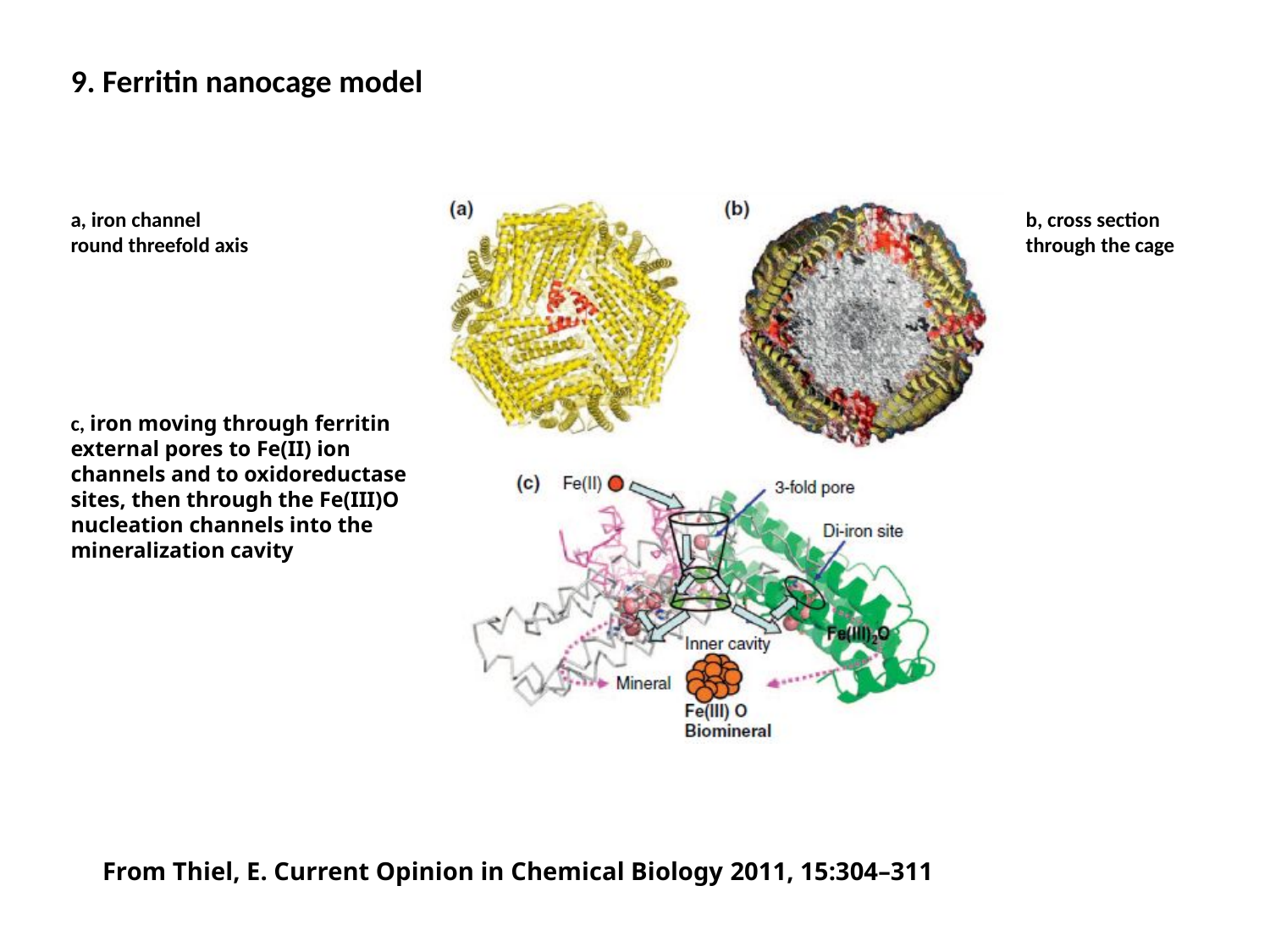

9. Ferritin nanocage model
a, iron channel round threefold axis
b, cross section through the cage
c, iron moving through ferritin external pores to Fe(II) ion channels and to oxidoreductase sites, then through the Fe(III)O nucleation channels into the mineralization cavity
From Thiel, E. Current Opinion in Chemical Biology 2011, 15:304–311

## Slide 10
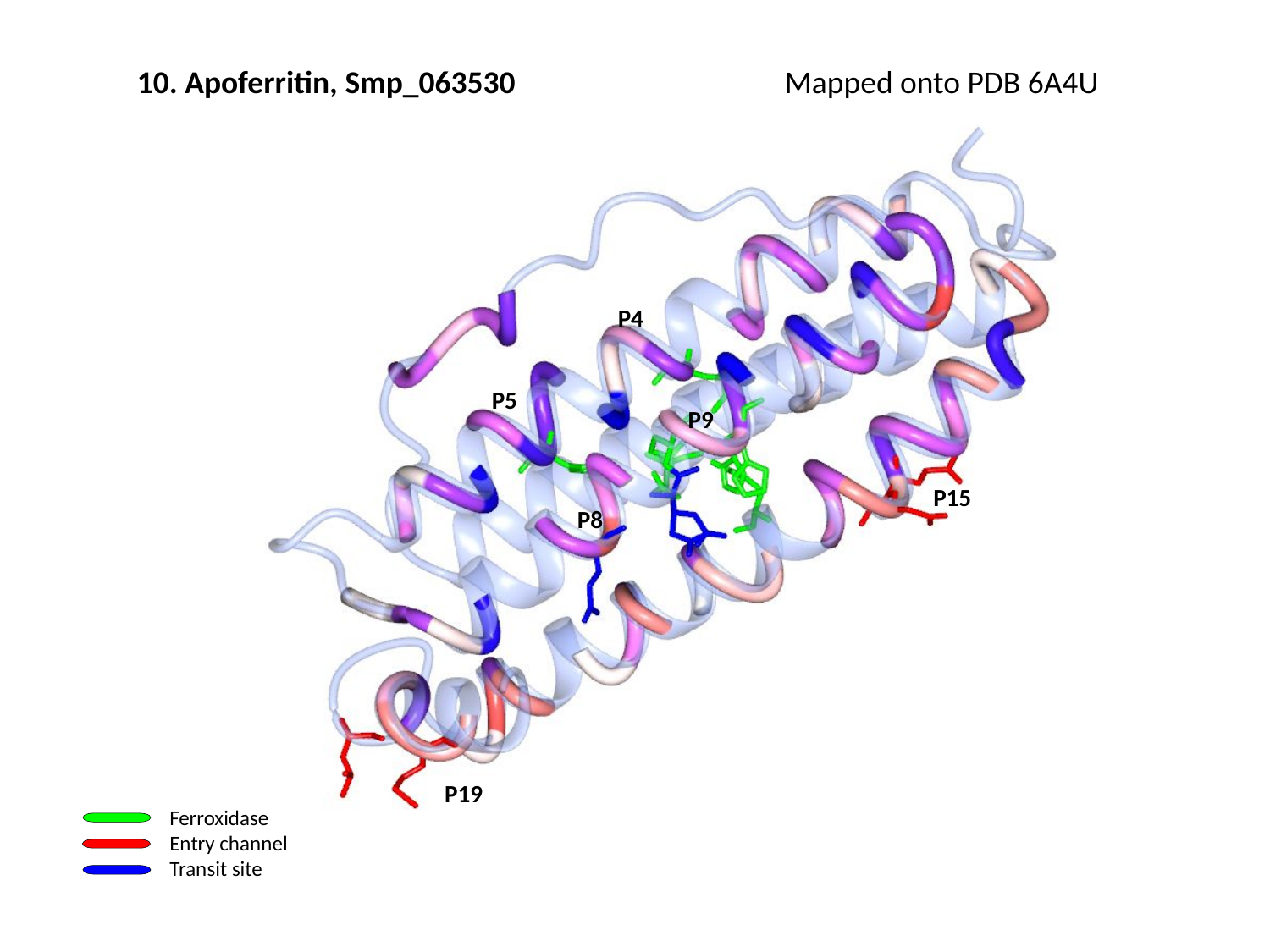

10. Apoferritin, Smp_063530
Mapped onto PDB 6A4U
P4
P5
P9
P15
P8
P19
Ferroxidase
Entry channel
Transit site

## Slide 11
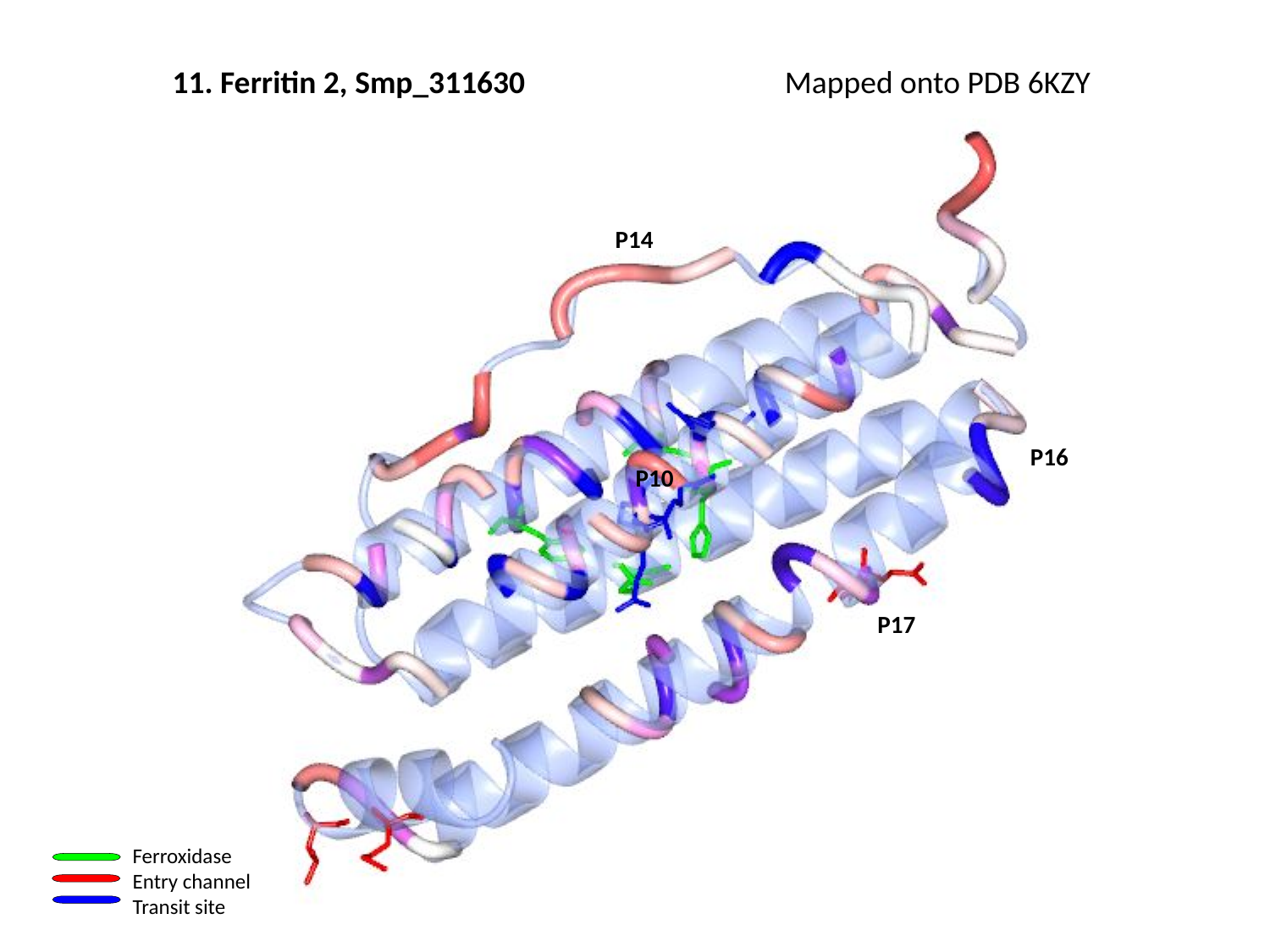

11. Ferritin 2, Smp_311630
Mapped onto PDB 6KZY
P14
P16
P16
P10
P17
Ferroxidase
Entry channel
Transit site

## Slide 12
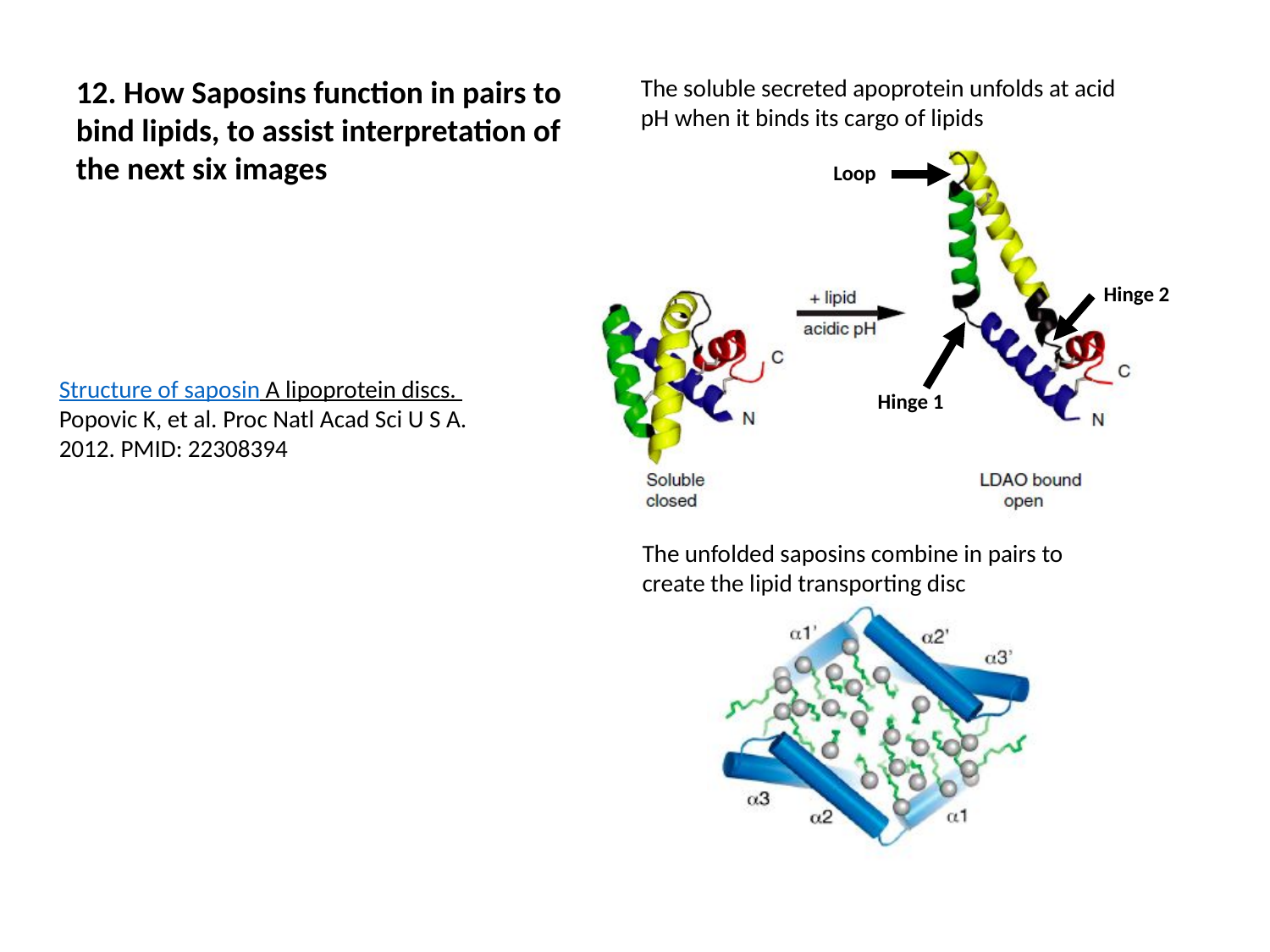

12. How Saposins function in pairs to bind lipids, to assist interpretation of the next six images
The soluble secreted apoprotein unfolds at acid pH when it binds its cargo of lipids
Loop
Hinge 2
Hinge 1
The unfolded saposins combine in pairs to create the lipid transporting disc
Structure of saposin A lipoprotein discs. Popovic K, et al. Proc Natl Acad Sci U S A. 2012. PMID: 22308394

## Slide 13
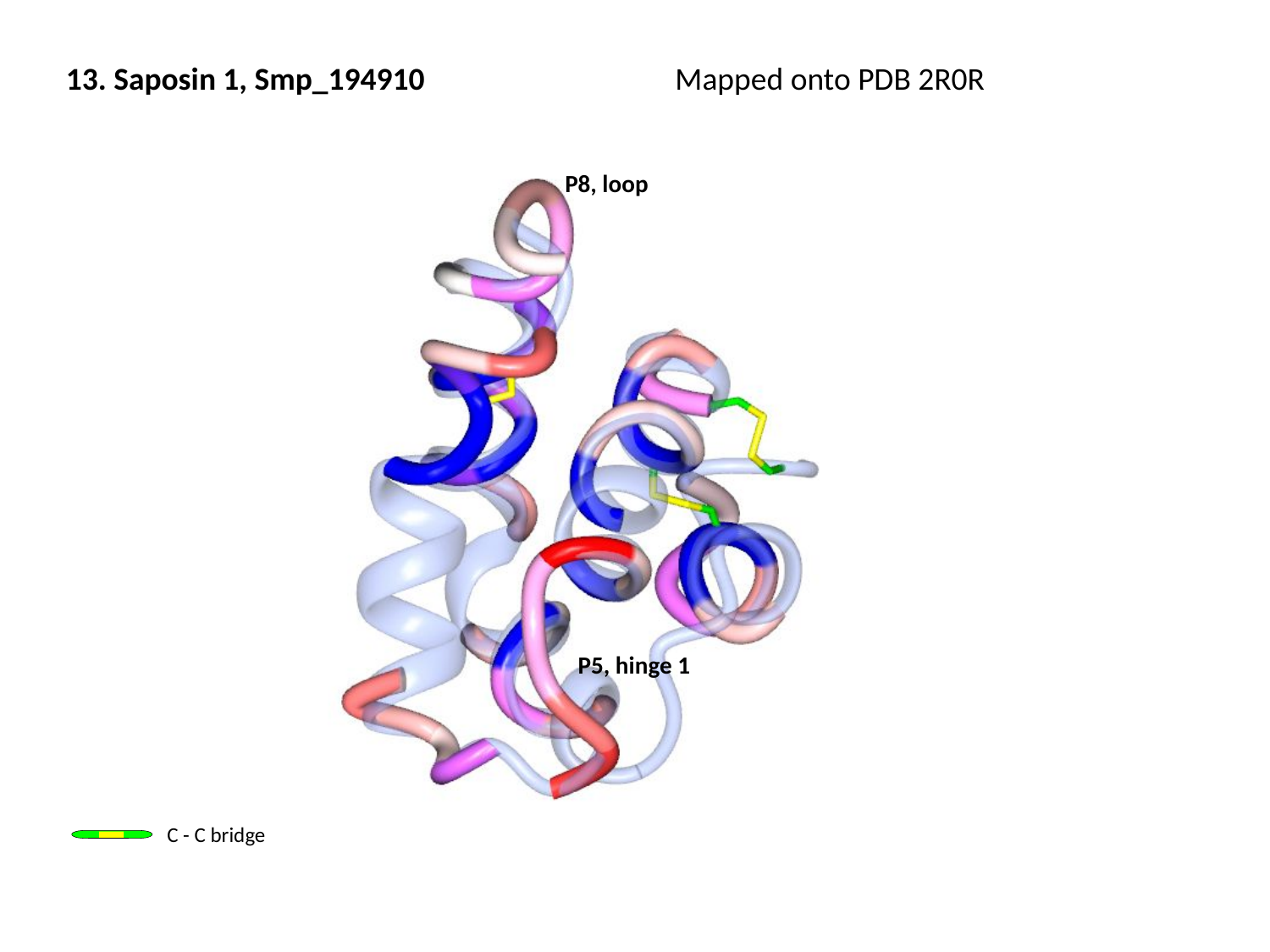

13. Saposin 1, Smp_194910
Mapped onto PDB 2R0R
P8, loop
P5, hinge 1
C - C bridge

## Slide 14
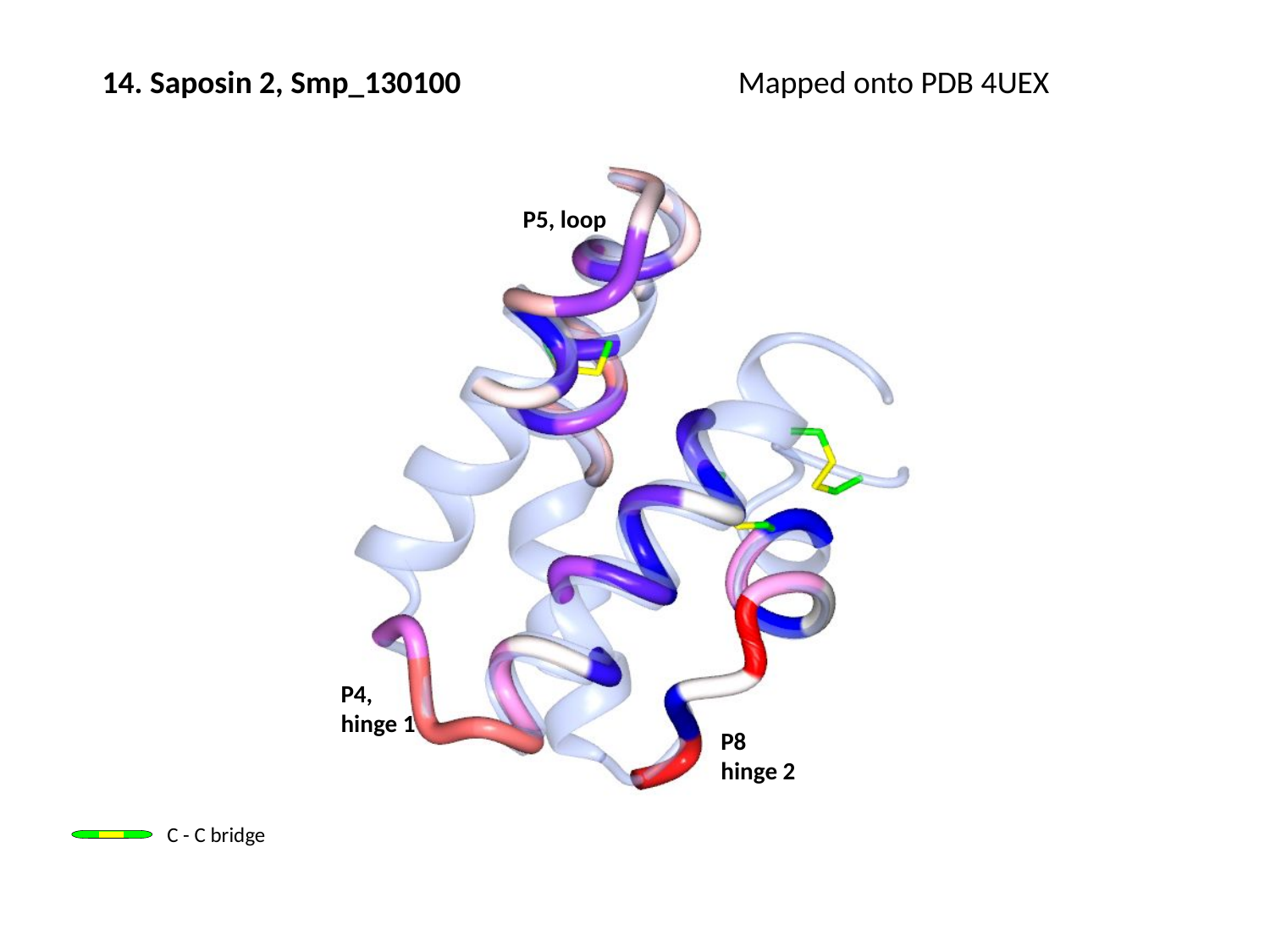

14. Saposin 2, Smp_130100
Mapped onto PDB 4UEX
P5, loop
P4,
hinge 1
P8 hinge 2
C - C bridge

## Slide 15
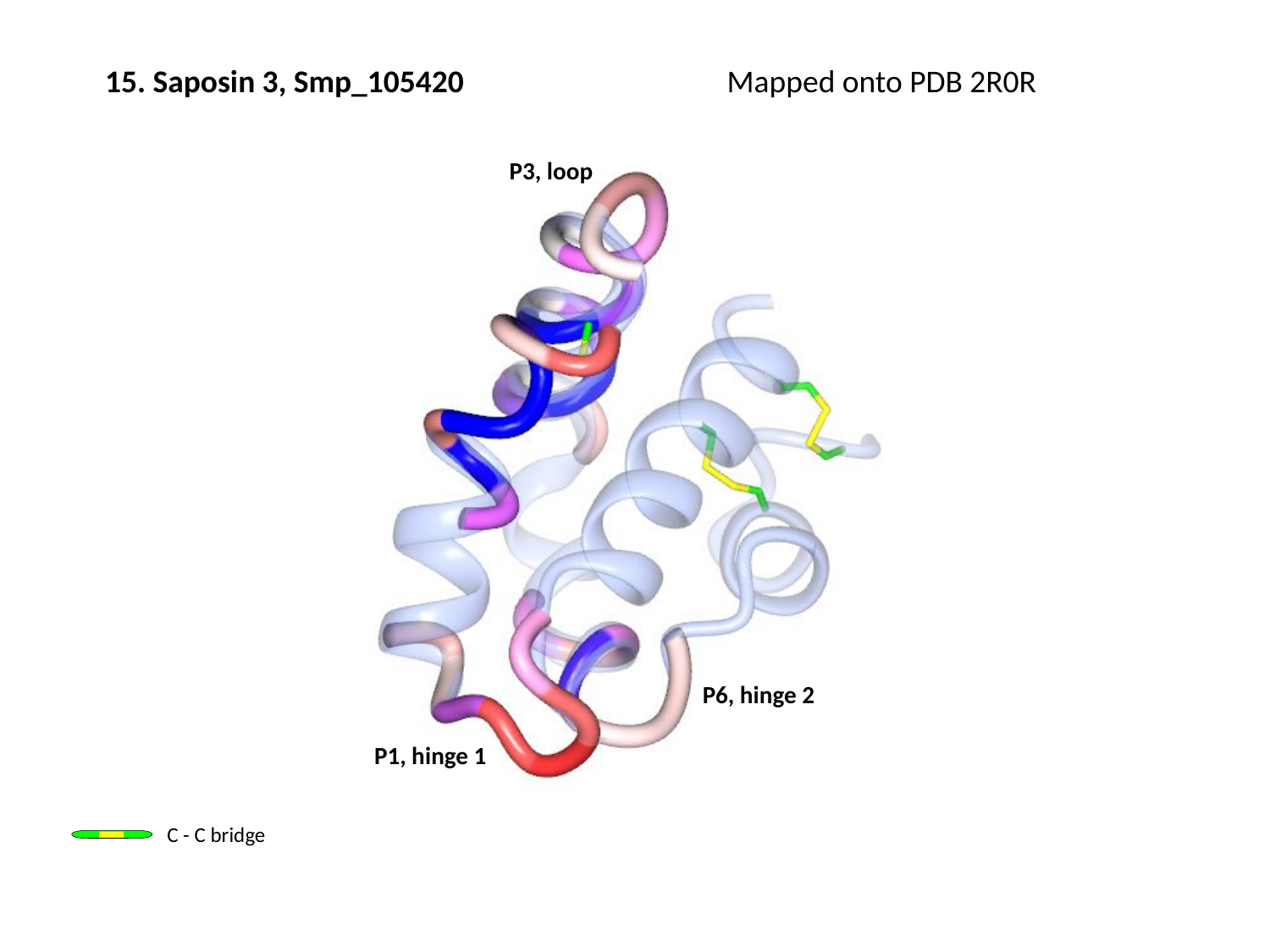

15. Saposin 3, Smp_105420
Mapped onto PDB 2R0R
P3, loop
P6, hinge 2
P1, hinge 1
C - C bridge

## Slide 16
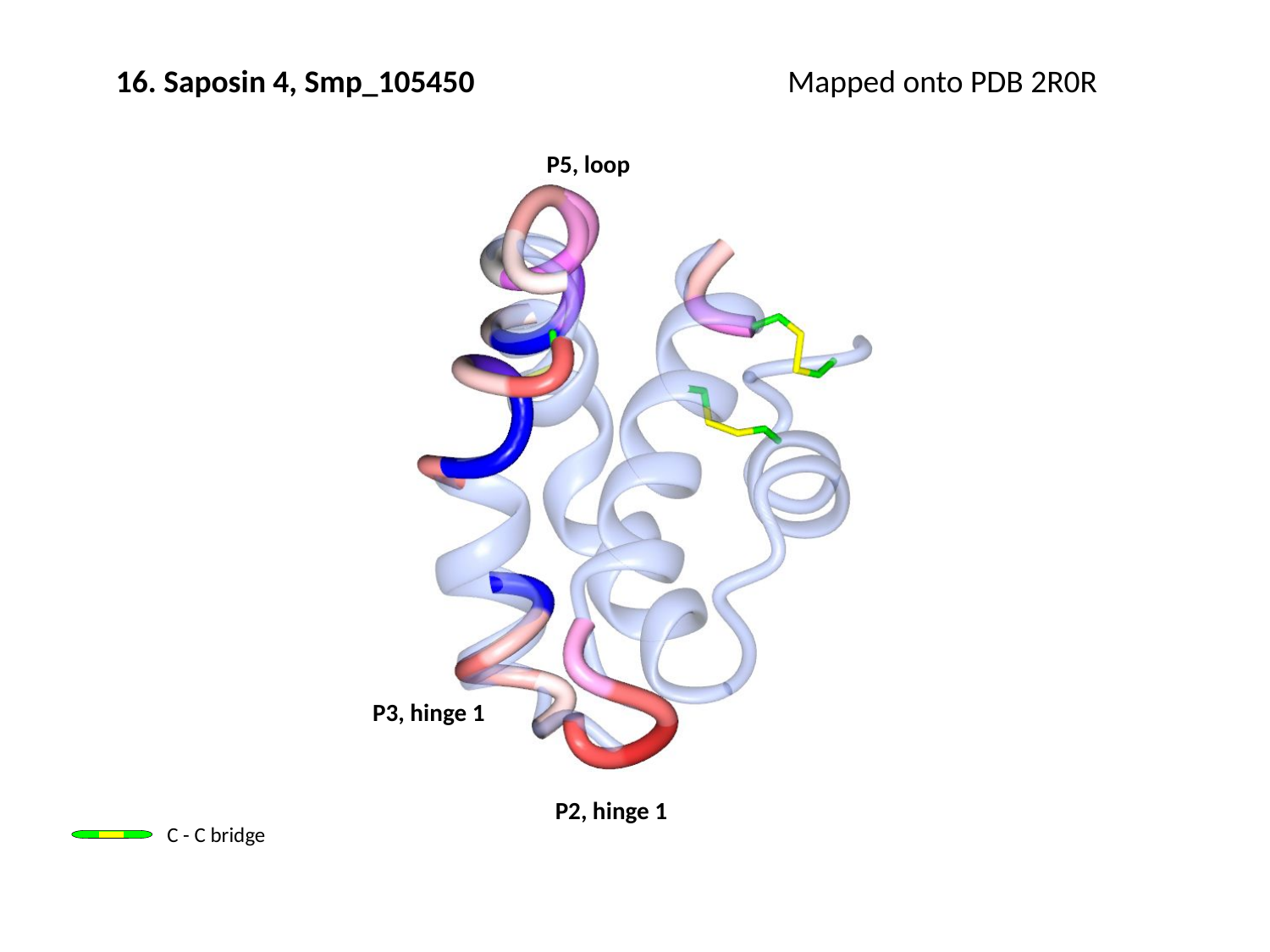

16. Saposin 4, Smp_105450
Mapped onto PDB 2R0R
P5, loop
P3, hinge 1
P2, hinge 1
C - C bridge

## Slide 17
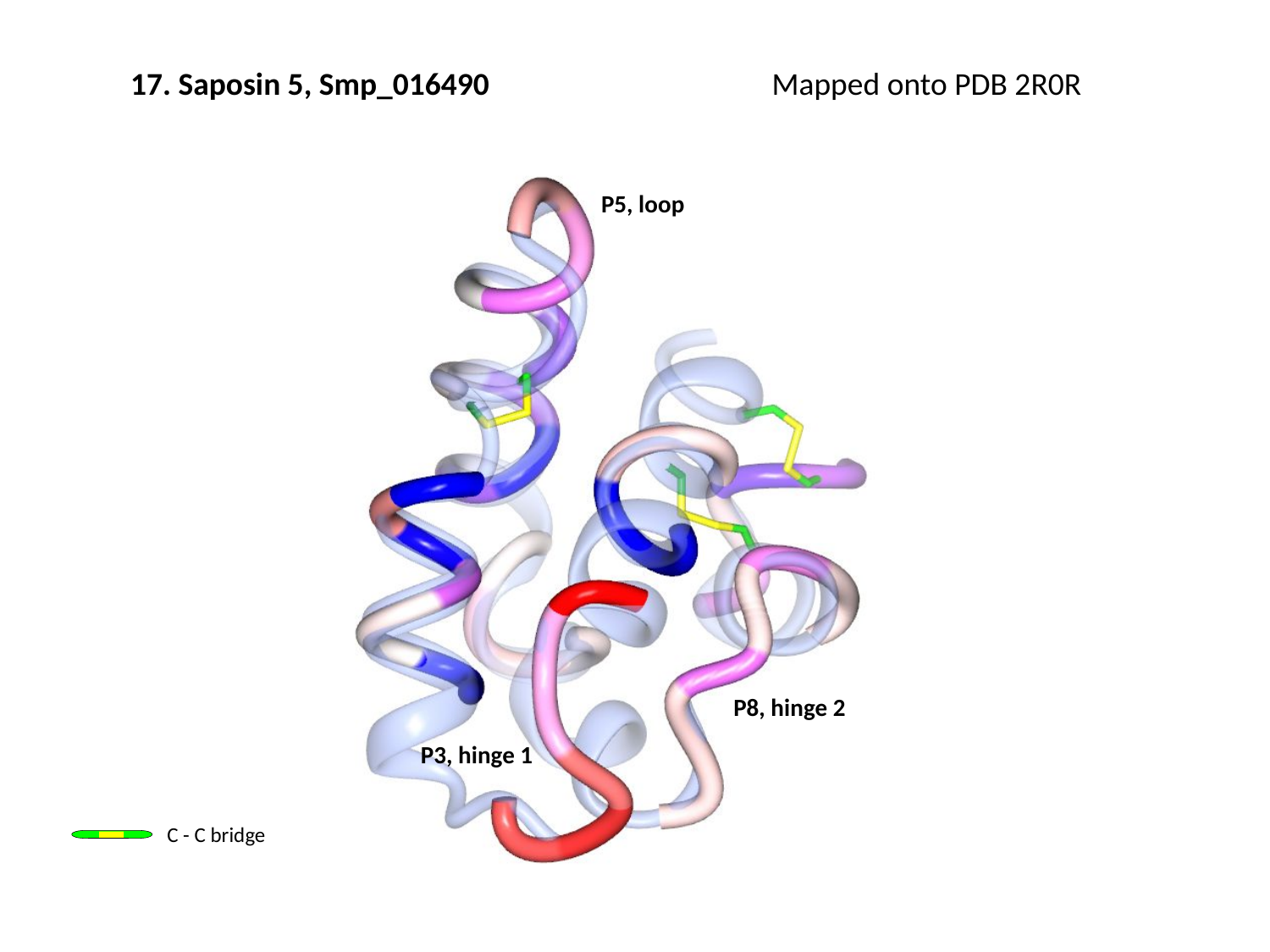

17. Saposin 5, Smp_016490
Mapped onto PDB 2R0R
P5, loop
P8, hinge 2
P3, hinge 1
C - C bridge

## Slide 18
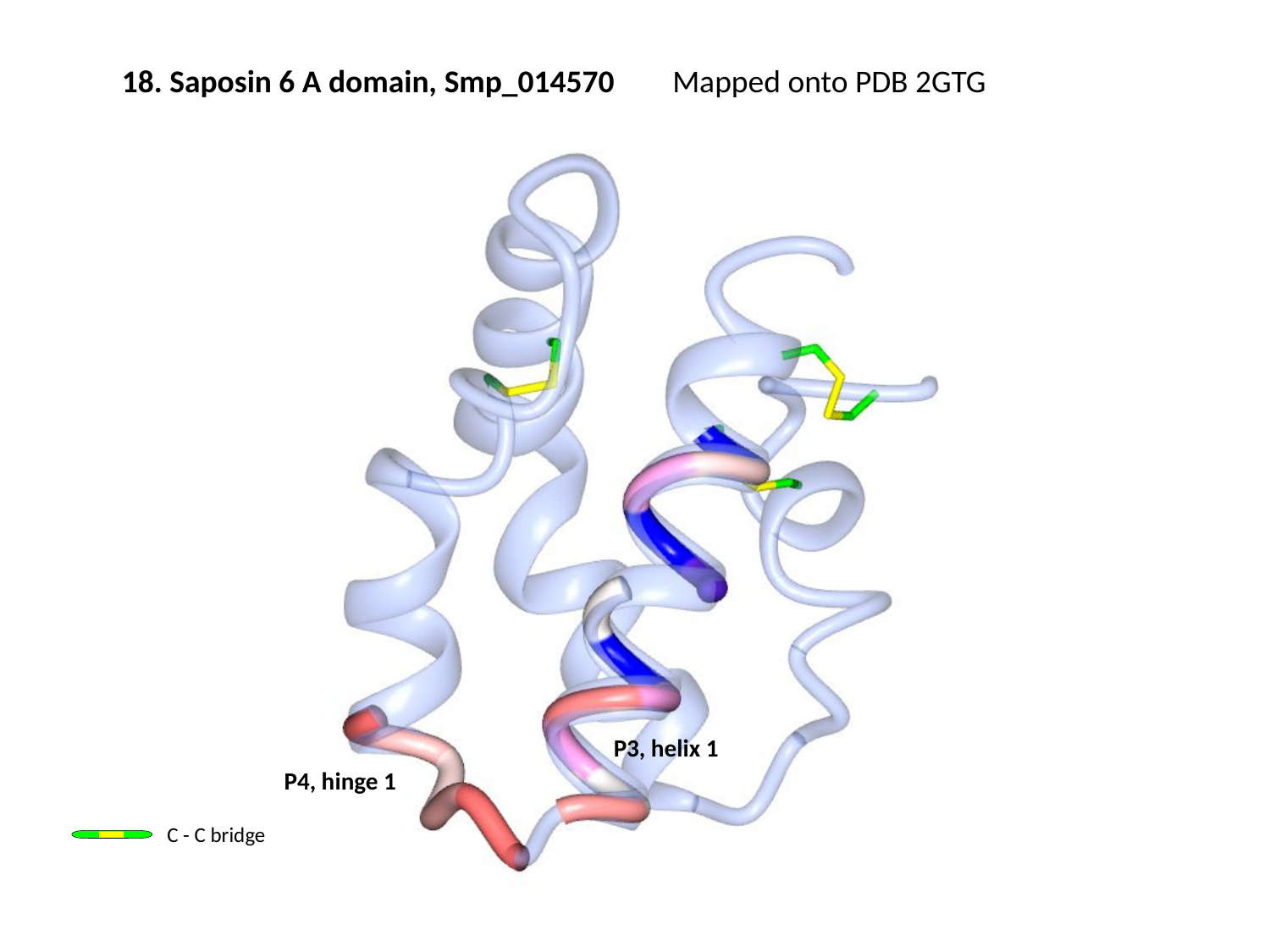

18. Saposin 6 A domain, Smp_014570
Mapped onto PDB 2GTG
P4, hinge 1
P3, helix 1
C - C bridge

## Slide 19
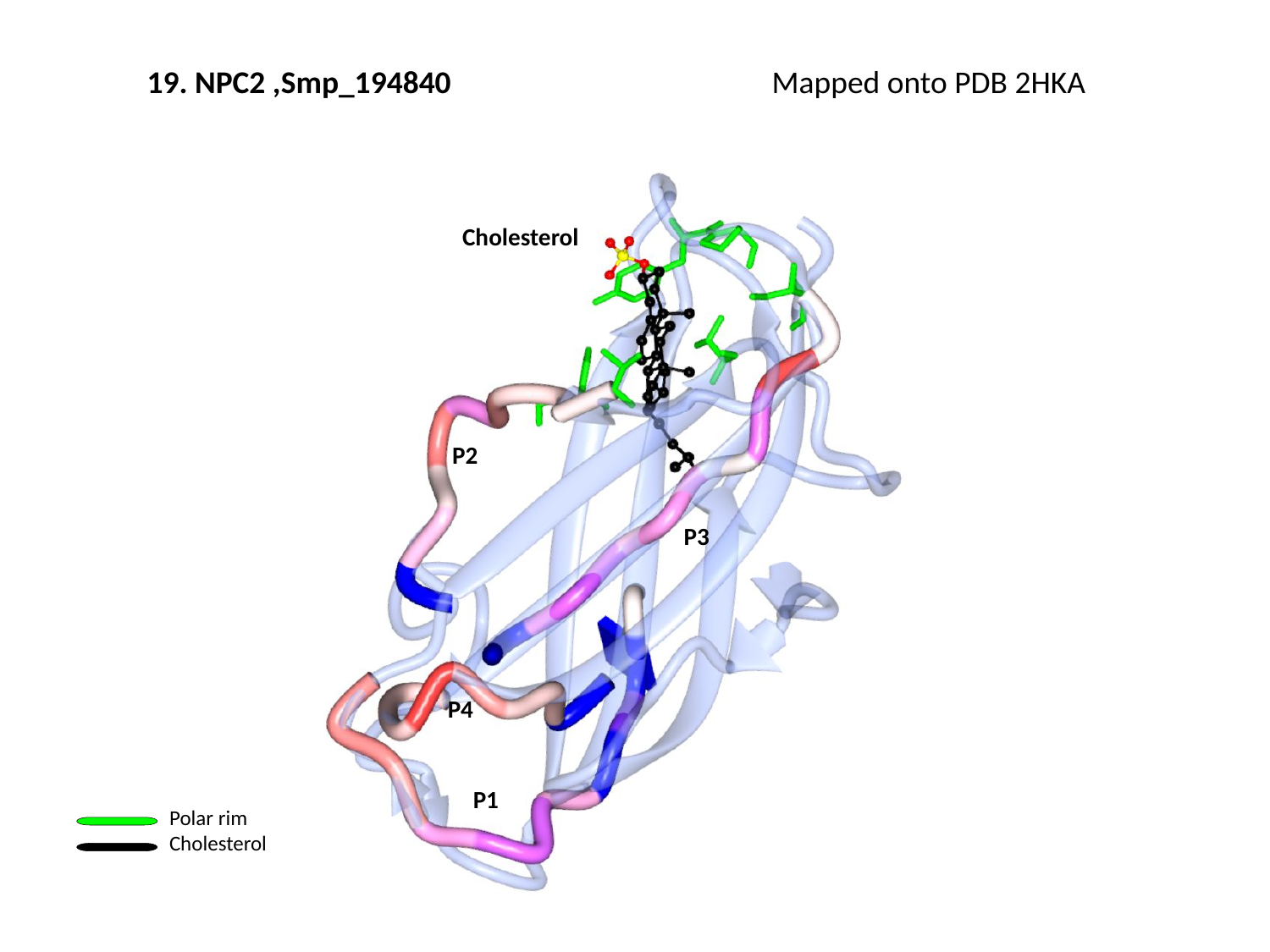

19. NPC2 ,Smp_194840
Mapped onto PDB 2HKA
Cholesterol
P2
P3
P4
P1
Polar rim
Cholesterol

## Slide 20
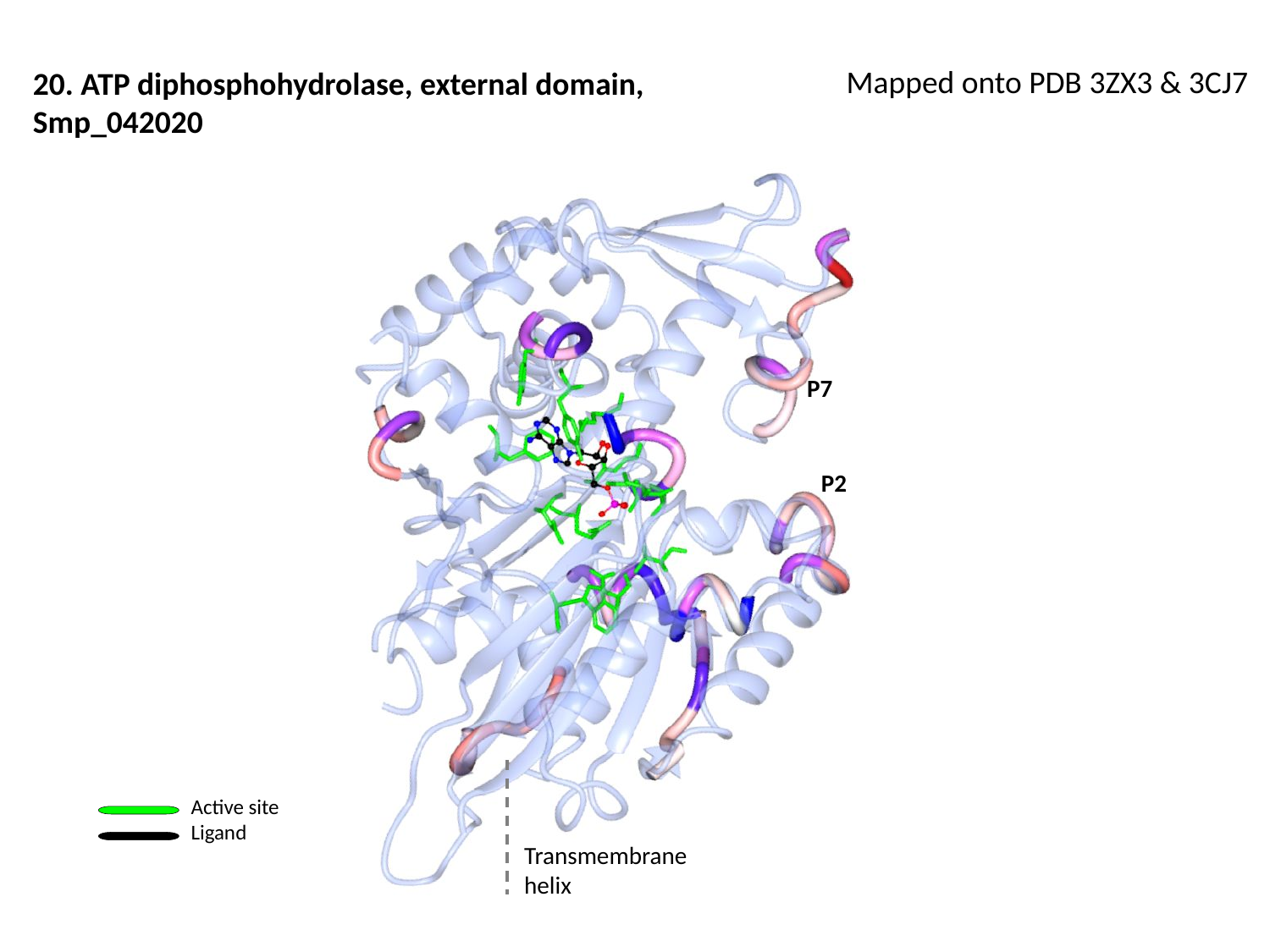

Mapped onto PDB 3ZX3 & 3CJ7
20. ATP diphosphohydrolase, external domain, Smp_042020
P7
P2
Active site
Ligand
Transmembrane
helix

## Slide 21
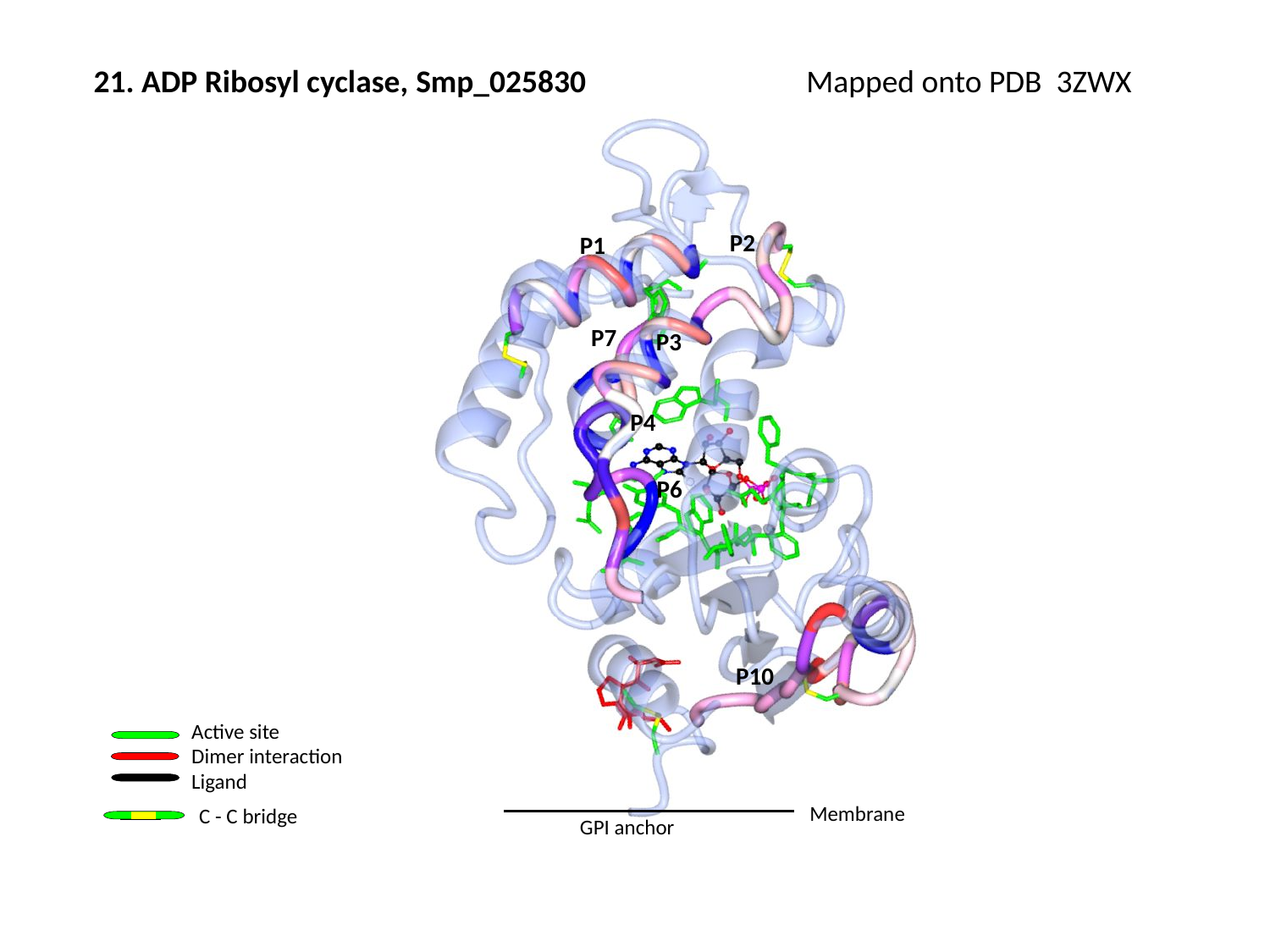

21. ADP Ribosyl cyclase, Smp_025830
Mapped onto PDB 3ZWX
P10
P6
P4
P7
P3
P1
P2
Active site
Dimer interaction
Ligand
C - C bridge
Membrane
GPI anchor

## Slide 22
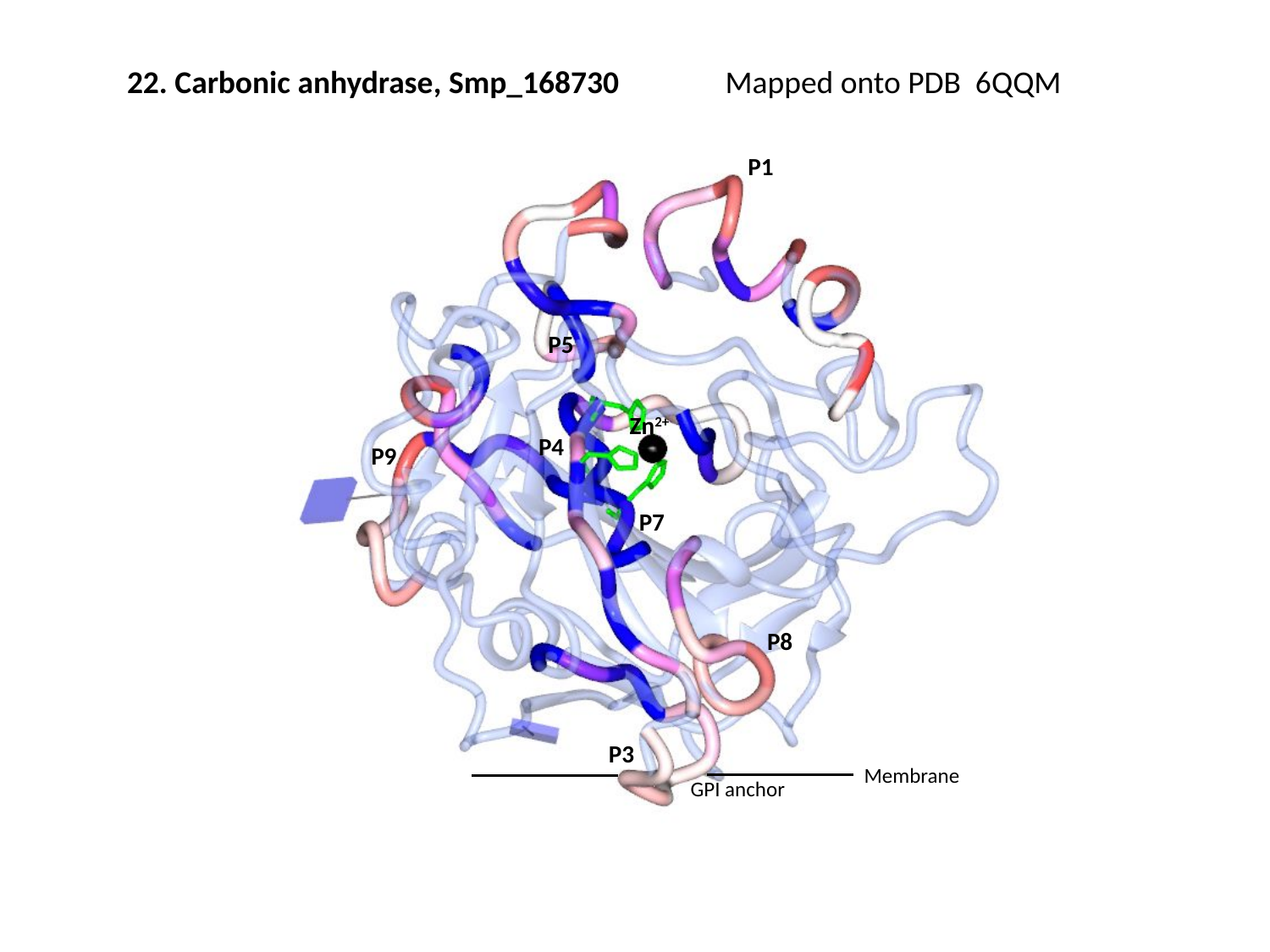

22. Carbonic anhydrase, Smp_168730
Mapped onto PDB 6QQM
P1
P5
Zn2+
P4
P9
P7
P8
P3
Membrane
GPI anchor

## Slide 23
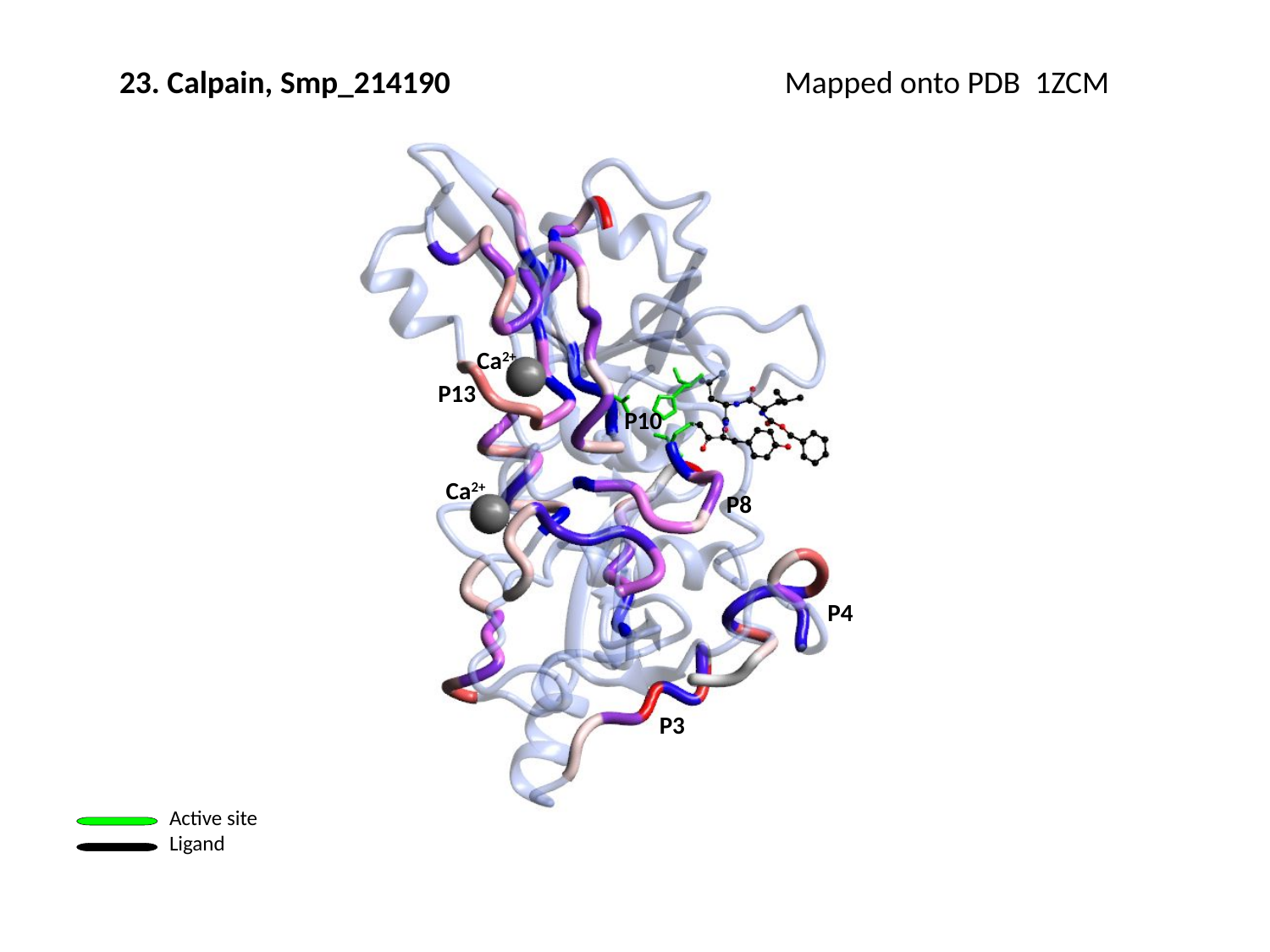

23. Calpain, Smp_214190
Mapped onto PDB 1ZCM
Ca2+
P13
P10
P8
P4
P3
Ca2+
Active site
Ligand

## Slide 24
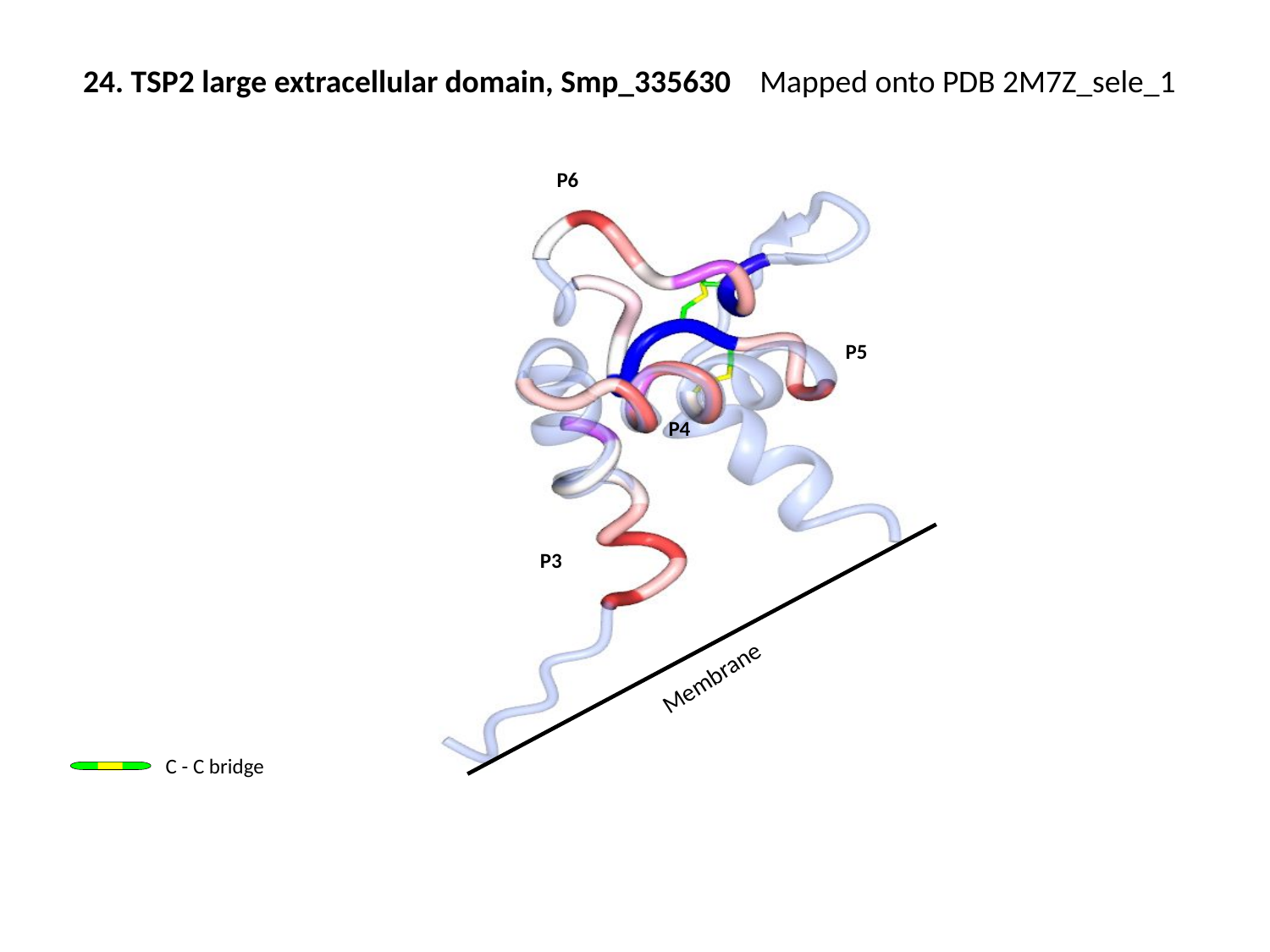

24. TSP2 large extracellular domain, Smp_335630
Mapped onto PDB 2M7Z_sele_1
P6
P3
P5
P4
Membrane
C - C bridge

## Slide 25
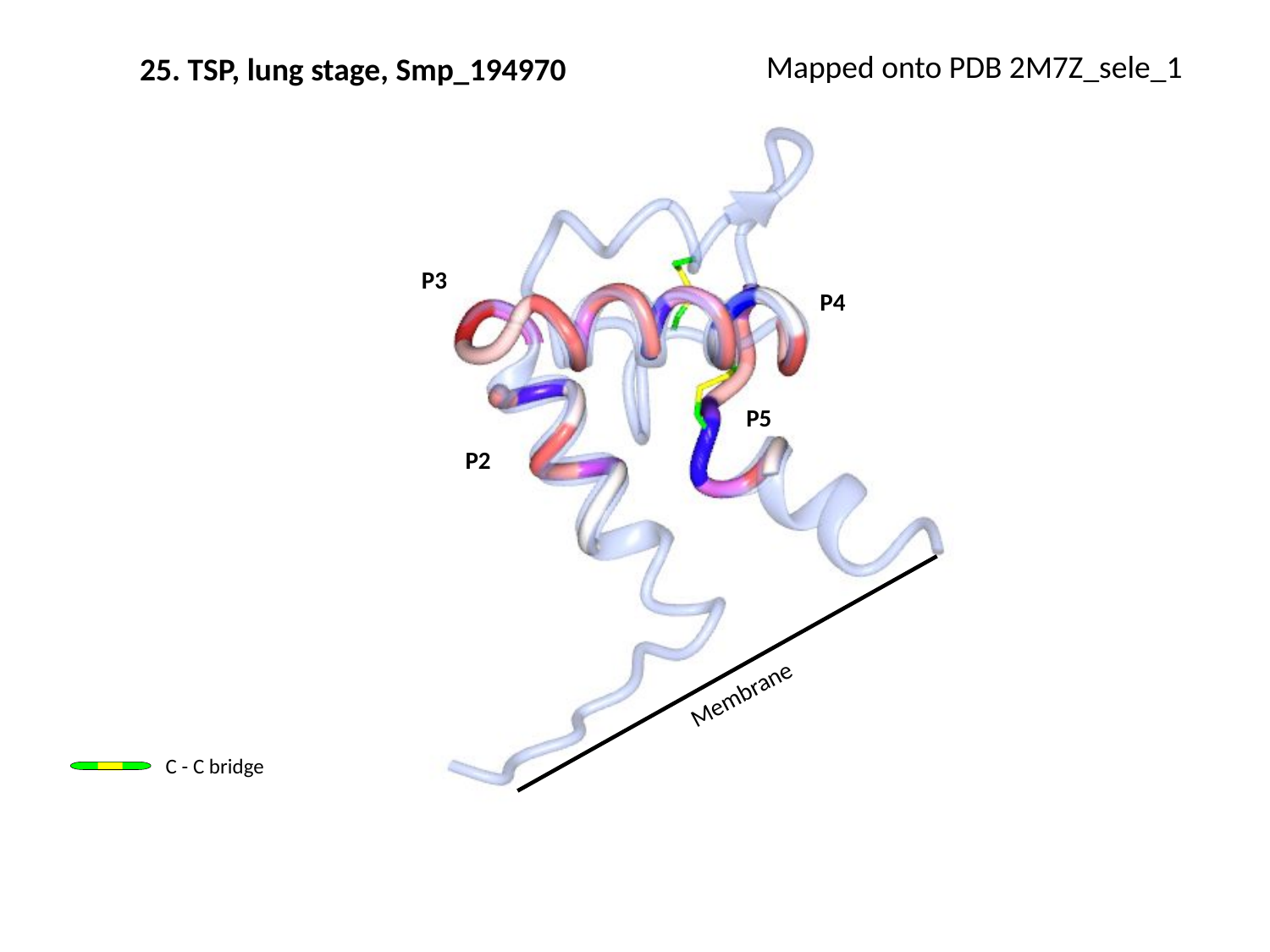

25. TSP, lung stage, Smp_194970
Mapped onto PDB 2M7Z_sele_1
P3
P4
P5
P2
Membrane
C - C bridge

## Slide 26
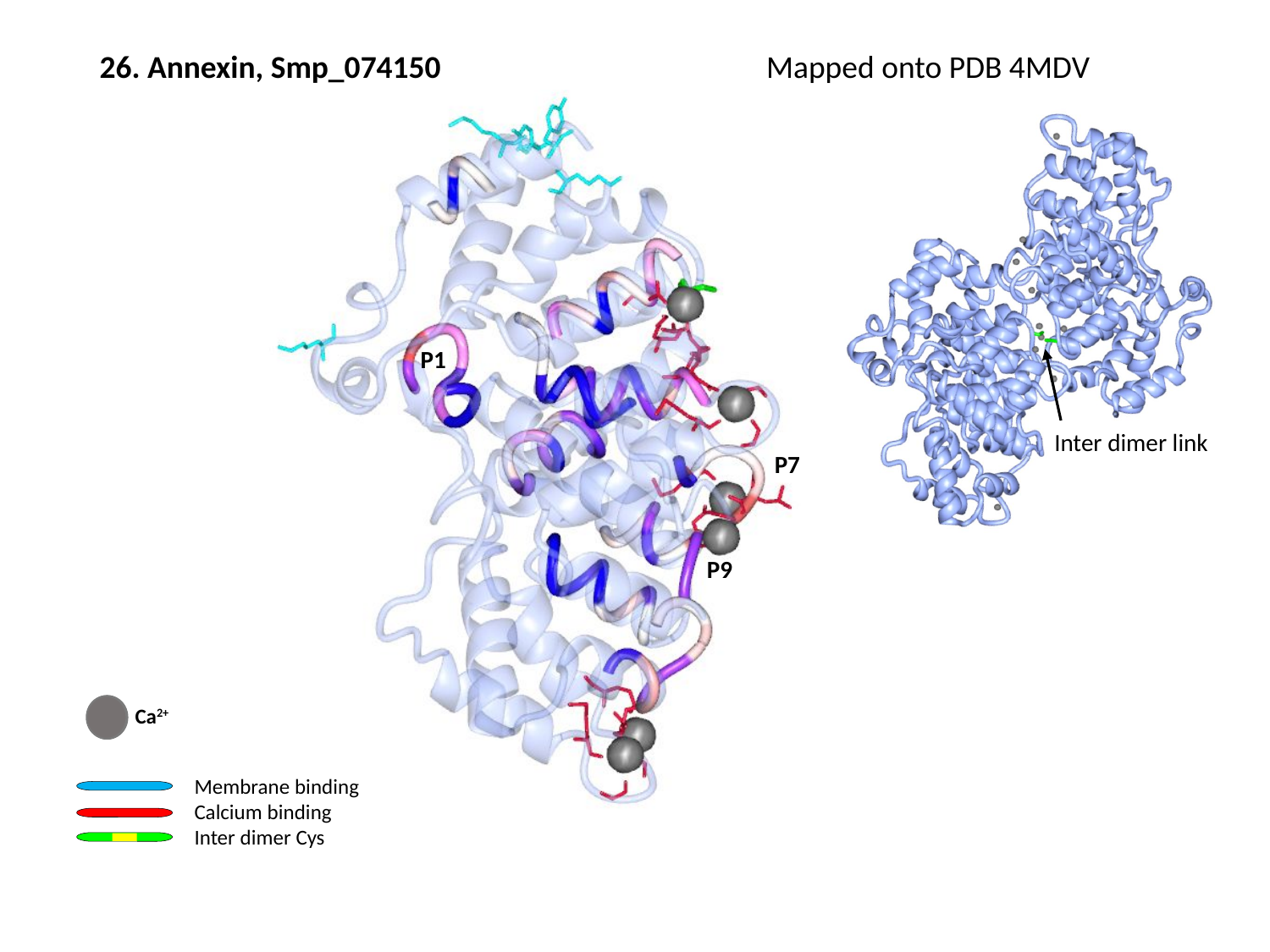

26. Annexin, Smp_074150
Mapped onto PDB 4MDV
P1
P7
P9
Inter dimer link
Ca2+
Membrane binding
Calcium binding
Inter dimer Cys

## Slide 27
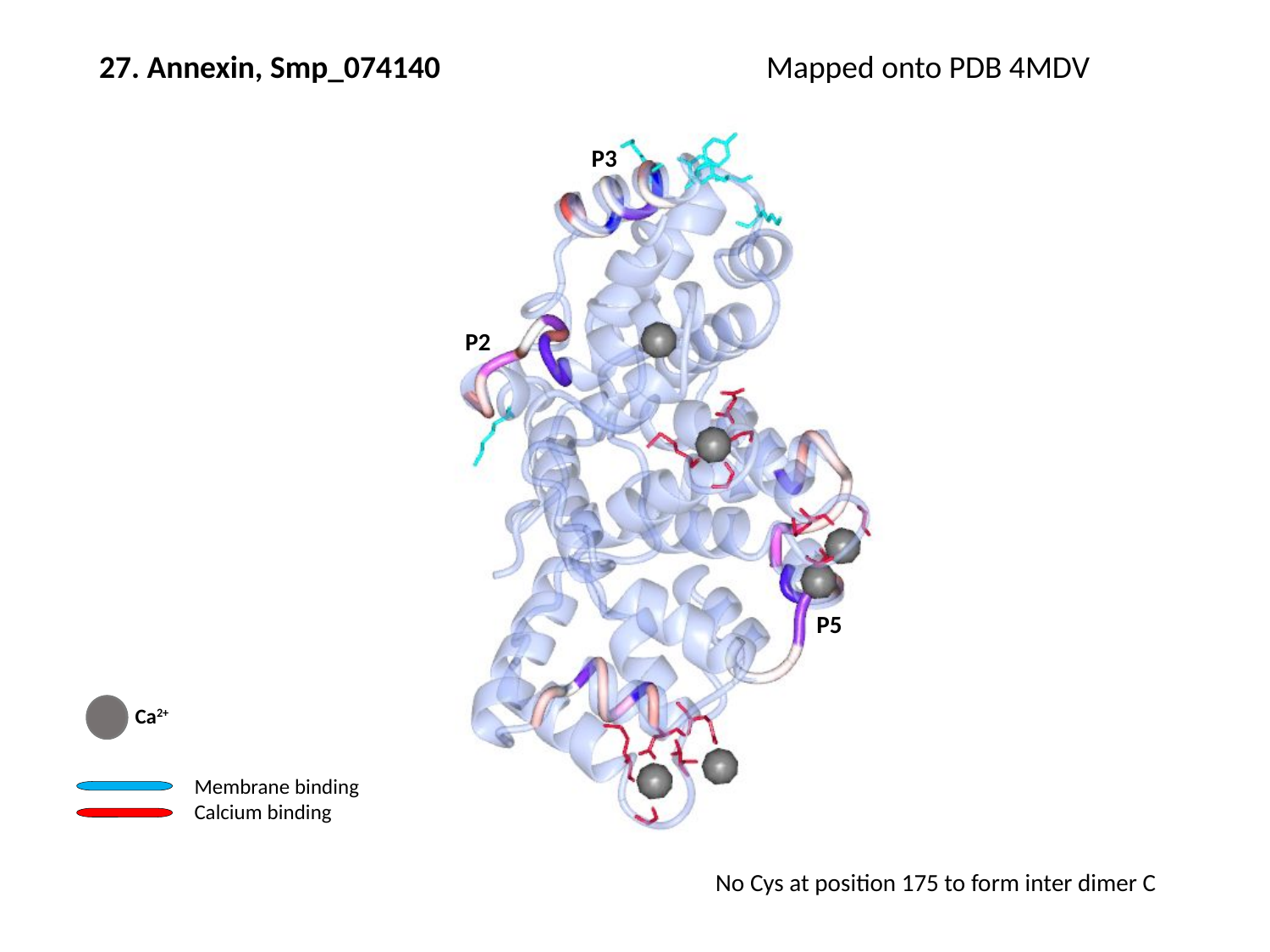

27. Annexin, Smp_074140
Mapped onto PDB 4MDV
P3
P2
P5
Ca2+
Membrane binding
Calcium binding
No Cys at position 175 to form inter dimer C

## Slide 28
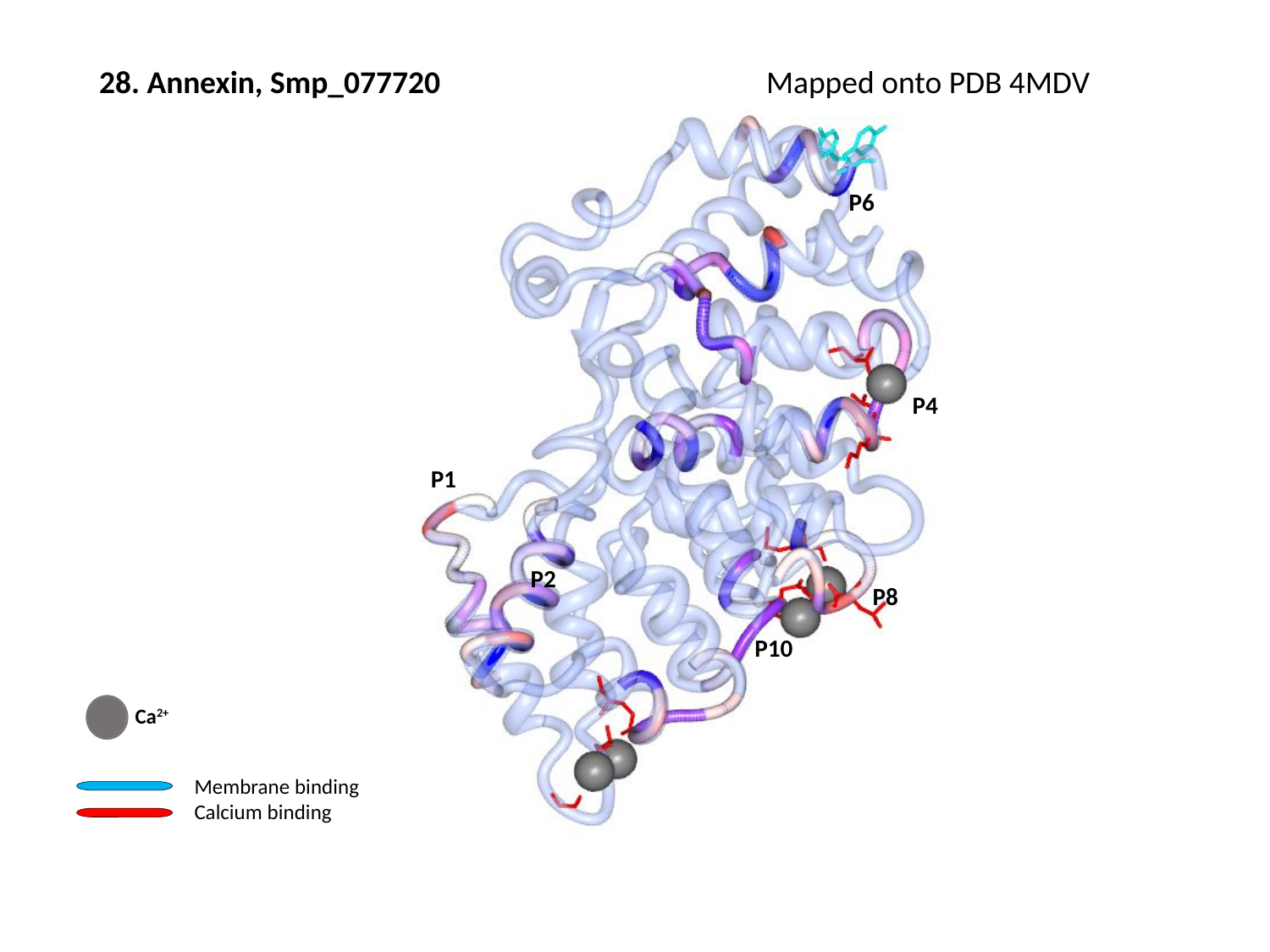

28. Annexin, Smp_077720
Mapped onto PDB 4MDV
P6
P4
P1
P2
P8
P10
Ca2+
Membrane binding
Calcium binding

## Slide 29
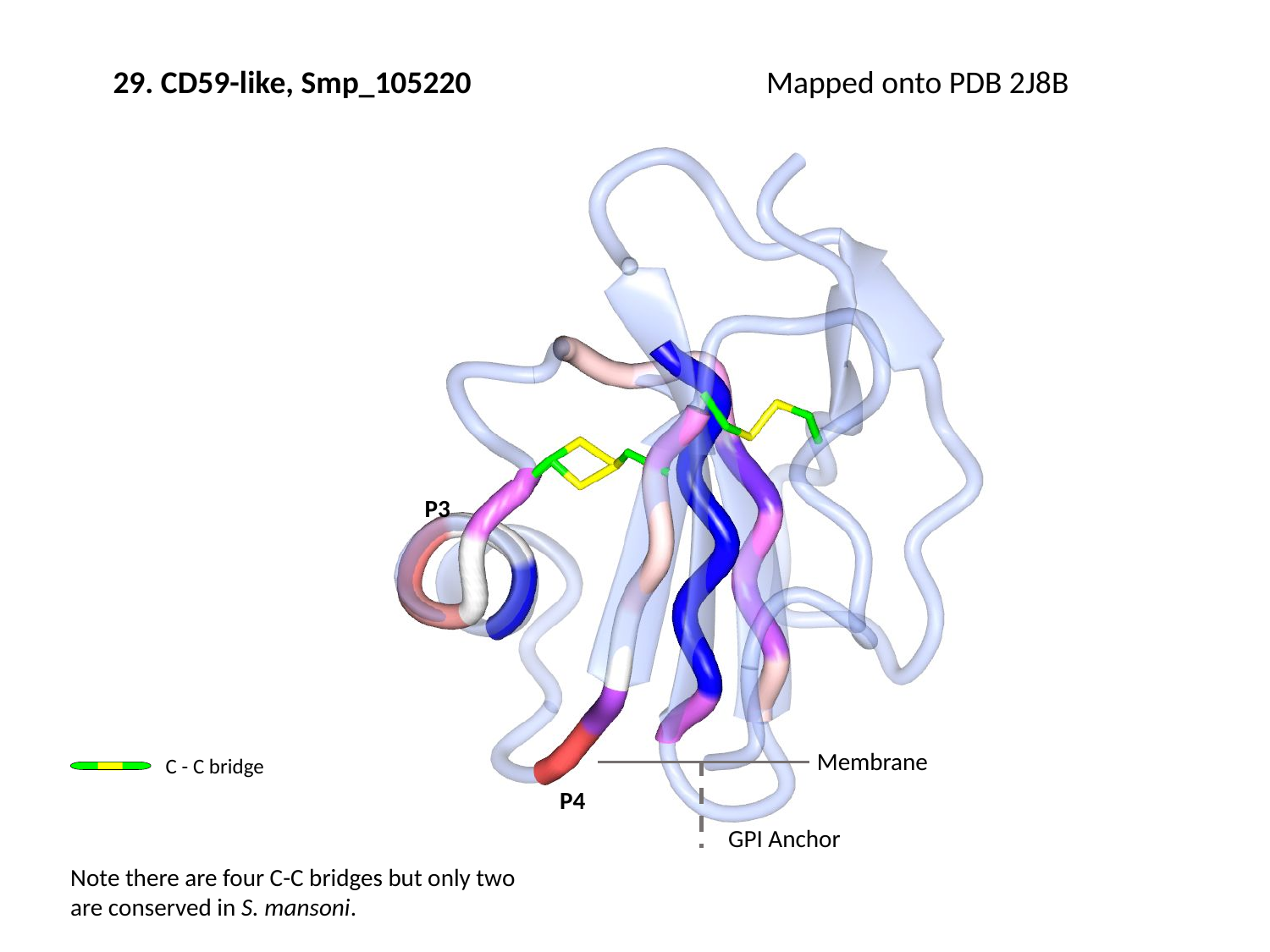

29. CD59-like, Smp_105220
Mapped onto PDB 2J8B
P3
P4
GPI Anchor
Membrane
C - C bridge
Note there are four C-C bridges but only two are conserved in S. mansoni.

## Slide 30
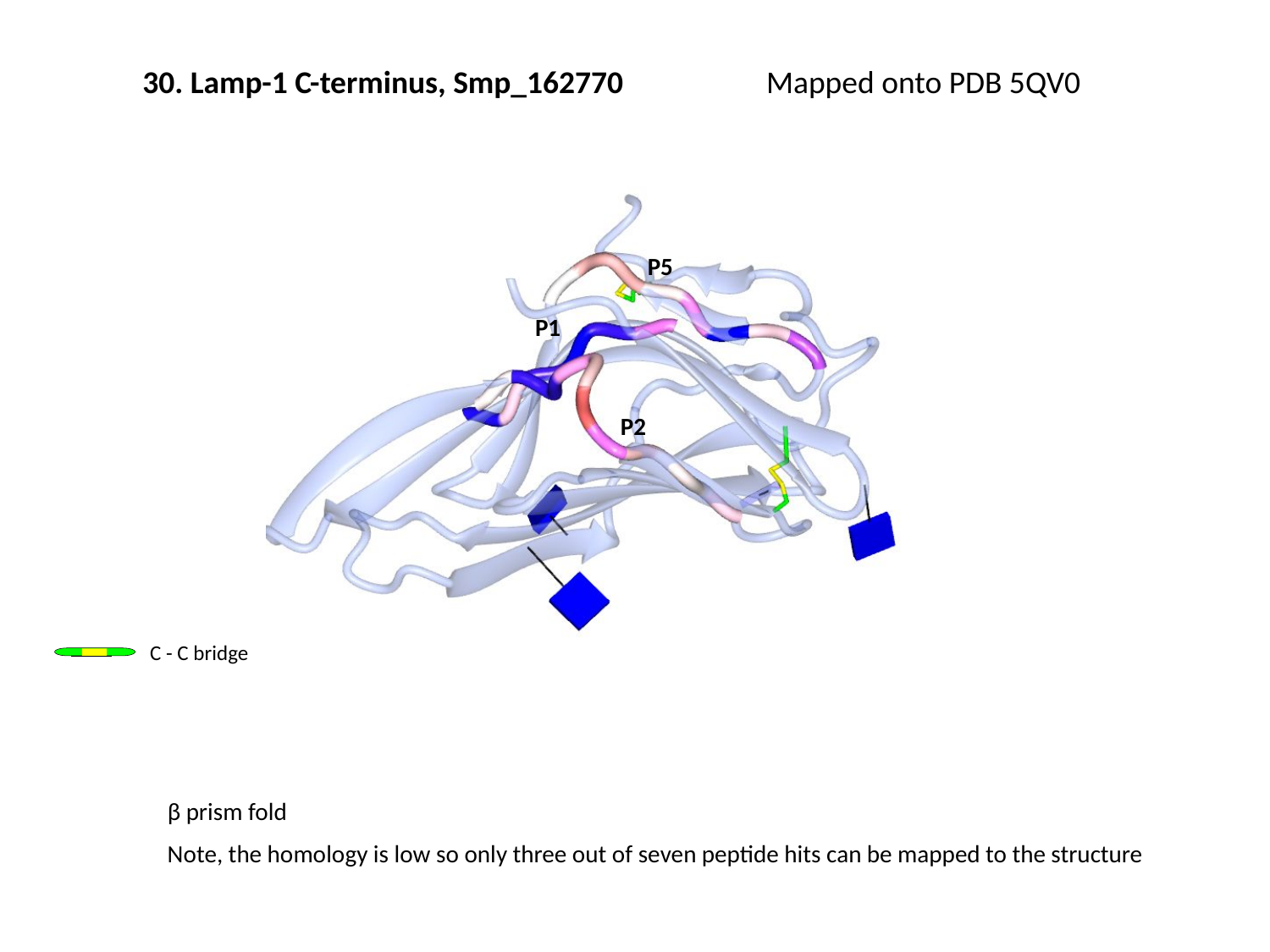

30. Lamp-1 C-terminus, Smp_162770
Mapped onto PDB 5QV0
P5
P1
P2
C - C bridge
β prism fold
Note, the homology is low so only three out of seven peptide hits can be mapped to the structure

## Slide 31
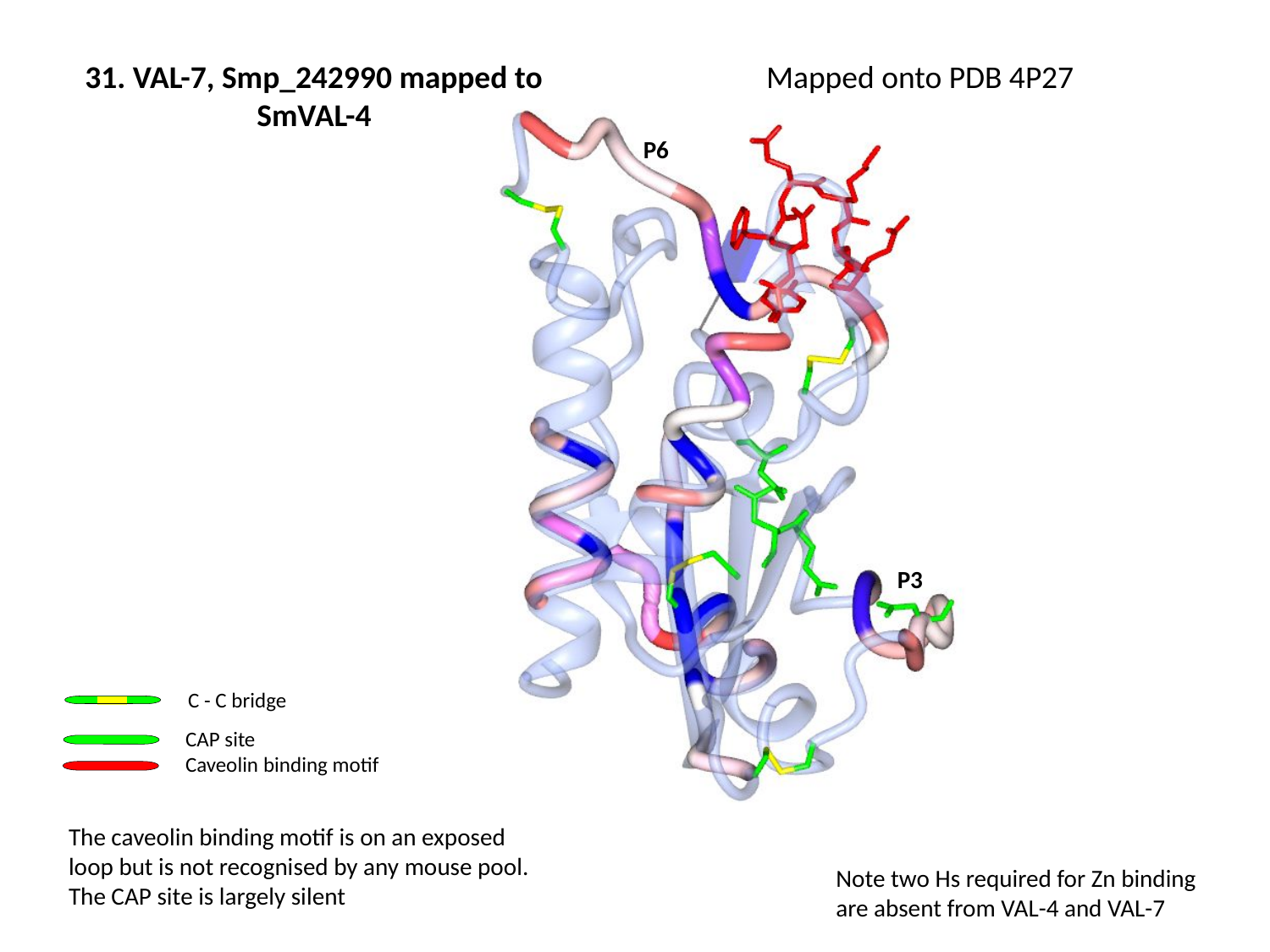

31. VAL-7, Smp_242990 mapped to SmVAL-4
Mapped onto PDB 4P27
P6
P3
 C - C bridge
 CAP site
 Caveolin binding motif
The caveolin binding motif is on an exposed loop but is not recognised by any mouse pool. The CAP site is largely silent
Note two Hs required for Zn binding are absent from VAL-4 and VAL-7
