# Supplementary material for: Epitope Mapping of Exposed Tegument and Alimentary Tract Proteins Identifies Putative Antigenic Targets of the Attenuated Schistosome Vaccine
Source: Front Immunol. 2021 Mar 3;11:624613. doi: 10.3389/fimmu.2020.624613 (PMC7982949; doi:10.3389/fimmu.2020.624613)
Supplement: Supplementary file 1 [file DataSheet_1.zip › Supplementary Material/Supplementary Figure 5.pdf]

Supplementary Figure 5. Peptides mapped to MEG conserved regions, selected REACTIVE peptides.

MEG-4

```
SmMEG-4.1  GTSDKPKSIADIFLINKPKVPLWIVNPLYYMVEKFVQIMGYLLEDDDTLELNLPKYYYDKSI
SmMEG-4.2  EEEQNPFHKLSEVLNSGSVVPLWLVNPIYYVLELFPRAISYYFN
             .::*    . .* .   ****:***:**::* * : :.* ::
```

MEG-8

```
SmMEG-8.1  PGKPESFLKRVGDGFFDLFSEQEFHPINHKSYLFNFWYLFRTSFLNLKNMKNLLLGS
SmMEG-8.2  EAKSLSLKERIMNKFNSIFGEEEYNPPKDSDFTERLWMLFKHCFLNFKNLAKIFST-
SmMEG-8.3  EAESLTFKEKIVELYKNWMNEKEFNPPKESEFYERFWELFKHCFLNSKQLTKILPF-
            .:  :: ::: : : . :.*:*::* :...:  .:* **: .*** *:: :::
```

MEG-3

```
SmMEG-3.1  AQETRDAERECKKHCEGNNEYVTRYCGGLCSSNTGPQTFYCYLGCSHNASTQDDFDKCLPKCNDRVQLT
SmMEG-3.2  ---ARETQQECVRHCGGHNEYVTRYCGGLCSGSTGPQTFYCYLGCSHNASNQNDFDKCLPKCNGSPQLT
SmMEG-3.3  ------AQQECEKNCKGDNEYVSPNCGILCSGTIGPQTFYCYLGCSHNATKQSEFDNCKTKCDGGVQLT
                 :::** ::* *.****:  ** ***.. ***************:.*.:**:*  **:.  ***

SmMEG-3.1  EENCRDDCGRVTSHHELCGDVCGGNHGGSFPLCLYNCDQEHPR------EYERGYDKCKTKCYAMEGR
SmMEG-3.2  ESSCQNDCGRVTTHPELCGIVCGGNVGDSFPLCLYNCDQGNG---------SGNFDECKTKCYEMAGR
SmMEG-3.3  KEACLSNCGLITTHPELCDAVCGGNDGGSFPICLYNCDQKHTDPRKDGADGSEDFDKCKTKCYKMAGQ
           :. * .:** :*:* ***. ***** *.***:******* :          . .:*:****** * *:
```
