# Supplementary material for: Epitope Mapping of Exposed Tegument and Alimentary Tract Proteins Identifies Putative Antigenic Targets of the Attenuated Schistosome Vaccine
Source: Front Immunol. 2021 Mar 3;11:624613. doi: 10.3389/fimmu.2020.624613 (PMC7982949; doi:10.3389/fimmu.2020.624613)

**Supplementary Figure 2.** Flow chart of the peptide array staining and scanning procedure. Courtesy of PEPperPRINT, Heidelberg, Germany, www.pepperprint.com, info@pepperprint.com

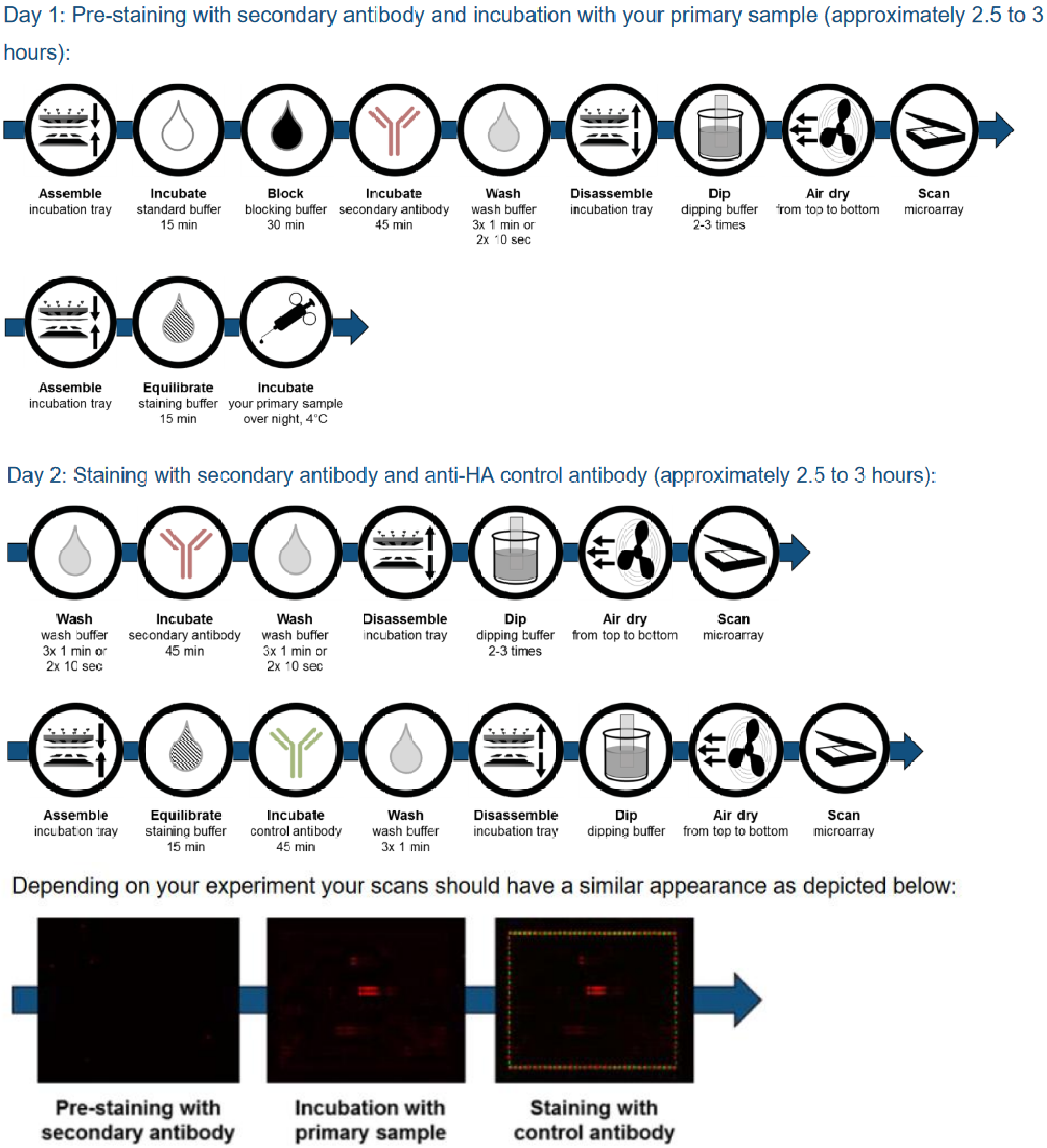

Supplement: Supplementary file 1 [file DataSheet_1.zip › Supplementary Material/Supplemetnary Figure 2.pdf]
