# Supplementary figures and images for: Epitope Mapping of Exposed Tegument and Alimentary Tract Proteins Identifies Putative Antigenic Targets of the Attenuated Schistosome Vaccine
Source: Front Immunol. 2021 Mar 3;11:624613. doi: 10.3389/fimmu.2020.624613 (PMC7982949; doi:10.3389/fimmu.2020.624613)

## Tegument

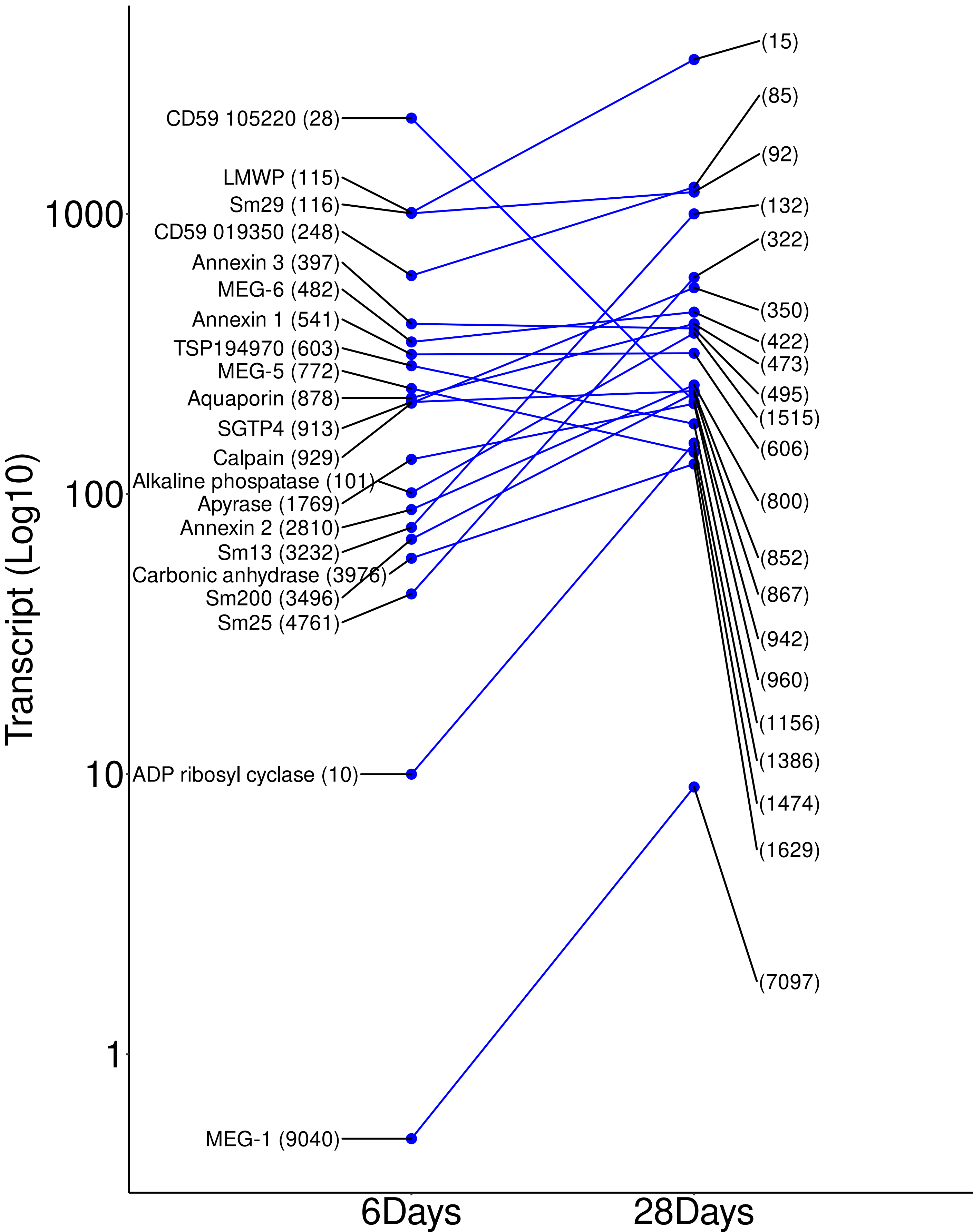

## Esophagus

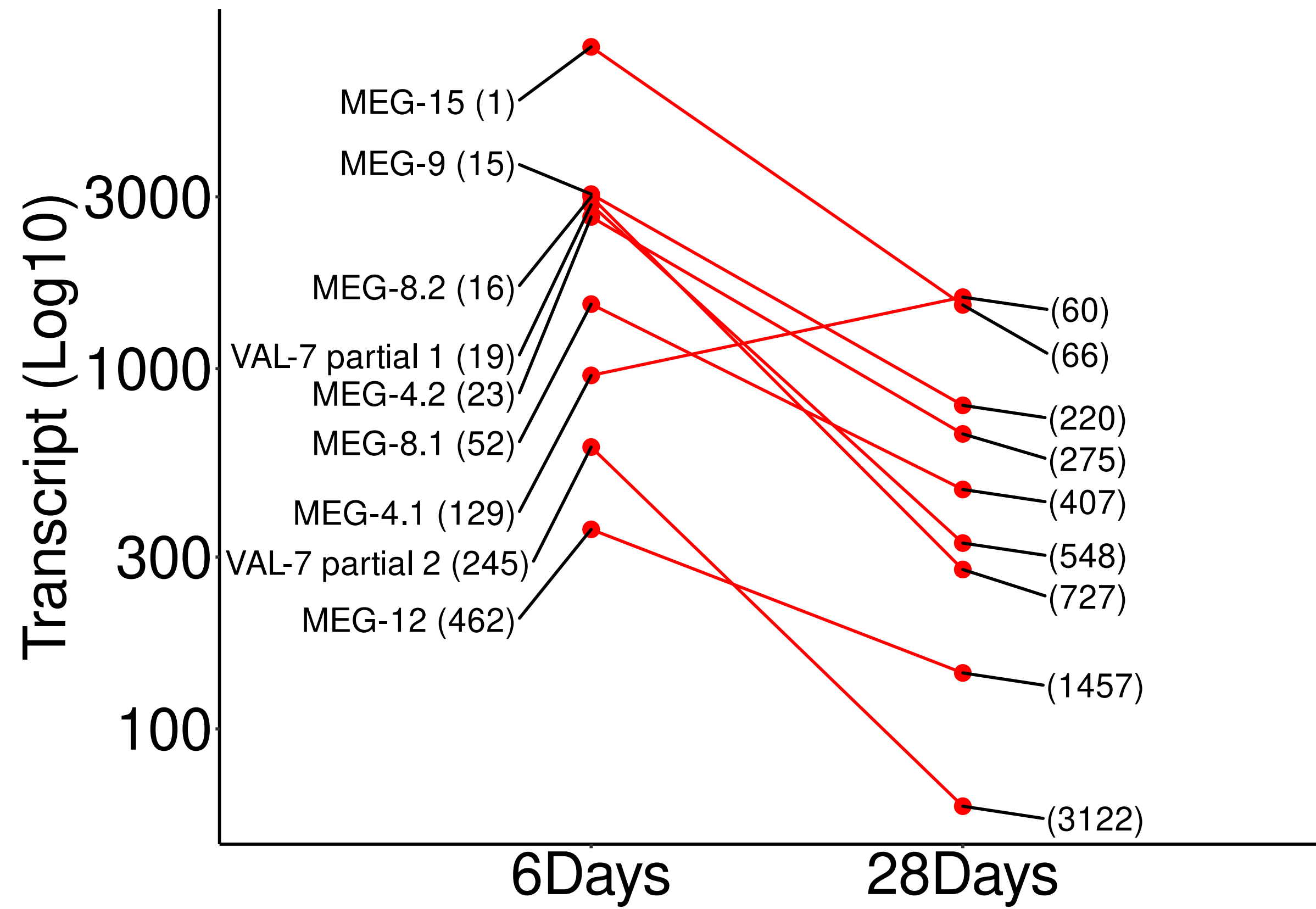

## Gastrodermis 1

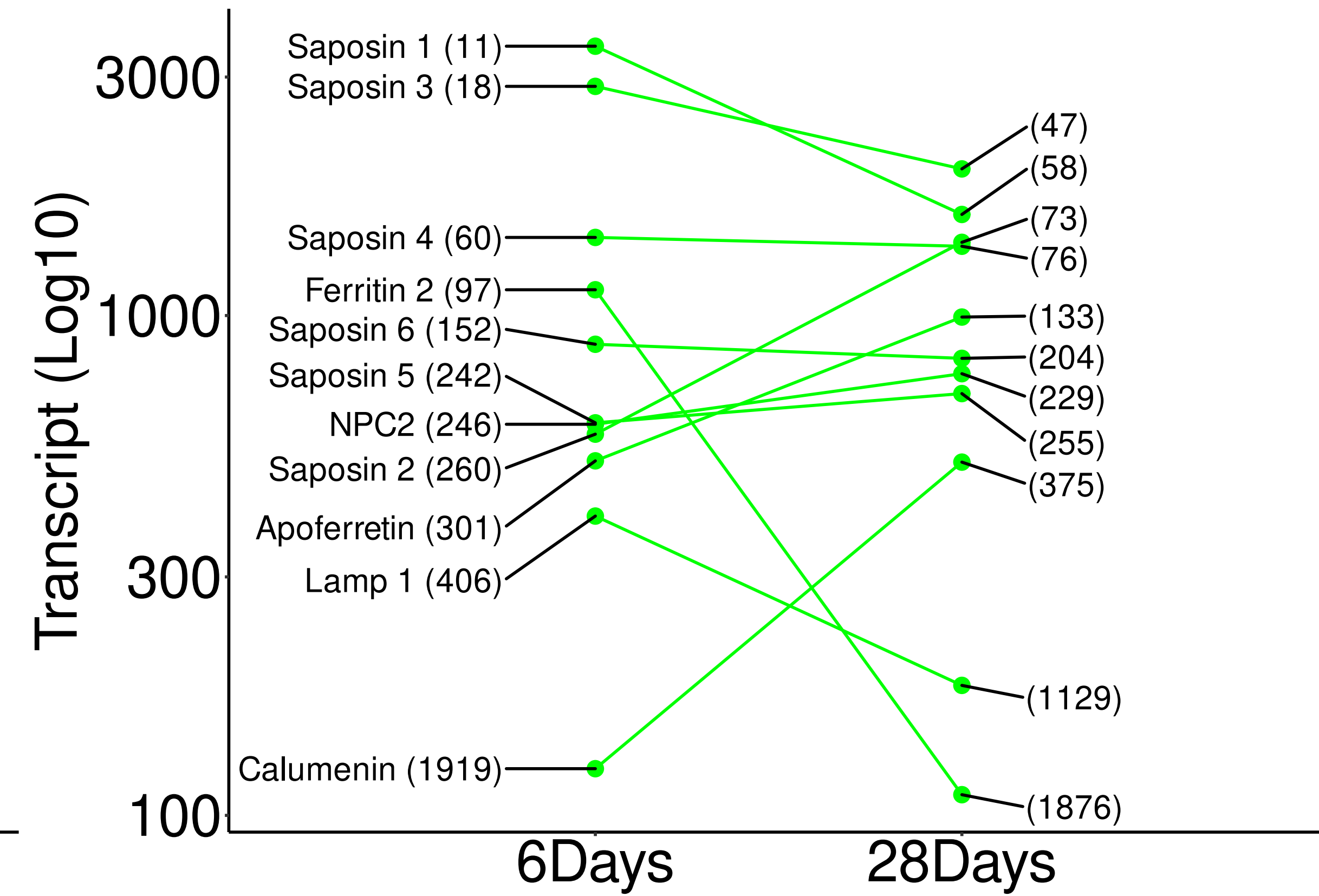

## Gastrodermis 2

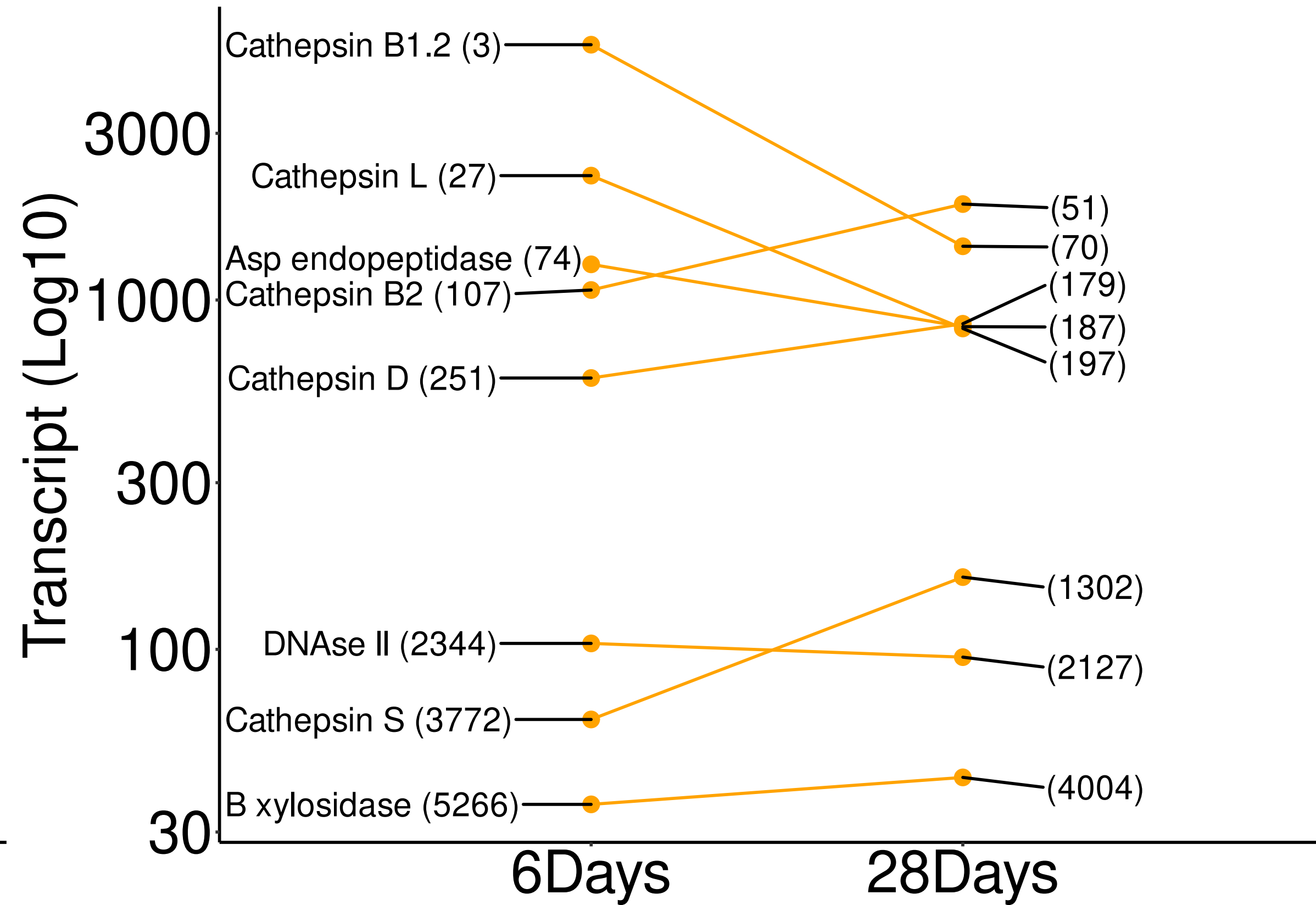

## Lung Stage

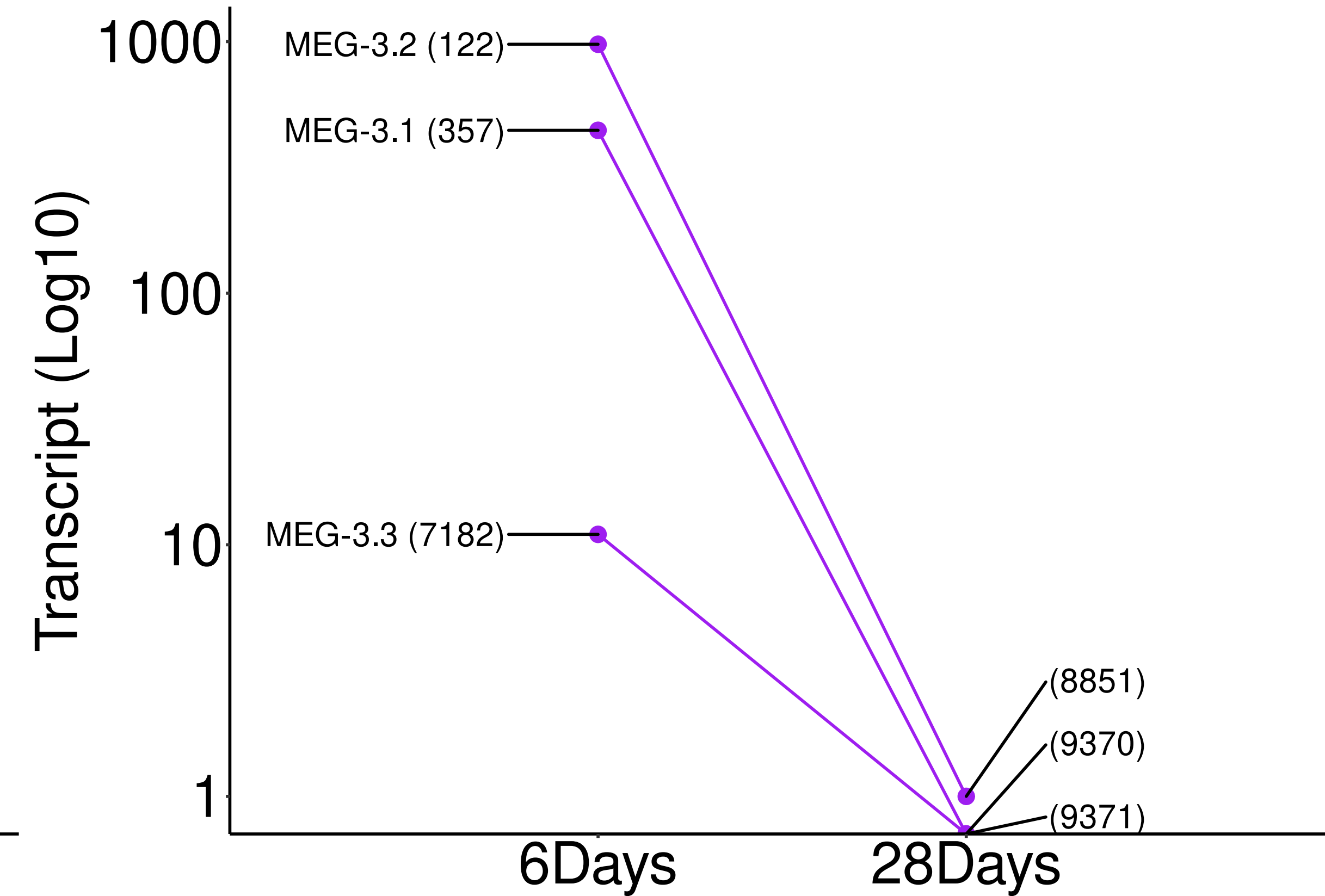

Supplement: Supplementary file 1 [file DataSheet_1.zip › Supplementary Material/Supplementary Figure 6.PDF]
